# Mining social mixing patterns for infectious disease models based on a two-day population survey in Belgium

**DOI:** 10.1186/1471-2334-9-5

**Published:** 2009-01-20

**Authors:** Niel Hens, Nele Goeyvaerts, Marc Aerts, Ziv Shkedy, Pierre Van Damme, Philippe Beutels

**Affiliations:** 1 Interuniversity Institute for Biostatistics and Statistical Bioinformatics (I-Biostat), Hasselt University and Catholic University of Leuven, Agoralaan 1, 3590 Diepenbeek, Belgium; 2 Centre for Health Economics Research & Modeling Infectious Diseases (CHERMID), Center for the Evaluation of Vaccination (CEV), Vaccine & Infectious Disease Institute (VAXINFECTIO), University of Antwerp, Belgium

## Abstract

**Background:**

Until recently, mathematical models of person to person infectious diseases transmission had to make assumptions on transmissions enabled by personal contacts by estimating the so-called WAIFW-matrix. In order to better inform such estimates, a population based contact survey has been carried out in Belgium over the period March-May 2006. In contrast to other European surveys conducted simultaneously, each respondent recorded contacts over two days. Special attention was given to holiday periods, and respondents with large numbers of professional contacts.

**Methods:**

Participants kept a paper diary with information on their contacts over two different days. A contact was defined as a two-way conversation of at least three words in each others proximity. The contact information included the age of the contact, gender, location, duration, frequency, and whether or not touching was involved.

For data analysis, we used association rules and classification trees. Weighted generalized estimating equations were used to analyze contact frequency while accounting for the correlation between contacts reported on the two different days.

A contact surface, expressing the average number of contacts between persons of different ages was obtained by a bivariate smoothing approach and the relation to the so-called next-generation matrix was established.

**Results:**

People mostly mixed with people of similar age, or with their offspring, their parents and their grandparents. By imputing professional contacts, the average number of daily contacts increased from 11.84 to 15.70. The number of reported contacts depended heavily on the household size, class size for children and number of professional contacts for adults. Adults living with children had on average 2 daily contacts more than adults living without children. In the holiday period, the daily contact frequency for children and adolescents decreased with about 19% while a similar observation is made for adults in the weekend. These findings can be used to estimate the impact of school closure.

**Conclusion:**

We conducted a diary based contact survey in Belgium to gain insights in social interactions relevant to the spread of infectious diseases. The resulting contact patterns are useful to improve estimating crucial parameters for infectious disease transmission models.

## Background

Mathematical models of person to person transmission of infectious agents require assumptions of social contact patterns between these persons. Such assumptions, in combination with the age-specific cumulative incidence of infection (for instance based on seroprevalence data [1-4]), enable estimating the so-called "Who Acquires Infection From Whom" (WAIFW) matrix [1]. The WAIFW matrix is central to dynamic transmission models, as are closely related parameters like the basic reproduction number and the force of infection. Over the last decade, some small and unrepresentative studies have explored ways of collecting data on social mixing behaviour relevant to the spread of close contact infections [5-9]. Alternatively, mixing patterns between age groups were estimated in a social network context [10]. Recently, the European commission project POLYMOD published the methods and broadly comparative results of large and representative population based surveys on social contacts recorded on a randomly assigned day in 8 countries (Belgium (BE), England & Wales (EW), Finland (FI), Germany (DE), Italy (IT), Luxemburg (LU), Poland (PL) and The Netherlands (NL)) [11].

The POLYMOD contact survey from Belgium differed from the surveys conducted in the other countries in several aspects. First, each participant registered contacts during 2 random days (one week day and one weekend day), enabling more refined coupled analyses on weekend versus week days. Second, participants could be linked to their geographical location enabling the comparison of contact behavior between regions. Third, a school holiday period was included in the sampling period, enabling more refined analyses on weekend versus holiday period days, and different simulations of the effects of school closure. Fourth, people with more than 20 professional contacts were requested to estimate these separately in terms of number and age to reduce reporting bias for persons with many professional contacts.

In the current paper, we aim to describe and analyze the complete Belgian contact survey in depth. The next section outlines the data collection procedure. We then explain the different methods of data analysis applied in this paper, starting from standard data mining techniques to simulations of the spread of newly emerging infections and the impact of school closure.

Finally, we end with a discussion on how these contact data can be used to improve estimation of infectious disease parameters.

## Methods

### Data collection

Over the period March – May 2006, 750 persons living in Belgium were recruited by random digit dialing on fixed telephone lines. Only one person per household could participate. In line with the total Belgian population (Chi-square test for equality of distributions, p-value 0.85), participants were recruited from the Flemish (n = 441), Walloon (n = 239) and Brussels geographic regions (n = 70), making sure also that the areas in these regions were representative of the overall urban and rural divide in Belgium. With regards to age, sampling was undertaken in order to obtain an age distribution with 10% in each of the age groups: 0–4, 5–9, 10–14, 15–19 years, and 6% in each of the adult age groups 20–24, 25–29, 30–34, 35–39, 40–44, 45–49, 50–54, 55–59, 60–64, 65+ years. Most (480) participants were adults aged 18 years and older of whom 50% were male, representative for the Belgian population (p-value 0.70). There were also 130 teenage participants aged 9–17 years (49% males, p-value 0.60) and 140 children aged 0–8 years (56% males, p-value 0.053).

The contact survey conducted as part of this study required participants to complete a survey anonymously, without changing their usual behaviour. No persons were subject to interventions and no physical samples were collected as part of this study. The study protocol was approved by the ethical committee of the Antwerp University Hospital.

Two types of physical contacts were defined: (1) two-way conversations during which at least three words were spoken; (2) contacts which involved skin to skin touching. Each participant was asked to fill in a paper diary recording their contacts during one randomly assigned weekday and one randomly assigned day on the weekend (not always in that order). Teenagers (9–17 y) filled in a simplified version of the diary, and were closely followed up to anticipate interpretation problems (all surveyors had received identical training instructions to accomplish this). For the children (< 9 y) a parent or exceptionally another adult caregiver filled in the diary. In addition to a pilot test in Luxemburg, specific Belgian Dutch and French language versions of each type of diary were made and tested in Belgium (see also [6,11]).

Information recorded in the diary included the exact or estimated age and gender of each contacted person, the duration of contacts per person over the entire day, frequency (habitual nature) and location or circumstance of contact (multiple options possible). A list of variables used for our analysis is given in Table 1.

**Table 1:** Classification Tree Variables.

Variables	Code	Abbreviation	Tree Usage
Physical Contact	1 = 'yes'; 2 = 'no'	touching	1*
Location	1 = 'home'; 2 = 'work'; 3 = 'school', 4 = 'transport'; 5 = 'leisure'; 6 = 'other-multiple'	location	1,2*,3,4
Frequency	1 = 'daily'; 2 = 'weekly'; 3 = 'monthly'; 4 = 'few times a year'; 5 = 'first time'	frequency	3*
Duration	1 = '0–5 min'; 2 = '5–15 min'; 3 = '15 min – 1 hour'; 4 = '1–4 hours'; 5 = '> 4 hours'	duration	4*
Age Contact	Continuous	agecon	1,2,3,4
Gender Contact	1 = 'male'; 2 = 'female'	gencon	1,2,3,4
Age Participant	Continuous	agepar	1,2,3,4
Gender Participant	1 = 'male'; 2 = 'female'	genpar	1,2,3,4
Occupation Participant	1 = 'working'; 2 = 'retired'; 3 = 'at home'; 4 = 'unemployed/job seeking'; 5 = 'in education'; 6 = 'other'	occpar	1,2,3,4
Household Size Participant	Household Size (including participant)	hhsize	1,2,3,4
Region	Brss = 'Brussels' WlsG = 'Walloon region' VlsG = 'Flanders'	region	1,2,3,4
Day of the Week	0 = 'Sunday'; 1 = 'Monday'; 2 = 'Tuesday'; 3 = 'Wednesday'; 4 = 'Thursday'; 5 = 'Friday'; 6 = 'Saturday'	dayofweek	1,2,3,4
Holiday Period	0 = 'no holiday period' 1 = 'holiday period';	holiday	1,2,3,4

Variables used in classification tree analyses. 'Tree usage' refers to the use of the variables in the tree construction for tree 1: physical contact; 2: contact location; 3: contact frequency and 4: contact duration. '*' indicates that the variable was used as the response variable.

The diaries were sent and collected by mail. Each participant was reminded by phone that they had to fill in the diary, one day prior to each assigned day, and was followed up after the first day to check whether they had. If they had not filled in the diary, they were assigned to a new random day. If they had not returned the diary, they were reminded by a maximum of three follow up calls to send it in. If participants repeatedly failed to fill in the diary on their assigned day, they were excluded, and replaced by a new recruit. Each participant received a small token of appreciation for the amount of 5 Euro. All diaries were double entered in a computer database and checked manually.

Virtually all (98%) participants recorded their contacts in the four weeks from March 18 to April 14 2006, and 49% and 73% of the recorded week days and weekend days, respectively, were during the Eastern holiday period. Note that weekends at the start and the end of the holiday period were considered as holiday and represented 72% of all recorded weekend days.

About half the children aged 0–2 attended childcare (55%), and almost all children aged 3–8 years attended childcare or school (93%), while school participation was 100% for participants aged 8–17 years. Adults aged 18 years or older were mostly employed (49%), unemployed (35%) or in further education (9%). Overall, 10% of the participants lived alone and these were all 18 years or older. Nearly 1 in 5 children aged 0–8 years lived in a single parent family, and 26%, 28% and 11% of the participants lived in a household of size 2, 3 and 4, respectively. Larger household sizes were only rarely observed (4%). These characteristics of the sample are all broadly in line with general Belgian population statistics (National Institute for Statistics, 2006, Belgium).

### Household contacts

Although in order to keep the diary manageable, we intentionally did not record the relationship with the contacted persons, we extracted household-like contact data from the database by identifying those contacts with the same age as the registered ages of the household members (knowing that the contacts occurred at home and that an exact age was given, and not an estimated age range as for the ages of contacted persons from whom the exact age is unknown). We performed a sensitivity analysis with respect to the selected contacts of the same age.

### Work contacts

As mentioned in the introduction, only a subsample of the Belgian contact survey (BE), was described and analyzed by [11], together with those from the seven other POLYMOD countries. These contact surveys, although broadly aiming to record similar outcomes, were not quite the same due to dissimilar implementations in each of the countries (see [11]). One important difference between these surveys was the registration of work contacts.

In half of the POLYMOD surveys (namely BE, DE, FI and NL), a threshold value was set for reporting contacts at work, requiring people with more estimated work contacts than a predefined threshold not to record these contacts in their diary. In the Belgian diaries, the participants were asked prior to filling in their dairy for the first time the following sequence of questions. (1) Do you have a profession through which you have a large number of contacts (e.g., clients, patients, students, etc)? YES/NO; (2) If YES, please make an estimate of the average number of persons you contact professionally per day? .... persons; (3) Please tick in which of these age categories these professional contacts mostly occur (multiple options possible): 0–5 y, 6–11 y, 12–17 y, 18–60 y and over 60 years. (4) If you estimated the number of these contacts at more than 20, then do not record these contacts in the diary, but only record the other (non-professional) contacts. The complete Belgian surveys (including diaries) are available [see Additional file 1, 2, 3, 4, 5, 6 and 7].

Although these questions were asked with the common intention to reduce reporting bias for people with many professional contacts (eg bus drivers), there are some important differences in how we approached this, in comparison to DE, FI and NL. First, based on information from the pilot studies, we set the threshold value at 20, whereas in DE, FI and NL it was set at 10. Second, we asked first if the participants thought they had many professional contacts, and only if they did subjectively think so, how many they estimated these to be. Third, we only revealed in the last question what the consequence of their estimate was for the effort required to complete the diary. Fourth, we did not only ask about the number of professional contacts, but also about their usual age range.

In reporting the results of all the POLYMOD diaries combined, [11] excluded these extra non-recorded contacts, thus presenting, in this respect, underestimates for the number of contacts in BE, DE, FI and NL, in comparison to the other 4 countries. In the current paper, we used imputation to complete the full Belgian data set. However, this too had its limitations, since imputation enabled generating data from which reliable inferences can be made, but could not recreate the values that were not recorded [12].

More specifically, the age of contacted persons provided a basis for imputation [see Additional file 7]: Consider $n_{i}^{w}$ > 20 as the number of work contacts to be imputed for participant *i *and (s)he indicated that these professional contacts were in specific age-categories denoted by a set $I_{i}^{a}$ (e.g. $I_{i}^{a}$ = [6,11] for a primary school teacher). We sampled $n_{i}^{w}$ age-values from $I_{i}^{a}$ with probabilities according to the population age-distribution. Contrasting this method with data for EW, IT, LU and PL indicated good performance.

In order to impute the other contact characteristics, plausible assumptions were made based on the available information from other POLYMOD countries. Mossong et al. [11] did not find a significant association between age and whether contacts involved skin-to-skin touching for EW, IT, LU and PL. Therefore we imputed, this variable in the Belgian dataset using the same distribution as when 10 <$n_{i}^{w}$ ≤ 20. The imputation of gender of contacts was performed based on the same reasoning. For the imputation of contact characteristics like duration and usual frequency, we considered it unlikely that a single professional contact in a large set of such contacts would last longer than 4 hours and reoccur daily. Therefore, we imputed these two variables jointly by sampling from the bivariate distribution of duration and frequency for work contacts of participants for whom 10 <$n_{i}^{w}$ ≤ 20. This method could also be more widely applied to all characteristics in an attempt to avoid disrupting dependencies, but could just the same also enforce dependencies. Whereas the imputation for age and gender was well founded (negligible change in distribution with $n_{i}^{w}$), other imputations seemed more speculative. Indeed, the higher the recorded $n_{i}^{w}$ was, the shorter the contact durations were, but the distribution of contact frequency remained quite stable. Furthermore, the choice of distribution for the duration of contacts would be subjective since (a) it was clearly unknown for what was missing and (b) the distributions varied substantially between countries. For the remainder of the paper, we focus on this augmented data set but note that the results were similar whenever these imputations were left out, except when estimating the number of contacts. This will be illustrated for one of the methods introduced in the next section.

### Statistical methodology

Since the contact survey contains a lot of information, we will, next to some descriptive statistics, use up-to-date statistical methodology to highlight different aspects from the data. We will first describe the use of association rules and classification trees to identify associations and factors related to type, location, frequency and duration of contact. Then we will use weighted generalized estimating equations to properly model the number of contacts taking into account the correlation of contacts recorded by the same participant on two different days. Finally, we relate contact patterns to the spread of infectious disease using the next generation matrix. While this methodology provides more insight into how close contact infectious diseases are spread, we note that we do not aim to describe the stochastic nature of an emerging epidemic.

In this section we present some more detail on the methods used throughout this manuscript and refer to the literature for an extensive description.

### Association rules

In large databases, like the contact database, patterns between variables can be discovered by data mining techniques such as association rules [13]. If *A*, *B *denote properties of contacts (e.g., frequency, location, touching), then association rules would focus on relations *A *→ *B *that estimate how likely the event *B *is given the occurrence of event *A*. While *A *could consist of more than one contact property (e.g. a home contact which also involved skin-to-skin touching), *B *is restricted to be a single property. The length of a rule is the total number of properties constituting that rule. Since numerous combinations of contact properties exist, various interestingness measures could be studied. We focused on the support, the confidence and the lift of a rule. The support of a rule expresses the relative frequency of all contacts in that rule and searching for rules with a high support can be seen as a simplification of 'bump hunting' [14]. The confidence of a rule expresses the conditional probability associated with that rule *P*(*B*|*A*). Finding rules with high confidence is similar to finding rules with a high association between the items, as is finding rules with a high lift value. In the latter case, a higher lift value indicates that the support of the rule is higher than the product of the support of the items in that rule (expressing independence between items [14]).

### Classification trees

To gain more insight in factors determining contact intensity (physical contact, location, frequency and duration), the binary classification tree methodology as introduced by [15] was used. This is a recursive partitioning method which is nonparametric in nature, simple and intuitively appealing. At each step, the recursive partitioning algorithm determined an optimal cut off point such that all the contacts were split in two subpopulations to achieve high predictive classification with respect to the variable of interest; touching (yes or no), location (home, school, work, school, transport, leisure or other), frequency (daily, weekly, monthly, a few times a year or first time) and duration (0–5 min, 5–15 min, 15 min – 1 hour, 1–4 hours, more than 4 hours). The resulting subpopulations were split repeatedly until no additional partitioning was warranted: either a subpopulation contained only one class or it was too small to divide any further. The program Rpart [16], implemented in R, was used to generate the decision trees depicting the classification rules generated through recursive partitioning. When growing a tree equal misclassification costs were assigned to the categories of the response variable. Pruning of the trees (to correct for overtraining) was undertaken using the 1 SE rule described by [15] in combination with a maximal tree-depth of 4 layers. Error rates associated with these trees were determined by simple resubstitution, and a 10-fold cross-validation (CV) was used to evaluate the performance of the tree. Table 1 lists the variables used in the tree construction.

### Modelling the number of contacts

While [11] used a censored negative binomial distribution to relate the overall number of contacts to different participant characteristics, we used generalized estimating equations (GEE, [17]). This approach allowed us to account for the correlation between the number of contacts recorded by the same individual on the two different days. Weights were included in the analyses, in the first instance to adjust for relative differences in age and household size as compared to Belgian demographic data (source: EUROSTAT), and in the second instance to adjust for differences in sampling proportions with respect to weekdays and holiday periods.

Model building was done a priori, using a nonparametric method called 'random forests' [18]. Random forests were constructed using binary regression trees [15] which relate a response variable, the contact number, to explanatory variables in a similar way as with the classification trees. However, in regression trees, the response is continuous and the objective is to minimise the mean squared error (MSE). In view of the skewness of the distribution of the number of contacts, a log-transform was used. Random forests were constructed by joining several of these regression trees, each based on a random sample of the observations and the explanatory variables, to explicitly take into account the variability associated with the construction of a single tree. A by-product of this random forests methodology, the so-called 'variable importance' list, reflects how often a variable is used as a splitting-criterion. The variables with a high importance, more explicitly using a threshold of 5% increase in MSE, were then selected and used in further model building. We refer the reader to [18] for more details on how 'random forests' are constructed.

Using the most important variables from the 'random forests' importance list, one can opt to look at the interactions between them. Since this inevitably leads to sparse cells, we only analysed interactions with respect to weekdays and holidays and for the remainder only considered main effect terms. We then further reduced the model using a stepwise Poisson regression (with log-link) based on backward selection and the BIC-criterion [19] and finally applied the weighted GEE analysis.

This procedure is applied separately for the three different types of diaries and for all types of contacts, resulting in 57 analyses.

### Who mixes with whom?

Consider the random variable *Y*_*ij*_, i.e. the number of contacts in age class *j *during one day as reported by a respondent in age class *i*, which has observed values *y*_*ij*, *t *_= 1,..., *T*_*i*_, where *T*_*i *_denotes the number of participants in the contact survey belonging to age class *i*. The elements *m*_*ij *_= *E*(*Y*_*ij*_) make up a *J *× *J *matrix, which is called the 'social contact matrix'. Now, the contact rates *c*_*ij *_are related to the social contact matrix as follows:

(1) $$c_{ij}=365\cdot \frac{m_{ji}}{w_{i}}$$

where *w*_*i *_denotes the population size in age class *i*, obtained from demographic data (EUROSTAT). When estimating the social contact matrix, the reciprocal nature of contacts needs to be taken into account (see [5]): *m*_*ij*_*w*_*i *_= *m*_*ji*_*w*_*j*_, which means that the total number of contacts from age class *i *to age class *j *must equal the total number of contacts from age class *j *to age class *i*. We used a negative binomial distribution with mean *m*_*ij *_and variance $m_{ij}+\frac{m_{ij}^{2}}{k}$ to model the number of contacts, while allowing overdispersion. The following negative binomial likelihood function was maximized in order to estimate the means *m*_*ij *_and the dispersion parameters *k*, assuming the *Y*_*ij *_were independent (*i*=1,...,*I*,*j *= 1,..., *J*):

(2) $$\prod_{i=1}^{I}\prod_{j=1}^{J}\prod_{t=1}^{T_{i}}{\left[\text{NegBin}(y_{ij,t};m_{ij},k)\right]}^{w_{it}^{d}},$$

where $w_{it}^{d}$ is the diary weight of the *t*^*th *^participant in age class *i*. In order to achieve the necessary flexibility and to exploit the continuous nature of the data, we estimated the mean contact rate using one-year age-intervals and a bivariate smoothing approach as described by Wood [20]. The basis was a tensor-product spline ensuring flexibility when modeling the average number of contacts as a function of the respondent's and contact's age. By applying a smooth-then-constrain-approach as proposed by [21], the reciprocal nature of contacts was taken into account.

### Mimicking the Spread of an Epidemic and the Impact of School Closure

In order to mimic the initial spread of a newly emerging pathogen in Belgium, we used the next generation operator as defined in (3).

(3) $$G[i](a)=\frac{ND}{L}\exp\left\{-\int_{0}^{a}\mu (t)dt\right\}\int_{0}^{+\infty }\beta (a,a^{\prime })i(a^{\prime })da^{\prime },$$

For Belgium, population size (*N *= 10 547 958), life expectancy at birth (*L *= 80) and age-specific mortality rates, μ(*t*) were obtained from official government statistics (source: EUROSTAT). The infectious period was set to five days *D *= 5/365, similar to the infectious period typically estimated for influenza (see e.g. [22]). We assumed *β*(*a*, *a*'), i.e. the effective transmission rate was proportional to the contact rate *c*(*a*, *a*') as estimated by *c*_*ij *_using one-year age-intervals and contacts involving skin-to-skin touching only and chose the proportionality factor such that the largest Eigen value of the next generation operator (3), i.e. the basic reproduction number *R*_0_, equaled 2 (thus mimicking the emergence of pandemic influenza [23,24]). Note that daily contacts were down weighted with a factor 1/5 to reflect the unlikely occurrence of transmission of infections during a second such contact. We first focused on a regular period and used the leading eigenvector to simulate the initial spread of a newly emerging pathogen [25]. We investigated the effect of school closure by using contact rates from the non-holiday period, the holiday period and in the weekend (excluding holidays), and comparing the resulting relative impact on *R*_0_.

## Results

**Figure 1:**
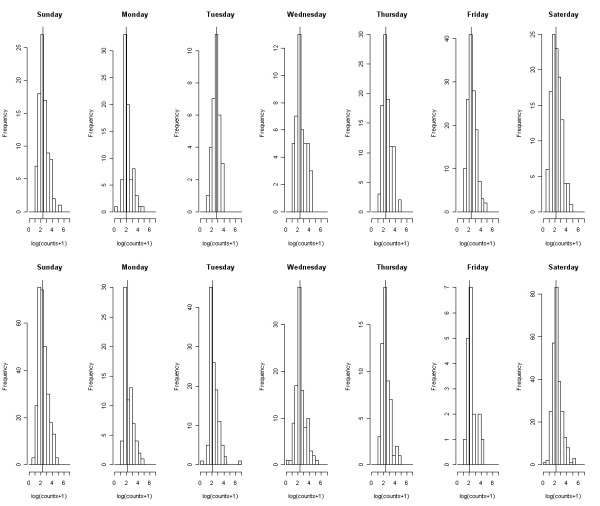
**Contact number densities per weekday**. Histograms of log(number of contacts+1) per day in the week, distinguishing non-holiday (top row) from holiday (bottom row) periods.

**Figure 2:**
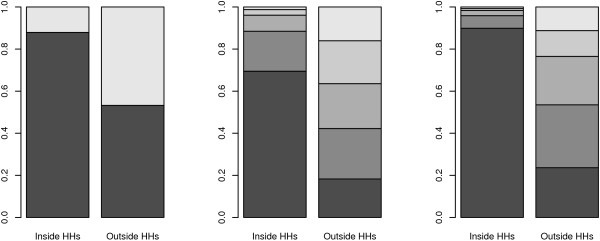
**Contact intensity distributions in- and outside households**. Contact intensity distributions in- and outside households: close or non-close (left figure); duration (middle panel); frequency (right panel). Darker colors correspond to touching, longer duration and more frequent contacts.

**Figure 3:**
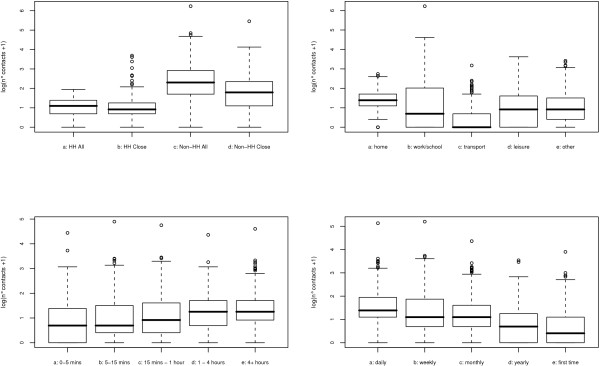
**Contact number densities for different contact characteristics**. Boxplots of the number of (close) contacts in and outside households (left upper panel); the number of contacts per location (right upper panel); the number of contacts for the different frequencies (left lower panel) and duration (right lower panel).

Figure 1 shows the histograms of the number of contacts on a log-scale per day in the week, distinguishing between holiday and non-holiday periods. The densities (especially on the original scale) were highly skewed, and more so for the holiday period. Median values were 7–9 for the holiday period and 9–12 for the non-holiday period. Figure 2 shows contact intensity distributions in- and outside households: close or non-close (left figure); duration (middle panel); frequency (right panel), clearly indicating that most intense contacts (darker shading) take place with household members. As a final descriptive statistic, Figure 3 shows boxplots of the number of (close) contacts in and outside households (left upper panel); the number of contacts per location (right upper panel); the number of contacts for the different frequencies (left lower panel) and duration (right lower panel). Whereas most contacts inside a household involve skin-to-skin touching, this is not true outside the household. There is substantial heterogeneity in the number of contacts recorded at work/school and during leisure activities with a high number of contacts mostly observed at work/school. Although there are no apparent differences, the lower left panel in Figure 3 indicates that there is a larger number of contacts with longer duration. Note that most contacts are frequent contacts whereas fewer contacts are yearly or first time contacts.

### Association rules

Overall, 61% of the reported contacts involved skin-to-skin touching; 26% lasted longer than 4 hours; and 31% occurred on a daily basis. Rules were only considered as interesting whenever the support exceeded 2% (≈475 contacts). When considering rules of length 2, more than 72% of the long duration contacts (more than 1 hour), home contacts and daily contacts, respectively, involved skin-to-skin touching. At the same time, more than 74% of the first time contacts of short duration (less than 5 minutes) were conversations without touching. Contacts lasting at least 4 hours, contacts at school and at home were usually daily contacts (confidence 0.62, lift 1.7). Rules of length 3 indicated the association between daily contacts lasting 4 hours or more and involving skin-to-skin touching. Also long contacts during leisure activities, at school or indicated to occur at "another" place almost certainly involved skin-to-skin touching (confidence 91%). Similarly as indicated by the rules of length 2, first time contacts lasting less than 5 minutes were mostly non-physical (confidence 86%). Single person households did not report long duration contacts at home. Transport contacts did not last longer than 4 hours.

When considering age-categories, contacts between children and contacts between adults and infants were mostly physical (75%).

### Classification trees

**Figure 4:**
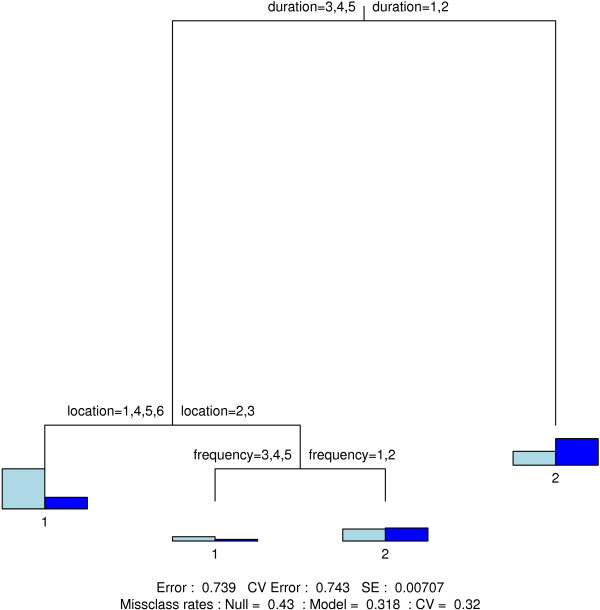
**Close or non-close contact classification tree**. Classification tree for contacts, involving skin to skin touching. Variable codes can be found in Table 1.

**Figure 5:**
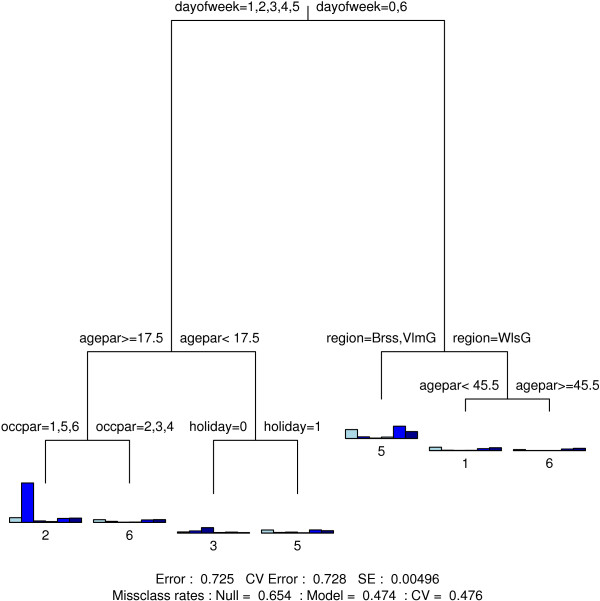
**Contact location classification tree**. Classification tree for contact location. Variable codes can be found in Table 1.

**Figure 6:**
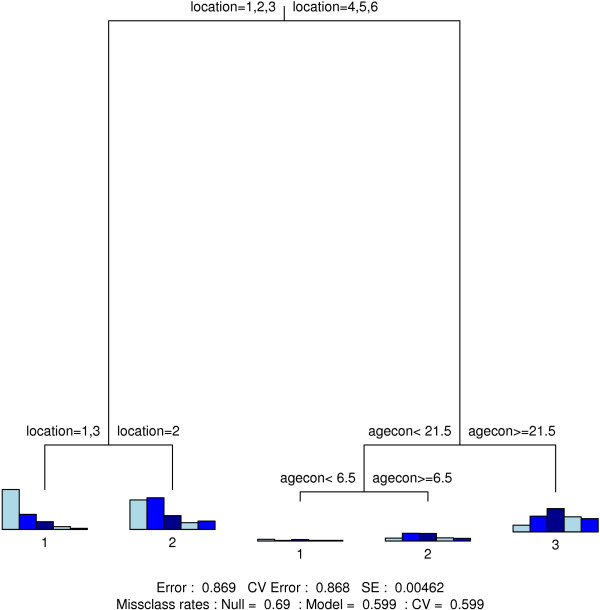
**Contact frequency classification tree**. Classification tree for contact frequency. Variable codes can be found in Table 1.

**Figure 7:**
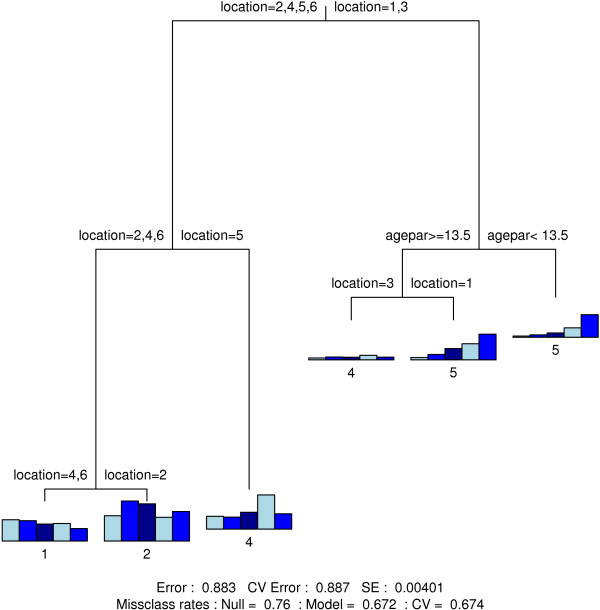
**Contact duration classification tree**. Classification tree for contact duration. Variable codes can be found in Table 1.

Figure 4, 5, 6 and 7 show the final classification trees for touching, contact location, contact frequency and contact duration, respectively. All trees showed an improvement with respect to misclassification compared to the Null model, i.e. a tree with only a root node. In general, the misclassification rate was still considerably high due to several heterogeneous terminal nodes. The CV-values were close to or approximately equal to the resubstitution misclassification rate. The length of a branch indicates its relative importance versus other branches. Together with the split variable; frequency plots for the final nodes (nodes without further subdivision) are shown. In addition, classification trees based on the non-augmented data were found indifferent [see Additional files 8, 9, 10 and 11].

Duration, location and contact frequency with the contacted person, determined mainly whether or not a contact involved touching (Figure 4). Two thirds of contacts of shorter duration occurred without touching, while longer contacts taking place at home, during transportation or leisure activities usually involved touching (78%). At work and school, there was a fairly even balance between touch and non-touch contacts (53% and 47%, respectively).

On weekdays, contacts mainly occur at work (adults) or at school (children-adolescents) except for the holiday period where children and adolescents make more contacts at home and during leisure activities (Figure 5). In the weekend, contacts mainly take place at home and at leisure activities. Interestingly, there was a significant difference in the relative number of weekend contacts during leisure activities between the Walloon Region (25%) and the Flemish and Brussels region (39%) (p-value < 0.001).

Usual contact frequency was mainly determined by location and by the age of the contacted person (Figure 6). More frequent contacts were observed at home and school; and to a lesser extent at work. Contacts during transport and leisure activities were less frequent.

Contact duration was highly dependent on contact location (Figure 7). Long duration contacts mainly took place at home and at school, while shorter contacts took place during transport. Contacts at work typically constituted a mix of long and short duration contacts.

### Modelling the number of contacts

Table 2 shows the variables used for each of the diary types. For the resulting models for the total number of contacts: [see Additional file 12]. A significant decrease in the number of contacts made during the holiday period was observed for children and adolescents, reflecting school closure. Over the weekend there was a significant decrease in the number of contacts made by adolescents and adults but not by children. The number of children in childcare (and class size for the older children) is positively correlated with the number of contacts made while there was no significant impact of the class size for adolescents on the total number of contacts. Household size was a significant factor for all age groups, while gender, although retained in the Poisson regression step, turned out to have no significant impact. For the adults, the number of contacts was highly influenced by their occupation, with employees or students making twice as many contacts as unemployed or retired adults. Overall a significant level of overdispersion was observed in all models, but the correlations were not significantly different from zero.

**Table 2:** Weighted GEE variables.

	Children	Adolescents	Adults
Age	X	X	X
Childcare	X		
Class size		X	
Day of week*	X	X	X
Education			X
First or second day	X	X	X
Gender	X	X	X
Holiday	X	X	X
Household size	X	X	X
Participant occupation			X
Region	X	X	X
Weekend*	X	X	X

Variables used as initial variables in the weighted GEE analysis for each of the diary types (X indicates the inclusion of the variable). '*' indicates that the final model retained at maximum one of the two variables.

Disentangling the total number of contacts in terms of location, duration, frequency and whether they involved skin-to-skin touching, yielded interesting insights in contact behavior. Person to person transmission was generally more likely to occur during more intimate contacts. Such more intimate contacts can be defined as contacts involving touching, contacts of long duration or contacts on a daily/weekly basis. We observed the number of such contacts to increase significantly for children in larger households, and to decrease significantly during holiday periods. Adolescents experienced more intimate contacts with increasing household size, class size and when living in Flanders. Work was the key factor for adults, where almost a two-fold increase in the number of intimate contacts for working adults was observed. In addition to the positive correlation with household size (as for children and adolescents), there was a marked increased number of more intimate contacts for adults living in Flanders and the Walloon region as opposed to the Brussels region.

In general, there was no significant difference in contact intensity when comparing males and females, nor was there a difference among the different days of the week preceding the weekend. However, there was a large distinction between week and weekend days with most contacts occurring during week days. Adults who obtained their highest degree in secondary school make significantly more non-touch contacts than others. Furthermore, the number of leisure contacts per person is higher in Flanders than in Wallonia and Brussels. The number of transport contacts for adolescents is lower in the holiday period.

The two-day setup of the contact survey was exploited by studying correlations. While there was no correlation between the total number of contacts reported over the two different days, positive correlations show up for categories specifically defining more intimate contacts. There was a slight, mostly non-significant decrease in the number of contacts reported at day two (irrespective of whether this was a weekend or a week day) which should be considered in further similar studies.

### Who mixes with whom?

**Figure 8:**
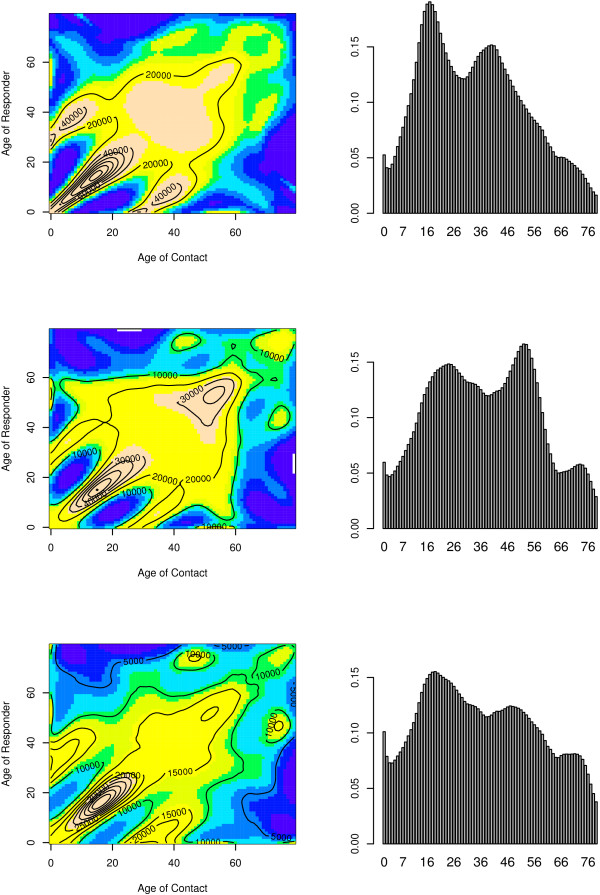
**Contact patterns and newly emerging infections**. Contact Patterns (left column) and leading eigenvector (right column) for a newly emerging infection in Belgium based on close contact patterns in a non-holiday period (first row), holiday period (middle row) and during weekends (excluding weekends during holidays) (last row). Contact patterns overlaid with contours are plotted at the population level.

Using the negative binomial model for the total number of close contacts a person of age *a *makes with persons of age *a' *in a non-holiday period resulted in the surface depicted in Figure 8 (upper left panel). Again as in the weighted GEE analyses, adjustment weights were applied to the distribution of week days and age and gender of the participants with respect to the Belgian population. We observed a clear assortative pattern in contact behavior, especially for children and adolescents, as well as a clear child-parent component. The assortativeness is less pronounced for adults, due to the more age diverse contact patterns in comparison to children and adolescents. Similar, though less assortative close contact patterns were constructed for the holiday period. Finally, the lower left panel in Figure 8 shows mixing patterns based on weekend days only. These patterns were used to simulate the effect of school closure on the spread of a newly emerging pathogen (see below).

### Living with children

**Figure 9:**
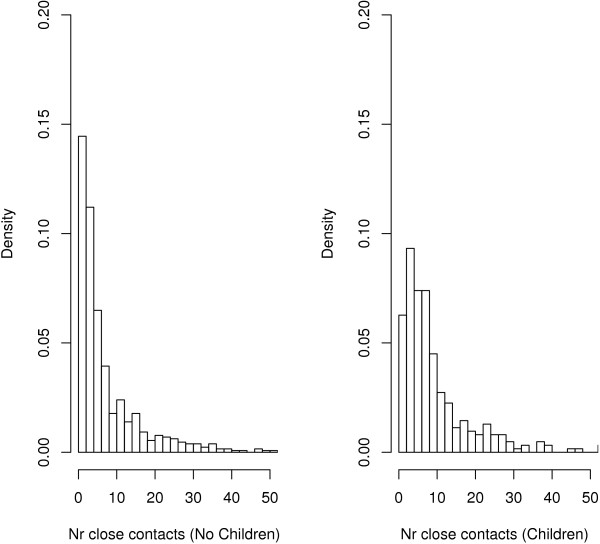
**Living with or without children and average daily number of close contacts**. Distribution of daily number of close contacts for adults living without and with children (left and right panel, respectively.).

Adults living with children are known to be exposed to a higher infection risk (see e.g. [26]). Figure 9 illustrates the difference in density of close contacts between adults living with and without children. A clear increase in the number of close contacts is observed for adults living with children (on average 10.4 as compared to 8.2).

### Mimicking the spread of an epidemic and the impact of school closure

The upper right panel in Figure 8 illustrates the leading eigenvector for a newly emerging infection during a non-holiday period, where the infectivity potential (i.e. the proportionality factor) was chosen so that *R*_0 _= 2.00. The highest relative incidence is observed for the age-group 14–20 years while a second local maximum was observed for persons aged 35–40 years. To simulate school closure, we calculated the relative incidence based on contact patterns as observed in the holiday period, while retaining the infectivity potential. This resulted in *R*_0 _= 1.69, a 15.5% reduction, as illustrated in the middle panel of Figure 8. While the relative incidence for adolescents decreased considerably, a shift in location for both local maxima is observed. In the holiday period grandparents seemed to play a more active role in that they made more contacts with their grandchildren. Alternatively, we simulate school closure by using weekend contacts only to construct the next generation matrix. In that case, we observed a substantial decrease in relative incidence for adults due to the assumed decrease in work contacts. This resulted in *R*_0 _= 1.33. These latter contact patterns do not necessarily provide a valid surrogate for holidays to estimate the impact of school closure, because the inter-age group mixing changes the expected incidence of infection by age, but they potentially do provide further insights when embedded in a sensitivity analysis.

## Discussion

In this paper, we described a large-scale Belgian contact survey, in which respondents recorded their contacts over two random days, one weekday and one weekend day, and estimated their professional contacts separately if these exceeded 20. Sampling in this survey was done by random digit dialing on fixed telephone lines and thus has its limitations with respect to the representativeness of the Belgian population, especially because of a substantial increase in the number mobile phone users without fixed telephone lines [27].

The association rules and classification tree analyses revealed logical results in that there were robust associations between general intimacy indicators of contacts, such as duration, contacts at home or during leisurely activities, and whether or not people touched. Furthermore contacts between children and contacts between adults and infants were found to usually involve touching.

Mossong et al.[11] analyzed a subset of this Belgian contact survey together with those from seven other European countries but did not take into account the separately recorded professional contacts. By explicitly imputing professional contacts on an individual level, the average number of contacts for the Belgian contact survey increased from 11.84 to 15.70. Due to various limitations in registering professional contacts in the other European country surveys, this difference should be borne in mind while interpreting the results reported in Mossong et al.[11].

## Conclusion

The number of reported contacts depended heavily on household size, class size for children (number of other children in childcare for infants) and the number of professional contacts for adults. Adults (mostly parents) living with children had on average 2 contacts more than adults living without children.

Differences in the average number of contacts were observed for the three regions in Belgium, showing the importance of considering regional differences. The correlation among the two days recorded was found to be significant for more intimate contacts.

People mostly mix with people of similar age, or with their offspring, their parents or their grandparents. In the holiday period the contact frequency for children and adolescents decreased with about 19% while a similar observation is made for adults in the weekend. The recorded contact behavior in the holiday period provided useful results to mimic the effect of school closure on the spread of airborne infections such as pandemic influenza. Indeed, we showed that weekends do not necessarily provide a valid surrogate for holidays to gauge these effects, as the inter-age group mixing changes the expected incidence of infection by age.

Finally, the resulting contact patterns could prove useful to improve the estimation of transmission parameters for airborne infections based on serological data as already shown by [5]. We note that for different diseases, different levels of contact intimacy will play a role. For example, Mycobacterium tuberculosis will be transmitted during more intimate contact as compared to the common cold or influenza. Therefore, contrasting different types of contact data to serological/incidence data in the spirit of [5] would substantiate empirical evidence about which type of contacts are most predictive for the spread of a specific infectious disease in a population. We refer to [28,29] for the explicit use of the Belgian contact data to estimate the transmission parameters and the basic reproduction number for the varicella zoster virus in Belgium. While [28] implemented these contact structures in a mathematical framework and compared it to the traditional WAIFW-matrices approach and the mass action principle, [29] focused on the statistical uncertainty in the estimation procedure and questioned the proportionality assumption on which [5] based their approach. Moreover, both [28,29] showed that contacts involving skin-to-skin touching and lasting at least 15 minutes were most predictive for the observed seroprevalence profile of varicella zoster virus in Belgium.

In addition to using the data we described in the current paper to define the WAIFW matrix in compartmental transmission models, it would seem useful to use these data also in network models where the spread of infectious diseases are mimicked using micro-simulation techniques.

## Competing interests

The authors declare that they have no competing interests.

## Authors' contributions

NH and PB drafted the manuscript in consultation with all the other authors; PB, PVD, MA and ZS designed and coordinated the survey. NH and NG conducted the data mining analyses, weighted GEE analyses, surface smoothing and epidemic modeling in consultation with MA, ZS and PB. All authors read and approved the final manuscript.

## Pre-publication history

The pre-publication history for this paper can be accessed here:

http://www.biomedcentral.com/1471-2334/9/5/prepub

## Supplementary Material

Additional file 1 **Diary Children Dutch.** original diaries in Dutch for children. Click here for file

Additional file 2 **Diary Children French.** original diaries in French for children. Click here for file

Additional file 3 **Diary Adolescents Dutch.** original diaries in **Dutch** for adolescents. Click here for file

Additional file 4 **Diary Adolescents French.** original diaries in French for adolescents. Click here for file

Additional file 5 **Diary Adults Dutch.** original diaries in **Dutch** for adults. Click here for file

Additional file 6 **Diary Adults French.** original diaries in French for adults. Click here for file

Additional file 7 **Data dictionary.** English data dictionary of the diaries. Click here for file

Additional file 8 **Figure 4 non-augmented.** Figure 4 for the non-augmented data. Click here for file

Additional file 9 **Figure 5 non-augmented.** Figure 5 for the non-augmented data. Click here for file

Additional file 10 **Figure 6 non-augmented.** Figure 6 for the non-augmented data. Click here for file

Additional file 11 **Figure 7 non-augmented.** Figure 7 for the non-augmented data. Click here for file

Additional file 12 **WGEE.** The results of the weighted GEE analyses. Click here for file
