# Supplementary material for: Mining social mixing patterns for infectious disease models based on a two-day population survey in Belgium
Source: BMC Infect Dis. 2009 Jan 20;9:5. doi: 10.1186/1471-2334-9-5 (PMC2656518; doi:10.1186/1471-2334-9-5)
Supplement: Additional file 1 — Diary Children Dutch. original diaries in Dutch for children. [file 1471-2334-9-5-S1.doc]

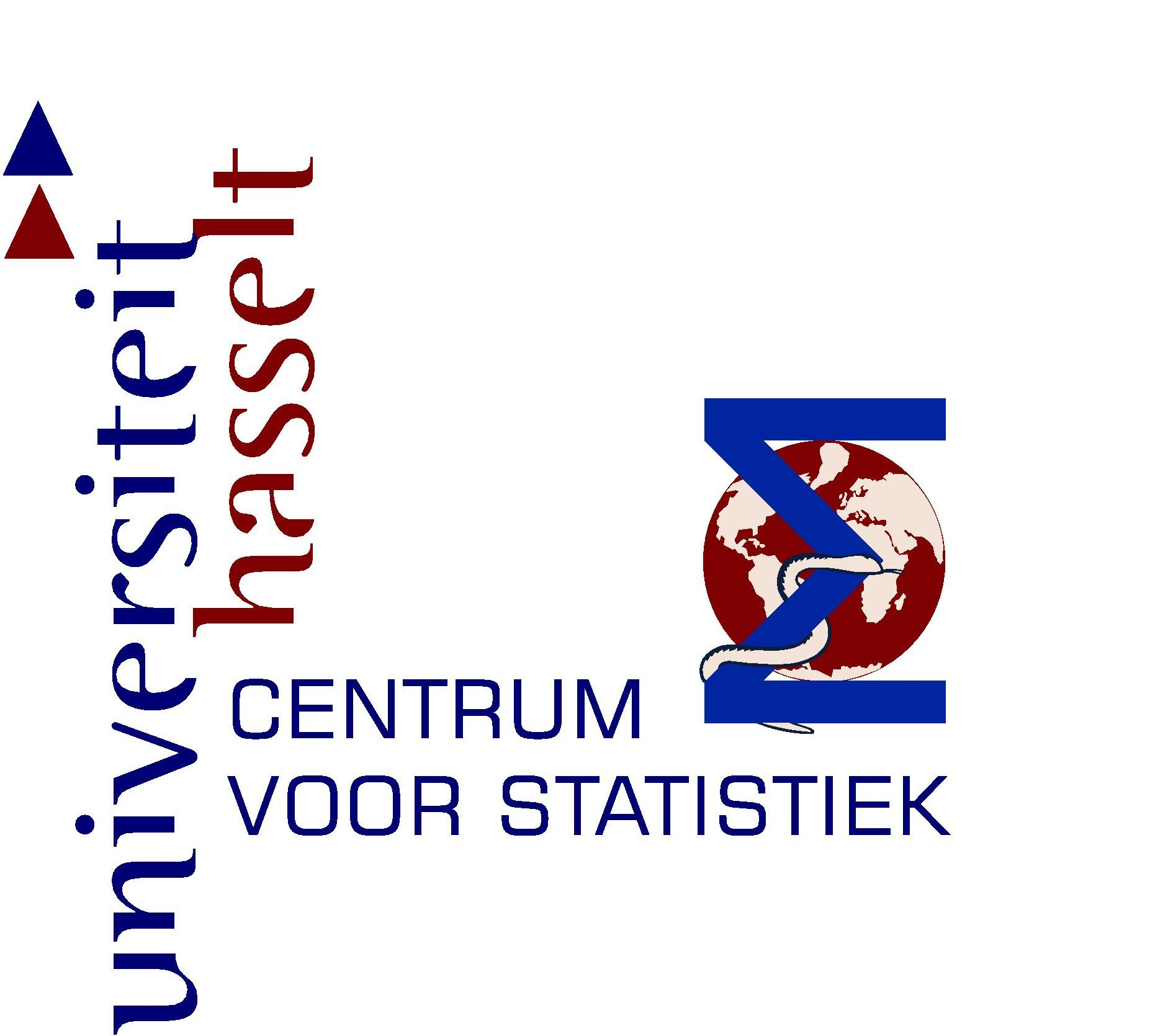


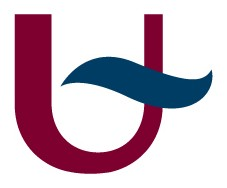


**Universiteit Antwerpen**


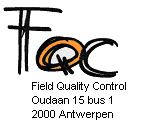


### Dagboekje

### studie contactpatronen

Indien u vragen hebt over het dagboekje, neem dan contact op met:

**03-231 06 67** of **0800-93667**

Marie-Paule Feremans – Dave Van Ginkel

#### Nr

##### Dag 1, datum / /

Dag 2, datum / /

**Instructies bij het invullen van het dagboekje**

- Gelieve alle personen met wie uw kind direct contact heeft gehad en die hij/zij deze 2 dagen ontmoet heeft, te noteren in het dagboekje.
- Met ‘contact’ bedoelen we dat uw kind met iemand gesproken heeft in zijn/haar aanwezigheid (geen telefonisch contact of contact via internet). Het contact kan ook fysiek zijn: iemand aanraken (hand geven, een kus geven, knuffelen, toevallige contacten tijdens sport).
  - Contact met dieren moet niet genoteerd worden.
  - Belangrijk: personen met wie uw kind enkel telefonisch contact (GSM/internet) heeft gehad, moeten niet opgenomen worden in het dagboekje.
  - Als u de exacte leeftijd van de persoon met wie uw kind contact had niet kent, geef dan een zo nauwkeurig mogelijke schatting (bv tussen 40 en 45 jaar).
  - Enkel de contacten van uw kind moeten in het dagboekje komen, niet uw eigen contacten. We stellen voor om het dagboekje samen met uw kind in te vullen om er zeker van te zijn wie hij/zij gezien heeft tijdens de dag.
- Gebruik 1 regel per persoon met wie uw kind contact had: als uw kind met een bepaalde persoon verschillende keren contact had, schrijf dit dan eenmaal op en geef een schatting van de totale tijd die ze samen doorbrachten.
- Het meest eenvoudige is de contacten van uw kind chronologisch in te vullen, te beginnen met het contact dat hij/zij het eerst had die dag en verdergaan met de andere personen die hij/zij zich herinnert in functie van de activiteiten van die dag.
- Als u denkt klaar te zijn met de lijst van contacten die uw kind gehad heeft die dag, overloop de dag dan nog eens grondig door na te kijken of u geen enkele activiteit vergeten bent waarbij uw kind contact gehad heeft. Een schoolagenda kan hierbij een hulp zijn.
- Een ‘dag’ beschouwen we in deze studie als één die begint om 5u ‘s morgens en eindigt om 5u de volgende morgen.
- Waar we bij de persoonlijke vragen verwijzen naar ‘het kind’, gaat het over het kind op wie dit dagboekje betrekking heeft.

Wij danken u voor het invullen van volgende persoonlijke gegevens over uzelf (ouder of andere volwassene):

1. Leeftijd jaar
2. Geslacht ⬜ vrouw ⬜ man
3. Welke band hebt u met het kind?
   - Ouder (stiefouder)
   - Andere familieband (oom, tante, grootouder, ...)
   - andere
4. Beroep
   - Zelfstandige (kunstenaar, handelaar, landbouwer, …)
   - Kader of vrij beroep (advocaat, dokter, architect, …)
   - bediende
   - arbeider
   - gepensioneerde
   - huisvrouw/man
   - student(e)
   - werkzoekend
   - andere
5. Opleidingsniveau (welke studies hebt u beëindigd):

⬜ geen enkele

⬜ basisonderwijs (lagere school)

⬜ beroepsonderwijs

⬜ lager technisch onderwijs

⬜ lager secundair onderwijs

⬜ hoger technisch onderwijs

⬜ hoger secundair onderwijs

⬜ hoger onderwijs buiten de universiteit

⬜ universitair onderwijs

1. Nationaliteit:
   - Belg
   - Andere nationaliteit binnen de Europese Unie
   - Andere nationaliteit buiten de Europese Unie
2. Aantal personen die deel uitmaken van het gezin (zonder het kind):
3. Leeftijd van de gezinsleden (zonder het kind), te beginnen met de jongste: , , , , , , , , , , ,
4. Woonplaats 10. Postcode

# Wij danken u voor het invullen van volgende gegevens over uw kind:

11. Leeftijd jaar

12. geslacht ⬜ vrouw ⬜ man

13. Gaat uw kind naar een crèche/peutertuin/school?

⬜ ja gemiddeld zijn er ⬜ < 10 kinderen in de crèche/peutertuin/school…

⬜ 10-20 kinderen in de crèche/peutertuin/school…

⬜ > 20 kinderen in de crèche/peutertuin/school…

⬜ neen

14. Nationaliteit

⬜ Belg

⬜ andere nationaliteit binnen de Europese Unie

⬜ andere nationaliteit buiten de Europese Unie

# Voorbeeld

| Leeftijd (of leeftijdscategorie) | Geslacht ♀ ♂  vrouw man | Plaats van contact (meerdere antwoorden mogelijk)  crèche,  peutertuin, onderweg  school, (auto,  thuis werk hogeschool trein  universiteit bus, ...) vrije tijd andere |
| --- | --- | --- |
| (- )  9  (- )  2  5  3  0 | X  X | X  X  X |

Eerste regel: uw kind heeft ’s morgens met zijn/haar broer van 9 gepraat terwijl ze samen naar school gingen met de schoolbus. ’s Avonds hebben ze samen gespeeld tussen 18h en 20h (ze hadden fysiek contact).

Tweede regel: Uw kind heeft met een jonge verkoopster gesproken in uw lievelings-schoenwinkel waar u een paar keer per jaar samen met hem/haar komt. Uw kind heeft vandaag verschillende schoenen gepast.

| Hoe dikwijls ziet u deze persoon?  (bijna) enkele enkele enkele  elke keren keren keren  dag per week per per jaar eerste  maand of minder keer | Hebt u hem/haar aangeraakt?  (bv hand geven, kus geven, sport)    ja neen | Tijd doorgebracht met deze persoon      Minder 5-15 15 min 1-4u 4u of  dan min -1u langer  5 min |
| --- | --- | --- |
| X  X | X  X | X  X |

Datum dag 1 / /

**Lijst van de personen met wie uw kind in contact is geweest tijdens**

| Leeftijd (of leeftijdscategorie) | Geslacht ♀ ♂  vrouw man | Plaats van contact (meerdere antwoorden mogelijk)  crèche,  peutertuin, onderweg  school, (auto,  thuis werk hogeschool trein  universiteit bus, ...) vrije tijd andere |
| --- | --- | --- |
| (- )  (- )  (- )  (- )  (- )  (- )  (- )  (- )  (- )  (- )  (- )  (- )  (- )  (- )  (- ) |  |  |

**deze eerste dag (van 5u ’s morgens tot 5u ’s morgens de volgende dag)**

| Hoe dikwijls ziet u deze persoon?  (bijna) enkele enkele enkele  elke keren keren keren  dag per week per per jaar eerste  maand of minder keer | Hebt u hem/haar aangeraakt?  (bv hand geven, kus geven, sport)    ja neen | Tijd doorgebracht met deze persoon    Minder 5-15 15 min 1-4u 4u of  dan min -1u langer  5 min |
| --- | --- | --- |
|  |  |  |

Datum dag 1 / /

**Lijst van de personen met wie uw kind in contact is geweest tijdens**

| Leeftijd (of leeftijdscategorie) | Geslacht ♀ ♂  vrouw man | Plaats van contact (meerdere antwoorden mogelijk)  crèche,  peutertuin, onderweg  school, (auto,  thuis werk hogeschool trein  universiteit bus, ...) vrije tijd andere |
| --- | --- | --- |
| (- )  (- )  (- )  (- )  (- )  (- )  (- )  (- )  (- )  (- )  (- )  (- )  (- )  (- )  (- ) |  |  |

**deze eerste dag (van 5u ’s morgens tot 5u ’s morgens de volgende dag)**

| Hoe dikwijls ziet u deze persoon?  (bijna) enkele enkele enkele  elke keren keren keren  dag per week per per jaar eerste  maand of minder keer | Hebt u hem/haar aangeraakt?  (bv hand geven, kus geven, sport)    ja neen | Tijd doorgebracht met deze persoon    Minder 5-15 15 min 1-4u 4u of  dan min -1u langer  5 min |
| --- | --- | --- |
|  |  |  |

Datum dag 1 / /

**Lijst van de personen met wie uw kind in contact is geweest tijdens**

| Leeftijd (of leeftijdscategorie) | Geslacht ♀ ♂  vrouw man | Plaats van contact (meerdere antwoorden mogelijk)  crèche,  peutertuin, onderweg  school, (auto,  thuis werk hogeschool trein  universiteit bus, ...) vrije tijd andere |
| --- | --- | --- |
| (- )  (- )  (- )  (- )  (- )  (- )  (- )  (- )  (- )  (- )  (- )  (- )  (- )  (- )  (- ) |  |  |

**deze eerste dag (van 5u ’s morgens tot 5u ’s morgens de volgende dag)**

| Hoe dikwijls ziet u deze persoon?  (bijna) enkele enkele enkele  elke keren keren keren  dag per week per per jaar eerste  maand of minder keer | Hebt u hem/haar aangeraakt?  (bv hand geven, kus geven, sport)    ja neen | Tijd doorgebracht met deze persoon    Minder 5-15 15 min 1-4u 4u of  dan min -1u langer  5 min |
| --- | --- | --- |
|  |  |  |

Datum dag 1 / /

**Lijst van de personen met wie uw kind in contact is geweest tijdens**

| Leeftijd (of leeftijdscategorie) | Geslacht ♀ ♂  vrouw man | Plaats van contact (meerdere antwoorden mogelijk)  crèche,  peutertuin, onderweg  school, (auto,  thuis werk hogeschool trein  universiteit bus, ...) vrije tijd andere |
| --- | --- | --- |
| (- )  (- )  (- )  (- )  (- )  (- )  (- )  (- )  (- )  (- )  (- )  (- )  (- )  (- )  (- ) |  |  |

**deze eerste dag (van 5u ’s morgens tot 5u ’s morgens de volgende dag)**

| Hoe dikwijls ziet u deze persoon?  (bijna) enkele enkele enkele  elke keren keren keren  dag per week per per jaar eerste  maand of minder keer | Hebt u hem/haar aangeraakt?  (bv hand geven, kus geven, sport)    ja neen | Tijd doorgebracht met deze persoon    Minder 5-15 15 min 1-4u 4u of  dan min -1u langer  5 min |
| --- | --- | --- |
|  |  |  |

Datum dag 1 / /

**Lijst van de personen met wie uw kind in contact is geweest tijdens**

| Leeftijd (of leeftijdscategorie) | Geslacht ♀ ♂  vrouw man | Plaats van contact (meerdere antwoorden mogelijk)  crèche,  peutertuin, onderweg  school, (auto,  thuis werk hogeschool trein  universiteit bus, ...) vrije tijd andere |
| --- | --- | --- |
| (- )  (- )  (- )  (- )  (- )  (- )  (- )  (- )  (- )  (- )  (- )  (- )  (- )  (- )  (- ) |  |  |

**deze eerste dag (van 5u ’s morgens tot 5u ’s morgens de volgende dag)**

| Hoe dikwijls ziet u deze persoon?  (bijna) enkele enkele enkele  elke keren keren keren  dag per week per per jaar eerste  maand of minder keer | Hebt u hem/haar aangeraakt?  (bv hand geven, kus geven, sport)    ja neen | Tijd doorgebracht met deze persoon    Minder 5-15 15 min 1-4u 4u of  dan min -1u langer  5 min |
| --- | --- | --- |
|  |  |  |

Datum dag 1 / /

**Lijst van de personen met wie uw kind in contact is geweest tijdens**

| Leeftijd (of leeftijdscategorie) | Geslacht ♀ ♂  vrouw man | Plaats van contact (meerdere antwoorden mogelijk)  crèche,  peutertuin, onderweg  school, (auto,  thuis werk hogeschool trein  universiteit bus, ...) vrije tijd andere |
| --- | --- | --- |
| (- )  (- )  (- )  (- )  (- )  (- )  (- )  (- )  (- )  (- )  (- )  (- )  (- )  (- )  (- ) |  |  |

**deze eerste dag (van 5u ’s morgens tot 5u ’s morgens de volgende dag)**

| Hoe dikwijls ziet u deze persoon?  (bijna) enkele enkele enkele  elke keren keren keren  dag per week per per jaar eerste  maand of minder keer | Hebt u hem/haar aangeraakt?  (bv hand geven, kus geven, sport)    ja neen | Tijd doorgebracht met deze persoon    Minder 5-15 15 min 1-4u 4u of  dan min -1u langer  5 min |
| --- | --- | --- |
|  |  |  |

Datum dag 2 / /

**Lijst van de personen met wie uw kind in contact is geweest tijdens**

| Leeftijd (of leeftijdscategorie) | Geslacht ♀ ♂  vrouw man | Plaats van contact (meerdere antwoorden mogelijk)  crèche,  peutertuin, onderweg  school, (auto,  thuis werk hogeschool trein  universiteit bus, ...) vrije tijd andere |
| --- | --- | --- |
| (- )  (- )  (- )  (- )  (- )  (- )  (- )  (- )  (- )  (- )  (- )  (- )  (- )  (- )  (- ) |  |  |

**deze tweede dag (van 5u ’s morgens tot 5u ’s morgens de volgende dag)**

| Hoe dikwijls ziet u deze persoon?  (bijna) enkele enkele enkele  elke keren keren keren  dag per week per per jaar eerste  maand of minder keer | Hebt u hem/haar aangeraakt?  (bv hand geven, kus geven, sport)    ja neen | Tijd doorgebracht met deze persoon    Minder 5-15 15 min 1-4u 4u of  dan min -1u langer  5 min |
| --- | --- | --- |
|  |  |  |

Datum dag 2 / /

**Lijst van de personen met wie uw kind in contact is geweest tijdens**

| Leeftijd (of leeftijdscategorie) | Geslacht ♀ ♂  vrouw man | Plaats van contact (meerdere antwoorden mogelijk)  crèche,  peutertuin, onderweg  school, (auto,  thuis werk hogeschool trein  universiteit bus, ...) vrije tijd andere |
| --- | --- | --- |
| (- )  (- )  (- )  (- )  (- )  (- )  (- )  (- )  (- )  (- )  (- )  (- )  (- )  (- )  (- ) |  |  |

**deze tweede dag (van 5u ’s morgens tot 5u ’s morgens de volgende dag)**

| Hoe dikwijls ziet u deze persoon?  (bijna) enkele enkele enkele  elke keren keren keren  dag per week per per jaar eerste  maand of minder keer | Hebt u hem/haar aangeraakt?  (bv hand geven, kus geven, sport)    ja neen | Tijd doorgebracht met deze persoon    Minder 5-15 15 min 1-4u 4u of  dan min -1u langer  5 min |
| --- | --- | --- |
|  |  |  |

Datum dag 2 / /

**Lijst van de personen met wie uw kind in contact is geweest tijdens**

| Leeftijd (of leeftijdscategorie) | Geslacht ♀ ♂  vrouw man | Plaats van contact (meerdere antwoorden mogelijk)  crèche,  peutertuin, onderweg  school, (auto,  thuis werk hogeschool trein  universiteit bus, ...) vrije tijd andere |
| --- | --- | --- |
| (- )  (- )  (- )  (- )  (- )  (- )  (- )  (- )  (- )  (- )  (- )  (- )  (- )  (- )  (- ) |  |  |

**deze tweede dag (van 5u ’s morgens tot 5u ’s morgens de volgende dag)**

| Hoe dikwijls ziet u deze persoon?  (bijna) enkele enkele enkele  elke keren keren keren  dag per week per per jaar eerste  maand of minder keer | Hebt u hem/haar aangeraakt?  (bv hand geven, kus geven, sport)    ja neen | Tijd doorgebracht met deze persoon    Minder 5-15 15 min 1-4u 4u of  dan min -1u langer  5 min |
| --- | --- | --- |
|  |  |  |

Datum dag 2 / /

**Lijst van de personen met wie uw kind in contact is geweest tijdens**

| Leeftijd (of leeftijdscategorie) | Geslacht ♀ ♂  vrouw man | Plaats van contact (meerdere antwoorden mogelijk)  crèche,  peutertuin, onderweg  school, (auto,  thuis werk hogeschool trein  universiteit bus, ...) vrije tijd andere |
| --- | --- | --- |
| (- )  (- )  (- )  (- )  (- )  (- )  (- )  (- )  (- )  (- )  (- )  (- )  (- )  (- )  (- ) |  |  |

**deze tweede dag (van 5u ’s morgens tot 5u ’s morgens de volgende dag)**

| Hoe dikwijls ziet u deze persoon?  (bijna) enkele enkele enkele  elke keren keren keren  dag per week per per jaar eerste  maand of minder keer | Hebt u hem/haar aangeraakt?  (bv hand geven, kus geven, sport)    ja neen | Tijd doorgebracht met deze persoon    Minder 5-15 15 min 1-4u 4u of  dan min -1u langer  5 min |
| --- | --- | --- |
|  |  |  |

Datum dag 2 / /

**Lijst van de personen met wie uw kind in contact is geweest tijdens**

| Leeftijd (of leeftijdscategorie) | Geslacht ♀ ♂  vrouw man | Plaats van contact (meerdere antwoorden mogelijk)  crèche,  peutertuin, onderweg  school, (auto,  thuis werk hogeschool trein  universiteit bus, ...) vrije tijd andere |
| --- | --- | --- |
| (- )  (- )  (- )  (- )  (- )  (- )  (- )  (- )  (- )  (- )  (- )  (- )  (- )  (- )  (- ) |  |  |

**deze tweede dag (van 5u ’s morgens tot 5u ’s morgens de volgende dag)**

| Hoe dikwijls ziet u deze persoon?  (bijna) enkele enkele enkele  elke keren keren keren  dag per week per per jaar eerste  maand of minder keer | Hebt u hem/haar aangeraakt?  (bv hand geven, kus geven, sport)    ja neen | Tijd doorgebracht met deze persoon    Minder 5-15 15 min 1-4u 4u of  dan min -1u langer  5 min |
| --- | --- | --- |
|  |  |  |

Datum dag 2 / /

**Lijst van de personen met wie uw kind in contact is geweest tijdens**

| Leeftijd (of leeftijdscategorie) | Geslacht ♀ ♂  vrouw man | Plaats van contact (meerdere antwoorden mogelijk)  crèche,  peutertuin, onderweg  school, (auto,  thuis werk hogeschool trein  universiteit bus, ...) vrije tijd andere |
| --- | --- | --- |
| (- )  (- )  (- )  (- )  (- )  (- )  (- )  (- )  (- )  (- )  (- )  (- )  (- )  (- )  (- ) |  |  |

**deze tweede dag (van 5u ’s morgens tot 5u ’s morgens de volgende dag)**

| Hoe dikwijls ziet u deze persoon?  (bijna) enkele enkele enkele  elke keren keren keren  dag per week per per jaar eerste  maand of minder keer | Hebt u hem/haar aangeraakt?  (bv hand geven, kus geven, sport)    ja neen | Tijd doorgebracht met deze persoon    Minder 5-15 15 min 1-4u 4u of  dan min -1u langer  5 min |
| --- | --- | --- |
|  |  |  |

15. Hebt u problemen gehad met het invullen van dit dagboekje? Indien ja, welke?

1. Hebt u het dagboekje bij de hand gehad en samen met uw kind om de paar uur ingevuld of enkel ’s avonds?

Dag 1

⬜ Tijdens de dag

⬜ ‘s avonds

⬜ andere, specifieer

Dag 2

⬜ tijdens de dag

⬜ ‘s avonds

⬜ andere, specifieer

1. Hoeveel contacten denkt u niet opgesomd te hebben, hetzij omdat uw kind ze vergeten is, hetzij omdat het er te veel waren.

Dag 1

⬜ 0

⬜ 1-4

⬜ 5-9

⬜ 10 of meer

Dag 2

⬜ 0

⬜ 1-4

⬜ 5-9

⬜ 10 of meer

Wij danken u nogmaals voor uw deelname.

Alle gegevens van dit dagboekje zullen confidentieel behandeld worden en zullen enkel gebruikt worden voor het wetenschappelijk onderzoek volgens de toepassing van de wet ter bescherming van de persoonlijke levenssfeer.


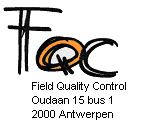


🖀 03-231 06 67

🖀 0800-93667
