# Supplementary material for: Mining social mixing patterns for infectious disease models based on a two-day population survey in Belgium
Source: BMC Infect Dis. 2009 Jan 20;9:5. doi: 10.1186/1471-2334-9-5 (PMC2656518; doi:10.1186/1471-2334-9-5)
Supplement: Additional file 2 — Diary Children French. original diaries in French for children. [file 1471-2334-9-5-S2.doc]

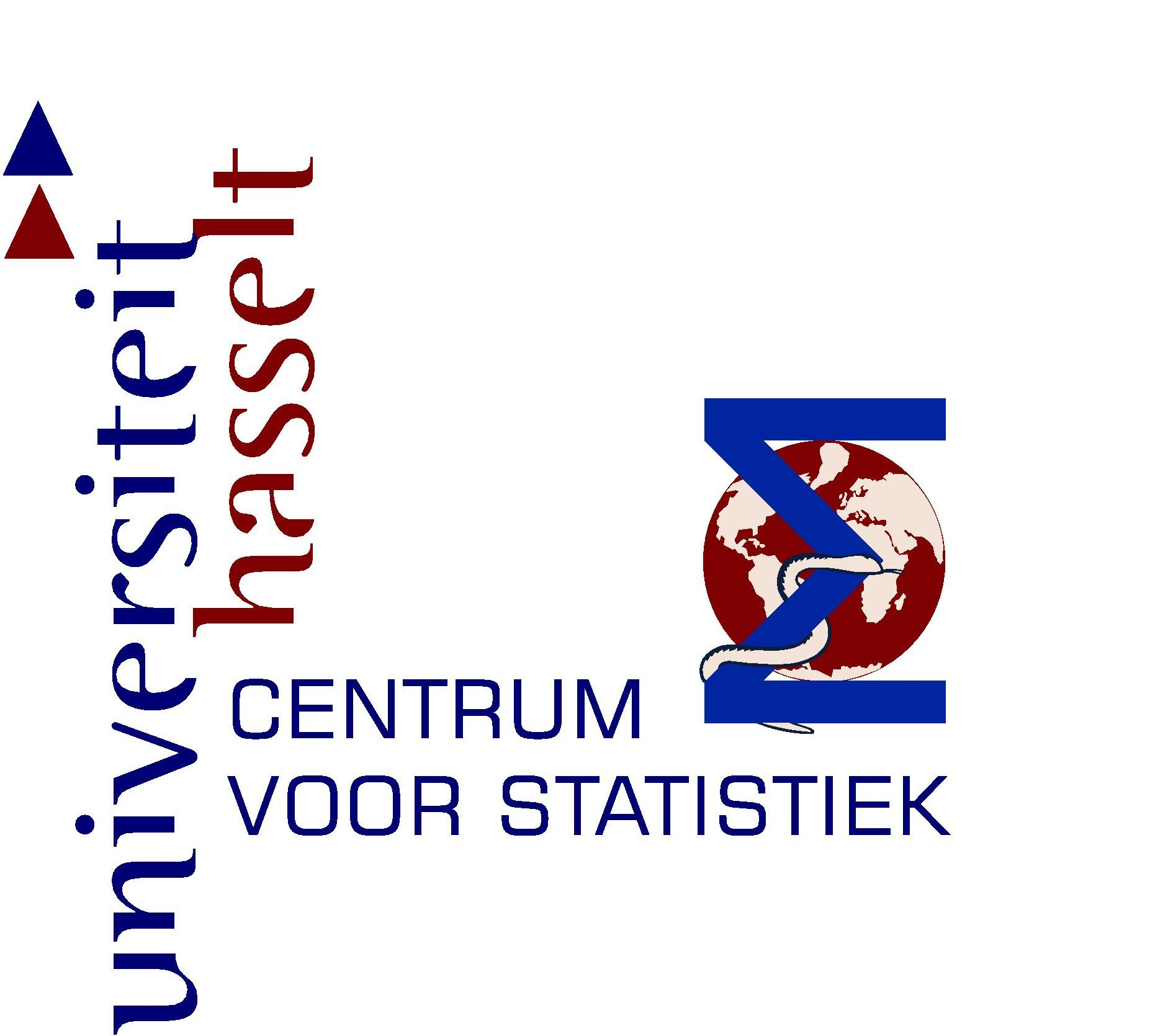


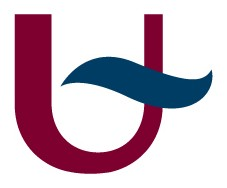


**Universiteit Antwerpen**


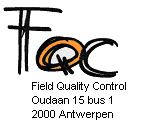


### Petit journal

### étude de contact

Si vous avez des problèmes ou des questions concernant le journal, n’hésitez pas à nous contacter au:

**03-231 06 67** ou **0800-93667**

Marie-Paule Feremans – Dave Van Ginkel

#### N°

##### Jour 1, date / /

Jour 2, date / /

**Comment remplir ce journal?**

- Nous vous prions d’indiquer dans ce journal toutes les personnes avec lesquelles votre enfant a été en contact direct et qu’il/elle a rencontrées au cours des deux journées que nous avons attribuées à votre enfant.
- Un contact veut dire que votre enfant a parlé avec quelqu’un en sa présence physique (conversations par téléphone ou GSM exclues). Un contact peut aussi être physique: toucher la peau d’une autre personne (se donner la main, donner une bise, des câlins ou en faisant du sport).
  - Les contacts avec des animaux ne comptent pas.
  - Important: il ne faut pas prendre en compte les personnes que votre enfant a uniquement contactées par téléphone ou GSM.
  - Si votre enfant ne connaît pas l’âge de la personne avec qui il/elle a été en contact, veuillez donner une estimation aussi précise que possible (p.ex. 40-45 ans).
  - Il faut seulement indiquer les contacts de votre enfant et non pas vos propres contacts. Nous vous conseillons de remplir le journal ensemble avec votre enfant pour être sûr de savoir qui il/elle a rencontré au cours de la journée.
- Veuillez utiliser une seule ligne par personne contactée: si votre enfant a contacté la même personne plusieurs fois dans la journée, ne l’indiquez qu’une seule fois et veuillez estimer le temps total que votre enfant a passé ensemble avec la personne lors de la journée attribuée.
- Le plus facile est d’essayer d’inscrire les contacts de votre enfant par ordre chronologique en commençant par la personne qu’il/elle a rencontrée en premier lors de la journée attribuée et puis de continuer avec toutes les autres personnes dont il/elle se souvient, en fonction de ses activités de la journée.
- Quand vous pensez avoir terminé la liste de contacts, nous vous demandons de bien réfléchir encore une fois (ensemble avec votre enfant) afin de vérifier que vous n’avez pas oublié une activité où votre enfant aurait pu avoir un contact. Un agenda d’école pourra être utile à cet effet.
- Pour les besoins de notre étude, la journée commence à 5 heures le matin de la journée attribuée et finit à 5 heures le lendemain matin.
- Dans les questions personnelles, on refère à ‘l’enfant’ comme l’enfant dans votre ménage sur qui ce petit journal porte.

Merci de fournir quelques données personnelles de vous-même (parent ou autre adulte):

1. Age ans
2. Sexe ⬜ féminin ⬜ masculin
3. Quel est votre lien avec l’enfant
   - Parent (beau-parent)
   - Autre famille (oncle, tante, grands-parents, …)
   - Autre
4. Situation professionnelle:
   - indépendant (artisan, commerçant, fermier, …)
   - cadre supérieur ou profession libérale (avocat, médecin, architecte, …)
   - employé(e)
   - ouvrier(ère)
   - retraité(e)
   - au foyer
   - étudiant(e)
   - à la recherche d’emploi
   - autre
5. Niveau d’éducation (quelles études avez-vous terminé):

⬜ Aucune étude

⬜ Enseignement primaire

⬜ Enseignement professionnel

⬜ Enseignement technique inférieur

⬜ Enseignement général inférieur

⬜ Enseignement technique secondaire

⬜ Enseignement général secondaire

⬜ Enseignement supérieur non universitaire

⬜ Enseignement universitaire

1. Nationalité:
   - belge
   - autre de l’Union européenne
   - autre en dehors de l’Union européenne
2. Le nombre de personnes dans votre ménage (sans l’enfant):
3. L’âge des membres de votre ménage (sans l’enfant), en commençant par le plus jeune: , , , , , , , , , , ,
4. Lieu de résidence 10. Code postal

Merci de fournir quelques données portant sur votre enfant:

11. Age ans

12. Sexe ⬜ féminin ⬜ masculin

13. Est-ce que votre enfant va dans une crèche/maternelle/primaire ou similaire?

⬜ oui habituellement il y a ⬜ < 10 enfants dans la crèche/maternelle…

⬜ 10-20 enfants dans la crèche/maternelle…

⬜ > 20 enfants dans la crèche/maternelle…

⬜ non

14. Nationalité

- - belge
  - autre de l’Union européenne

⬜ autre en dehors de l’Union européenne

# Exemple

| Age (ou fourchette) | Sexe ♀ ♂  féminin masculin | Lieu de contact (choix multiples possibles)  crèche,  maternelle, transport  école, (voitures,  à la lieu de lycée, train,  maison travail université bus, …) loisirs autres |
| --- | --- | --- |
| (- )  9  (- )  2  5  3  0 | X  X | X  X  X |

Première ligne: Le matin, votre enfant a parlé avec son frère de 9 ans en prenant le bus scolaire ensemble. Le soir ils ont joué ensemble entre 18-20 heures (avec contact physique).

Deuxième ligne: votre enfant a parlé avec une jeune vendeuse dans votre magasin de chaussures préféré, où vous allez plusieurs fois par an. Aujourd’hui votre enfant a essayé plusieurs pairs de chaussures.

| A quelle fréquence votre enfant rencontre cette personne  (presque) quelques quelques quelques 1ère  chaque fois par fois par fois par an fois  jour semaine mois ou moins  souvent | A-t-il (elle) touché sa peau?  (p.ex. se donner la main,  bises, sport)    oui non | Durée totale passée avec la personne      moins 5-15 15 min 1-4h 4h ou  de min -1h plus  5 min |
| --- | --- | --- |
| X  X | X  X | X  X |

Date jour 1 / /

**Liste de personnes avec lesquelles votre enfant a été en contact pendant**

| Age (ou fourchette) | Sexe ♀ ♂  féminin masculin | Lieu de contact (choix multiples possibles)  crèche, transport  maternelle, (voitures,  à la lieu de école, train, loisirs autres  maison travail lycée, bus, …)  université |
| --- | --- | --- |
| (- )  (- )  (- )  (- )  (- )  (- )  (- )  (- )  (- )  (- )  (- )  (- )  (- )  (- )  (- ) |  |  |

**la première journée attribuée entre 5 heures et 5 heures le lendemain matin**

| A quelle fréquence rencontrez-vous cette personne  (presque) quelques quelques quelques 1ère  chaque fois par fois par fois par an fois  jour semaine mois ou moins  souvent | Avez-vous touché sa peau?  (p.ex. se donner la main,  bises, sport)    oui non | Durée totale passée avec la personne  moins 5-15 15 min 1-4h 4h  de min - 1 h ou  5 min plus |
| --- | --- | --- |
|  |  |  |

Date jour 1 / /

**Liste de personnes avec lesquelles votre enfant a été en contact pendant**

| Age (ou fourchette) | Sexe ♀ ♂  féminin masculin | Lieu de contact (choix multiples possibles)  crèche, transport  maternelle, (voitures,  à la lieu de école, train, loisirs autres  maison travail lycée, bus, …)  université |
| --- | --- | --- |
| (- )  (- )  (- )  (- )  (- )  (- )  (- )  (- )  (- )  (- )  (- )  (- )  (- )  (- )  (- ) |  |  |

**la première journée attribuée entre 5 heures et 5 heures le lendemain matin**

| A quelle fréquence votre enfant rencontre cette personne  (presque) quelques quelques quelques 1ère  chaque fois par fois par fois par an fois  jour semaine mois ou moins  souvent | A-t-il (elle) touché sa peau?  (p.ex. se donner la main,  bises, sport)    oui non | Durée totale passée avec la personne  moins 5-15 15 min 1-4h 4h  de min - 1 h ou  5 min plus |
| --- | --- | --- |
|  |  |  |

Date jour 1 / /

**Liste de personnes avec lesquelles votre enfant a été en contact pendant**

| Age (ou fourchette) | Sexe ♀ ♂  féminin masculin | Lieu de contact (choix multiples possibles)  crèche, transport  maternelle, (voitures,  à la lieu de école, train, loisirs autres  maison travail lycée, bus, …)  université |
| --- | --- | --- |
| (- )  (- )  (- )  (- )  (- )  (- )  (- )  (- )  (- )  (- )  (- )  (- )  (- )  (- )  (- ) |  |  |

**la première journée attribuée entre 5 heures et 5 heures le lendemain matin**

| A quelle fréquence votre enfant rencontre cette personne  (presque) quelques quelques quelques 1ère  chaque fois par fois par fois par an fois  jour semaine mois ou moins  souvent | A-t-il (elle) touché sa peau?  (p.ex. se donner la main,  bises, sport)    oui non | Durée totale passée avec la personne  moins 5-15 15 min 1-4h 4h  de min - 1 h ou  5 min plus |
| --- | --- | --- |
|  |  |  |

Date jour 1 / /

**Liste de personnes avec lesquelles votre enfant a été en contact pendant**

| Age (ou fourchette) | Sexe ♀ ♂  féminin masculin | Lieu de contact (choix multiples possibles)  crèche, transport  maternelle, (voitures,  à la lieu de école, train, loisirs autres  maison travail lycée, bus, …)  université |
| --- | --- | --- |
| (- )  (- )  (- )  (- )  (- )  (- )  (- )  (- )  (- )  (- )  (- )  (- )  (- )  (- )  (- ) |  |  |

**la première journée attribuée entre 5 heures et 5 heures le lendemain matin**

| A quelle fréquence votre enfant rencontre cette personne  (presque) quelques quelques quelques 1ère  chaque fois par fois par fois par an fois  jour semaine mois ou moins  souvent | A-t-il (elle) touché sa peau?  (p.ex. se donner la main,  bises, sport)    oui non | Durée totale passée avec la personne  moins 5-15 15 min 1-4h 4h  de min - 1 h ou  5 min plus |
| --- | --- | --- |
|  |  |  |

Date jour 1 / /

Date jour 1 / /

**Liste de personnes avec lesquelles votre enfant a été en contact pendant**

| Age (ou fourchette) | Sexe ♀ ♂  féminin masculin | Lieu de contact (choix multiples possibles)  crèche, transport  maternelle, (voitures,  à la lieu de école, train, loisirs autres  maison travail lycée, bus, …)  université |
| --- | --- | --- |
| (- )  (- )  (- )  (- )  (- )  (- )  (- )  (- )  (- )  (- )  (- )  (- )  (- )  (- )  (- ) |  |  |

**la première journée attribuée entre 5 heures et 5 heures le lendemain matin**

| A quelle fréquence rencontrez-vous cette personne  (presque) quelques quelques quelques 1ère  chaque fois par fois par fois par an fois  jour semaine mois ou moins  souvent | Avez-vous touché sa peau?  (p.ex. se donner la main,  bises, sport)    oui non | Durée totale passée avec la personne  moins 5-15 15 min 1-4h 4h  de min - 1 h ou  5 min plus |
| --- | --- | --- |
|  |  |  |

Date jour 1 / /

**Liste de personnes avec lesquelles votre enfant a été en contact pendant**

| Age (ou fourchette) | Sexe ♀ ♂  féminin masculin | Lieu de contact (choix multiples possibles)  crèche, transport  maternelle, (voitures,  à la lieu de école, train, loisirs autres  maison travail lycée, bus, …)  université |
| --- | --- | --- |
| (- )  (- )  (- )  (- )  (- )  (- )  (- )  (- )  (- )  (- )  (- )  (- )  (- )  (- )  (- ) |  |  |

**la première journée attribuée entre 5 heures et 5 heures le lendemain matin**

| A quelle fréquence votre enfant rencontre cette personne  (presque) quelques quelques quelques 1ère  chaque fois par fois par fois par an fois  jour semaine mois ou moins  souvent | A-t-il (elle) touché sa peau?  (p.ex. se donner la main,  bises, sport)    oui non | Durée totale passée avec la personne  moins 5-15 15 min 1-4h 4h  de min - 1 h ou  5 min plus |
| --- | --- | --- |
|  |  |  |

Date jour 2 / /

**Liste de personnes avec lesquelles votre enfant a été en contact pendant**

| Age (ou fourchette) | Sexe ♀ ♂  féminin masculin | Lieu de contact (choix multiples possibles)  crèche, transport  maternelle, (voitures,  à la lieu de école, train, loisirs autres  maison travail lycée, bus, …)  université |
| --- | --- | --- |
| (- )  (- )  (- )  (- )  (- )  (- )  (- )  (- )  (- )  (- )  (- )  (- )  (- )  (- )  (- ) |  |  |

**la deuxième journée attribuée entre 5 heures et 5 heures le lendemain matin**

| A quelle fréquence votre enfant rencontre cette personne  (presque) quelques quelques quelques 1ère  chaque fois par fois par fois par an fois  jour semaine mois ou moins  souvent | A-t-il (elle) touché sa peau?  (p.ex. se donner la main,  bises, sport)    oui non | Durée totale passée avec la personne  moins 5-15 15 min 1-4h 4h  de min - 1 h ou  5 min plus |
| --- | --- | --- |
|  |  |  |

Date jour 2 / /

**Liste de personnes avec lesquelles votre enfant a été en contact pendant**

| Age (ou fourchette) | Sexe ♀ ♂  féminin masculin | Lieu de contact (choix multiples possibles)  crèche, transport  maternelle, (voitures,  à la lieu de école, train, loisirs autres  maison travail lycée, bus, …)  université |
| --- | --- | --- |
| (- )  (- )  (- )  (- )  (- )  (- )  (- )  (- )  (- )  (- )  (- )  (- )  (- )  (- )  (- ) |  |  |

**la deuxième journée attribuée entre 5 heures et 5 heures le lendemain matin**

| A quelle fréquence votre enfant rencontre cette personne  (presque) quelques quelques quelques 1ère  chaque fois par fois par fois par an fois  jour semaine mois ou moins  souvent | A-t-il (elle) touché sa peau?  (p.ex. se donner la main,  bises, sport)    oui non | Durée totale passée avec la personne  moins 5-15 15 min 1-4h 4h  de min - 1 h ou  5 min plus |
| --- | --- | --- |
|  |  |  |

Date jour 2 / /

**Liste de personnes avec lesquelles votre enfant a été en contact pendant**

| Age (ou fourchette) | Sexe ♀ ♂  féminin masculin | Lieu de contact (choix multiples possibles)  crèche, transport  maternelle, (voitures,  à la lieu de école, train, loisirs autres  maison travail lycée, bus, …)  université |
| --- | --- | --- |
| (- )  (- )  (- )  (- )  (- )  (- )  (- )  (- )  (- )  (- )  (- )  (- )  (- )  (- )  (- ) |  |  |

**la deuxième journée attribuée entre 5 heures et 5 heures le lendemain matin**

| A quelle fréquence votre enfant rencontre cette personne  (presque) quelques quelques quelques 1ère  chaque fois par fois par fois par an fois  jour semaine mois ou moins  souvent | A-t-il (elle) touché sa peau?  (p.ex. se donner la main,  bises, sport)    oui non | Durée totale passée avec la personne  moins 5-15 15 min 1-4h 4h  de min - 1 h ou  5 min plus |
| --- | --- | --- |
|  |  |  |

Date jour 2 / /

**Liste de personnes avec lesquelles votre enfant a été en contact pendant**

| Age (ou fourchette) | Sexe ♀ ♂  féminin masculin | Lieu de contact (choix multiples possibles)  crèche, transport  maternelle, (voitures,  à la lieu de école, train, loisirs autres  maison travail lycée, bus, …)  université |
| --- | --- | --- |
| (- )  (- )  (- )  (- )  (- )  (- )  (- )  (- )  (- )  (- )  (- )  (- )  (- )  (- )  (- ) |  |  |

**la deuxième journée attribuée entre 5 heures et 5 heures le lendemain matin**

| A quelle fréquence votre enfant rencontre cette personne  (presque) quelques quelques quelques 1ère  chaque fois par fois par fois par an fois  jour semaine mois ou moins  souvent | A-t-il (elle) touché sa peau?  (p.ex. se donner la main,  bises, sport)    oui non | Durée totale passée avec la personne  moins 5-15 15 min 1-4h 4h  de min - 1 h ou  5 min plus |
| --- | --- | --- |
|  |  |  |

Date jour 2 / /

**Liste de personnes avec lesquelles votre enfant a été en contact pendant**

| Age (ou fourchette) | Sexe ♀ ♂  féminin masculin | Lieu de contact (choix multiples possibles)  crèche, transport  maternelle, (voitures,  à la lieu de école, train, loisirs autres  maison travail lycée, bus, …)  université |
| --- | --- | --- |
| (- )  (- )  (- )  (- )  (- )  (- )  (- )  (- )  (- )  (- )  (- )  (- )  (- )  (- )  (- ) |  |  |

**la deuxième journée attribuée entre 5 heures et 5 heures le lendemain matin**

| A quelle fréquence votre enfant rencontre cette personne  (presque) quelques quelques quelques 1ère  chaque fois par fois par fois par an fois  jour semaine mois ou moins  souvent | A-t-il (elle) touché sa peau?  (p.ex. se donner la main,  bises, sport)    oui non | Durée totale passée avec la personne  moins 5-15 15 min 1-4h 4h  de min - 1 h ou  5 min plus |
| --- | --- | --- |
|  |  |  |

Date jour 2 / /

**Liste de personnes avec lesquelles votre enfant a été en contact pendant**

| Age (ou fourchette) | Sexe ♀ ♂  féminin masculin | Lieu de contact (choix multiples possibles)  crèche, transport  maternelle, (voitures,  à la lieu de école, train, loisirs autres  maison travail lycée, bus, …)  université |
| --- | --- | --- |
| (- )  (- )  (- )  (- )  (- )  (- )  (- )  (- )  (- )  (- )  (- )  (- )  (- )  (- )  (- ) |  |  |

**la deuxième journée attribuée entre 5 heures et 5 heures le lendemain matin**

| A quelle fréquence votre enfant rencontre cette personne  (presque) quelques quelques quelques 1ère  chaque fois par fois par fois par an fois  jour semaine mois ou moins  souvent | A-t-il (elle) touché sa peau?  (p.ex. se donner la main,  bises, sport)    oui non | Durée totale passée avec la personne  moins 5-15 15 min 1-4h 4h  de min - 1 h ou  5 min plus |
| --- | --- | --- |
|  |  |  |

15. Avez-vous eu des problèmes pour remplir ce journal? Si oui, merci de les préciser.

1. Avez-vous complété le journal avec votre enfant au fur et à mesure pendant les journées en question (en le gardant avec vous) ou uniquement le soir?

Journée 1

⬜ pendant la journée

⬜ le soir

⬜ autre, à spécifier

Journée 2

⬜ pendant la journée

⬜ le soir

⬜ autre, à spécifier

1. Combien de contacts pensez-vous ne pas avoir énumérés, soit parce que votre enfant les a oubliés ou parce qu’il y en avait trop?

Journée 1

⬜ 0

⬜ 1-4

⬜ 5-9

⬜ 10 ou plus

Journée 2

⬜ 0

⬜ 1-4

⬜ 5-9

⬜ 10 ou plus

Nous vous remercions encore une fois de votre participation.

Toutes les informations de ce journal seront traitées de façon confidentielle et ne seront utilisées qu’à des fins de recherche scientifique.


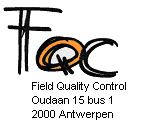


🖀 03-231 06 67

🖀 0800-93667
