# Supplementary material for: Mining social mixing patterns for infectious disease models based on a two-day population survey in Belgium
Source: BMC Infect Dis. 2009 Jan 20;9:5. doi: 10.1186/1471-2334-9-5 (PMC2656518; doi:10.1186/1471-2334-9-5)
Supplement: Additional file 3 — Diary Adolescents Dutch. original diaries in Dutch for adolescents. [file 1471-2334-9-5-S3.doc]

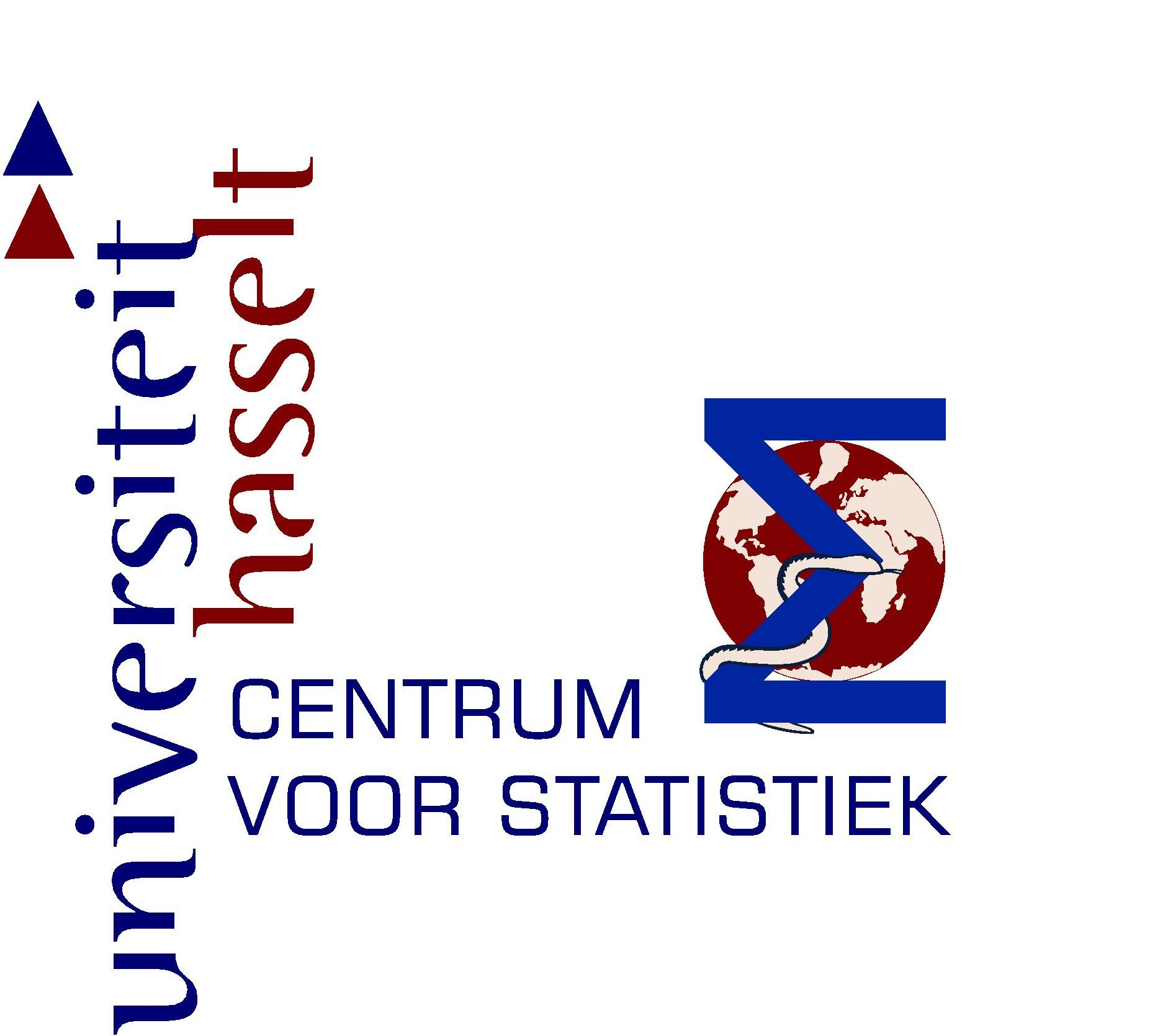


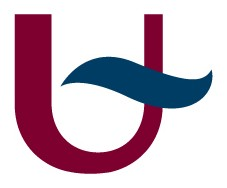


**Universiteit Antwerpen**


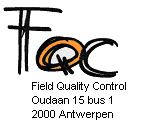


### Dagboekje

### studie contactpatronen

Indien u vragen hebt over het dagboekje, neem dan contact op met:

**03-231 06 67** of **0800-93667**

Marie-Paule Feremans – Dave Van Ginkel

#### Nr

##### Dag 1, datum / /

Dag 2, datum / /

**Instructies bij het invullen van het dagboekje**

- Gelieve alle personen met wie je direct contact hebt gehad en die je deze 2 dagen ontmoet hebt, te noteren in het dagboekje.
- Met ‘contact’ bedoelen we dat je met iemand gesproken hebt in zijn/haar aanwezigheid (geen telefonisch contact of contact via internet). Het contact kan ook fysiek zijn: iemand aanraken (hand geven, een kus geven, knuffelen, toevallige contacten tijdens sport).
  - Contact met dieren moet niet genoteerd worden.
  - Belangrijk: personen met wie je enkel telefonisch contact (GSM/internet) hebt gehad moeten niet opgenomen worden in het dagboekje.
  - Als je de exacte leeftijd van de persoon met wie je contact hebt gehad niet kent, geef dan een zo nauwkeurig mogelijke schatting (bv tussen 40 en 45 jaar).
  - Met ‘vrije tijd’ bedoelen we al wat je buiten de school doet, bv sport, muziekschool, met vrienden samen zijn, naar de film gaan, een activiteit uitoefenen in clubverband.
- Gebruik 1 regel per persoon met wie je contact had: als je met een bepaalde persoon verschillende keren contact had, schrijf dit dan eenmaal op en geef een schatting van de totale tijd die je samen hebt doorgebracht.
  - Het beste is dat je je dagboekje om de 1 à 2 uur even ter hand neemt en aanvult in functie van je contacten.
- Het meest eenvoudige is je contacten chronologisch in te vullen, te beginnen met het contact dat je het eerst had die dag en verdergaan met de andere personen die je je herinnert in functie van de activiteiten van die dag.
- Als je denkt klaar te zijn met de lijst van contacten die je gehad hebt die dag, overloop de dag dan nog eens grondig door na te kijken of je geen enkele activiteit vergeten bent waarbij je contact gehad hebt.
- Een ‘dag’ beschouwen we in deze studie als één die begint om 5u ‘s morgens en eindigt om 5u de volgende morgen.

Wij danken je voor het invullen van volgende persoonlijke gegevens:

1. Leeftijd jaar
2. Geslacht ⬜ vrouw ⬜ man
3. Wat is jouw situatie op deze moment?
   - Ik gaan naar school of ik volg een opleiding

Gemiddeld zijn we met personen in de klas

- - Ik werk
  - Ik zoek werk
  - andere

1. Nationaliteit:
   - Belg
   - Andere nationaliteit binnen de Europese Unie
   - Andere nationaliteit buiten de Europese Unie
2. Aantal personen die deel uitmaken van het gezin (jezelf niet meegerekend):
3. Leeftijd van de gezinsleden (jezelf niet meegerekend), te beginnen met de jongste: , , , , , , , , , , ,
4. Woonplaats 8. Postcode
5. Oefen je een beroep uit waarbij je veel contacten hebt? (cliënten, patiënten, studenten,.) ⬜ ja ⬜ neen

**Indien ja,** gelieve een schatting te maken van het gemiddeld aantal personen dat je per dag ziet (cliënten, patiënten, studenten, ....):

Deze professionele contacten situeren zich vooral in volgende leeftijdscategorieën: (meerdere antwoorden mogelijk):

⬜ 0-5 jaar ⬜ 6-11 jaar ⬜ 12-17 jaar ⬜ 18-60 jaar ⬜ ouder dan 60 jaar

Als je schat dat het aantal contacten meer dan 20 is, gelieve deze contacten dan niet allemaal op te sommen in je dagboekje maar enkel de andere (niet-professionele) contacten te vermelden

# Voorbeeld

| Leeftijd (of leeftijdscategorie) | Geslacht ♀ ♂  vrouw man | Plaats van contact (meerdere antwoorden mogelijk)  crèche,  peutertuin, onderweg  school, (auto,  thuis werk hogeschool trein  universiteit bus, ...) vrije tijd andere |
| --- | --- | --- |
| (- )  9  (- )  2  5  3  0 | X  X | X  X  X |

Eerste regel: je hebt ’s morgens 10 minuten met je broer van 9 jaar gepraat op de bus naar school. ’s Avonds heb je met hem gespeeld tussen 18h en 20h.

Tweede regel: Je hebt met een jonge verkoopster gesproken in je lievelingsschoenwinkel waar je een paar keer per jaar komt. Je hebt vandaag verschillende schoenen gepast.

| Hoe dikwijls ziet u deze persoon?  (bijna) enkele enkele enkele  elke keren keren keren  dag per week per per jaar eerste  maand of minder keer | Hebt u hem/haar aangeraakt?  (bv hand geven, kus geven, sport)    ja neen | Tijd doorgebracht met deze persoon      Minder 5-15 15 min 1-4u 4u of  dan min -1u langer  5 min |
| --- | --- | --- |
| X  X | X  X | X  X |

Datum dag 1 / /

**Lijst van de personen met wie u in contact bent geweest tijdens**

| Leeftijd (of leeftijdscategorie) | Geslacht ♀ ♂  vrouw man | Plaats van contact (meerdere antwoorden mogelijk)  crèche,  peutertuin, onderweg  school, (auto,  thuis werk hogeschool trein  universiteit bus, ...) vrije tijd andere |
| --- | --- | --- |
| (- )  (- )  (- )  (- )  (- )  (- )  (- )  (- )  (- )  (- )  (- )  (- )  (- )  (- )  (- ) |  |  |

**deze eerste dag (van 5u ’s morgens tot 5u ’s morgens de volgende dag)**

| Hoe dikwijls ziet u deze persoon?  (bijna) enkele enkele enkele  elke keren keren keren  dag per week per per jaar eerste  maand of minder keer | Hebt u hem/haar aangeraakt?  (bv hand geven, kus geven, sport)    ja neen | Tijd doorgebracht met deze persoon    Minder 5-15 15 min 1-4u 4u of  dan min -1u langer  5 min |
| --- | --- | --- |
|  |  |  |

Datum dag 1 / /

**Lijst van de personen met wie u in contact bent geweest tijdens**

| Leeftijd (of leeftijdscategorie) | Geslacht ♀ ♂  vrouw man | Plaats van contact (meerdere antwoorden mogelijk)  crèche,  peutertuin, onderweg  school, (auto,  thuis werk hogeschool trein  universiteit bus, ...) vrije tijd andere |
| --- | --- | --- |
| (- )  (- )  (- )  (- )  (- )  (- )  (- )  (- )  (- )  (- )  (- )  (- )  (- )  (- )  (- ) |  |  |

**deze eerste dag (van 5u ’s morgens tot 5u ’s morgens de volgende dag)**

| Hoe dikwijls ziet u deze persoon?  (bijna) enkele enkele enkele  elke keren keren keren  dag per week per per jaar eerste  maand of minder keer | Hebt u hem/haar aangeraakt?  (bv hand geven, kus geven, sport)    ja neen | Tijd doorgebracht met deze persoon    Minder 5-15 15 min 1-4u 4u of  dan min -1u langer  5 min |
| --- | --- | --- |
|  |  |  |

Datum dag 1 / /

**Lijst van de personen met wie u in contact bent geweest tijdens**

| Leeftijd (of leeftijdscategorie) | Geslacht ♀ ♂  vrouw man | Plaats van contact (meerdere antwoorden mogelijk)  crèche,  peutertuin, onderweg  school, (auto,  thuis werk hogeschool trein  universiteit bus, ...) vrije tijd andere |
| --- | --- | --- |
| (- )  (- )  (- )  (- )  (- )  (- )  (- )  (- )  (- )  (- )  (- )  (- )  (- )  (- )  (- ) |  |  |

**deze eerste dag (van 5u ’s morgens tot 5u ’s morgens de volgende dag)**

| Hoe dikwijls ziet u deze persoon?  (bijna) enkele enkele enkele  elke keren keren keren  dag per week per per jaar eerste  maand of minder keer | Hebt u hem/haar aangeraakt?  (bv hand geven, kus geven, sport)    ja neen | Tijd doorgebracht met deze persoon    Minder 5-15 15 min 1-4u 4u of  dan min -1u langer  5 min |
| --- | --- | --- |
|  |  |  |

Datum dag 1 / /

**Lijst van de personen met wie u in contact bent geweest tijdens**

| Leeftijd (of leeftijdscategorie) | Geslacht ♀ ♂  vrouw man | Plaats van contact (meerdere antwoorden mogelijk)  crèche,  peutertuin, onderweg  school, (auto,  thuis werk hogeschool trein  universiteit bus, ...) vrije tijd andere |
| --- | --- | --- |
| (- )  (- )  (- )  (- )  (- )  (- )  (- )  (- )  (- )  (- )  (- )  (- )  (- )  (- )  (- ) |  |  |

**deze eerste dag (van 5u ’s morgens tot 5u ’s morgens de volgende dag)**

| Hoe dikwijls ziet u deze persoon?  (bijna) enkele enkele enkele  elke keren keren keren  dag per week per per jaar eerste  maand of minder keer | Hebt u hem/haar aangeraakt?  (bv hand geven, kus geven, sport)    ja neen | Tijd doorgebracht met deze persoon    Minder 5-15 15 min 1-4u 4u of  dan min -1u langer  5 min |
| --- | --- | --- |
|  |  |  |

Datum dag 1 / /

**Lijst van de personen met wie u in contact bent geweest tijdens**

| Leeftijd (of leeftijdscategorie) | Geslacht ♀ ♂  vrouw man | Plaats van contact (meerdere antwoorden mogelijk)  crèche,  peutertuin, onderweg  school, (auto,  thuis werk hogeschool trein  universiteit bus, ...) vrije tijd andere |
| --- | --- | --- |
| (- )  (- )  (- )  (- )  (- )  (- )  (- )  (- )  (- )  (- )  (- )  (- )  (- )  (- )  (- ) |  |  |

**deze eerste dag (van 5u ’s morgens tot 5u ’s morgens de volgende dag)**

| Hoe dikwijls ziet u deze persoon?  (bijna) enkele enkele enkele  elke keren keren keren  dag per week per per jaar eerste  maand of minder keer | Hebt u hem/haar aangeraakt?  (bv hand geven, kus geven, sport)    ja neen | Tijd doorgebracht met deze persoon    Minder 5-15 15 min 1-4u 4u of  dan min -1u langer  5 min |
| --- | --- | --- |
|  |  |  |

Datum dag 1 / /

**Lijst van de personen met wie u in contact bent geweest tijdens**

| Leeftijd (of leeftijdscategorie) | Geslacht ♀ ♂  vrouw man | Plaats van contact (meerdere antwoorden mogelijk)  crèche,  peutertuin, onderweg  school, (auto,  thuis werk hogeschool trein  universiteit bus, ...) vrije tijd andere |
| --- | --- | --- |
| (- )  (- )  (- )  (- )  (- )  (- )  (- )  (- )  (- )  (- )  (- )  (- )  (- )  (- )  (- ) |  |  |

**deze eerste dag (van 5u ’s morgens tot 5u ’s morgens de volgende dag)**

| Hoe dikwijls ziet u deze persoon?  (bijna) enkele enkele enkele  elke keren keren keren  dag per week per per jaar eerste  maand of minder keer | Hebt u hem/haar aangeraakt?  (bv hand geven, kus geven, sport)    ja neen | Tijd doorgebracht met deze persoon    Minder 5-15 15 min 1-4u 4u of  dan min -1u langer  5 min |
| --- | --- | --- |
|  |  |  |

Datum dag 2 / /

**Lijst van de personen met wie u in contact bent geweest tijdens**

| Leeftijd (of leeftijdscategorie) | Geslacht ♀ ♂  vrouw man | Plaats van contact (meerdere antwoorden mogelijk)  crèche,  peutertuin, onderweg  school, (auto,  thuis werk hogeschool trein  universiteit bus, ...) vrije tijd andere |
| --- | --- | --- |
| (- )  (- )  (- )  (- )  (- )  (- )  (- )  (- )  (- )  (- )  (- )  (- )  (- )  (- )  (- ) |  |  |

**deze tweede dag (van 5u ’s morgens tot 5u ’s morgens de volgende dag)**

| Hoe dikwijls ziet u deze persoon?  (bijna) enkele enkele enkele  elke keren keren keren  dag per week per per jaar eerste  maand of minder keer | Hebt u hem/haar aangeraakt?  (bv hand geven, kus geven, sport)    ja neen | Tijd doorgebracht met deze persoon    Minder 5-15 15 min 1-4u 4u of  dan min -1u langer  5 min |
| --- | --- | --- |
|  |  |  |

Datum dag 2 / /

**Lijst van de personen met wie u in contact bent geweest tijdens**

| Leeftijd (of leeftijdscategorie) | Geslacht ♀ ♂  vrouw man | Plaats van contact (meerdere antwoorden mogelijk)  crèche,  peutertuin, onderweg  school, (auto,  thuis werk hogeschool trein  universiteit bus, ...) vrije tijd andere |
| --- | --- | --- |
| (- )  (- )  (- )  (- )  (- )  (- )  (- )  (- )  (- )  (- )  (- )  (- )  (- )  (- )  (- ) |  |  |

**deze tweede dag (van 5u ’s morgens tot 5u ’s morgens de volgende dag)**

| Hoe dikwijls ziet u deze persoon?  (bijna) enkele enkele enkele  elke keren keren keren  dag per week per per jaar eerste  maand of minder keer | Hebt u hem/haar aangeraakt?  (bv hand geven, kus geven, sport)    ja neen | Tijd doorgebracht met deze persoon    Minder 5-15 15 min 1-4u 4u of  dan min -1u langer  5 min |
| --- | --- | --- |
|  |  |  |

Datum dag 2 / /

**Lijst van de personen met wie u in contact bent geweest tijdens**

| Leeftijd (of leeftijdscategorie) | Geslacht ♀ ♂  vrouw man | Plaats van contact (meerdere antwoorden mogelijk)  crèche,  peutertuin, onderweg  school, (auto,  thuis werk hogeschool trein  universiteit bus, ...) vrije tijd andere |
| --- | --- | --- |
| (- )  (- )  (- )  (- )  (- )  (- )  (- )  (- )  (- )  (- )  (- )  (- )  (- )  (- )  (- ) |  |  |

**deze tweede dag (van 5u ’s morgens tot 5u ’s morgens de volgende dag)**

| Hoe dikwijls ziet u deze persoon?  (bijna) enkele enkele enkele  elke keren keren keren  dag per week per per jaar eerste  maand of minder keer | Hebt u hem/haar aangeraakt?  (bv hand geven, kus geven, sport)    ja neen | Tijd doorgebracht met deze persoon    Minder 5-15 15 min 1-4u 4u of  dan min -1u langer  5 min |
| --- | --- | --- |
|  |  |  |

Datum dag 2 / /

**Lijst van de personen met wie u in contact bent geweest tijdens**

| Leeftijd (of leeftijdscategorie) | Geslacht ♀ ♂  vrouw man | Plaats van contact (meerdere antwoorden mogelijk)  crèche,  peutertuin, onderweg  school, (auto,  thuis werk hogeschool trein  universiteit bus, ...) vrije tijd andere |
| --- | --- | --- |
| (- )  (- )  (- )  (- )  (- )  (- )  (- )  (- )  (- )  (- )  (- )  (- )  (- )  (- )  (- ) |  |  |

**deze tweede dag (van 5u ’s morgens tot 5u ’s morgens de volgende dag)**

| Hoe dikwijls ziet u deze persoon?  (bijna) enkele enkele enkele  elke keren keren keren  dag per week per per jaar eerste  maand of minder keer | Hebt u hem/haar aangeraakt?  (bv hand geven, kus geven, sport)    ja neen | Tijd doorgebracht met deze persoon    Minder 5-15 15 min 1-4u 4u of  dan min -1u langer  5 min |
| --- | --- | --- |
|  |  |  |

Datum dag 2 / /

**Lijst van de personen met wie u in contact bent geweest tijdens**

| Leeftijd (of leeftijdscategorie) | Geslacht ♀ ♂  vrouw man | Plaats van contact (meerdere antwoorden mogelijk)  crèche,  peutertuin, onderweg  school, (auto,  thuis werk hogeschool trein  universiteit bus, ...) vrije tijd andere |
| --- | --- | --- |
| (- )  (- )  (- )  (- )  (- )  (- )  (- )  (- )  (- )  (- )  (- )  (- )  (- )  (- )  (- ) |  |  |

**deze tweede dag (van 5u ’s morgens tot 5u ’s morgens de volgende dag)**

| Hoe dikwijls ziet u deze persoon?  (bijna) enkele enkele enkele  elke keren keren keren  dag per week per per jaar eerste  maand of minder keer | Hebt u hem/haar aangeraakt?  (bv hand geven, kus geven, sport)    ja neen | Tijd doorgebracht met deze persoon    Minder 5-15 15 min 1-4u 4u of  dan min -1u langer  5 min |
| --- | --- | --- |
|  |  |  |

Datum dag 2 / /

**Lijst van de personen met wie u in contact bent geweest tijdens**

| Leeftijd (of leeftijdscategorie) | Geslacht ♀ ♂  vrouw man | Plaats van contact (meerdere antwoorden mogelijk)  crèche,  peutertuin, onderweg  school, (auto,  thuis werk hogeschool trein  universiteit bus, ...) vrije tijd andere |
| --- | --- | --- |
| (- )  (- )  (- )  (- )  (- )  (- )  (- )  (- )  (- )  (- )  (- )  (- )  (- )  (- )  (- ) |  |  |

**deze tweede dag (van 5u ’s morgens tot 5u ’s morgens de volgende dag)**

| Hoe dikwijls ziet u deze persoon?  (bijna) enkele enkele enkele  elke keren keren keren  dag per week per per jaar eerste  maand of minder keer | Hebt u hem/haar aangeraakt?  (bv hand geven, kus geven, sport)    ja neen | Tijd doorgebracht met deze persoon    Minder 5-15 15 min 1-4u 4u of  dan min -1u langer  5 min |
| --- | --- | --- |
|  |  |  |

10. Heb je problemen gehad met het invullen van dit dagboekje? Indien ja, welke?

1. Heb je het dagboekje bij de hand gehad en om de paar uur ingevuld of enkel ’s avonds?

Dag 1

⬜ Tijdens de dag

⬜ ‘s avonds

⬜ andere, specifieer

Dag 2

⬜ tijdens de dag

⬜ ‘s avonds

⬜ andere, specifieer

1. Hoeveel contacten denk je niet opgesomd te hebben, hetzij omdat je ze vergeten bent, hetzij omdat het er te veel waren.

Dag 1

⬜ 0

⬜ 1-4

⬜ 5-9

⬜ 10 of meer

Dag 2

⬜ 0

⬜ 1-4

⬜ 5-9

⬜ 10 of meer

Wij danken je nogmaals voor je deelname.

Alle gegevens van dit dagboekje zullen confidentieel behandeld worden en zullen enkel gebruikt worden voor het wetenschappelijk onderzoek volgens de toepassing van de wet ter bescherming van de persoonlijke levenssfeer.


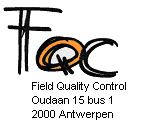


🖀 03-231 06 67

🖀 0800-93667
