# Supplementary material for: Mining social mixing patterns for infectious disease models based on a two-day population survey in Belgium
Source: BMC Infect Dis. 2009 Jan 20;9:5. doi: 10.1186/1471-2334-9-5 (PMC2656518; doi:10.1186/1471-2334-9-5)
Supplement: Additional file 4 — Diary Adolescents French. original diaries in French for adolescents. [file 1471-2334-9-5-S4.doc]

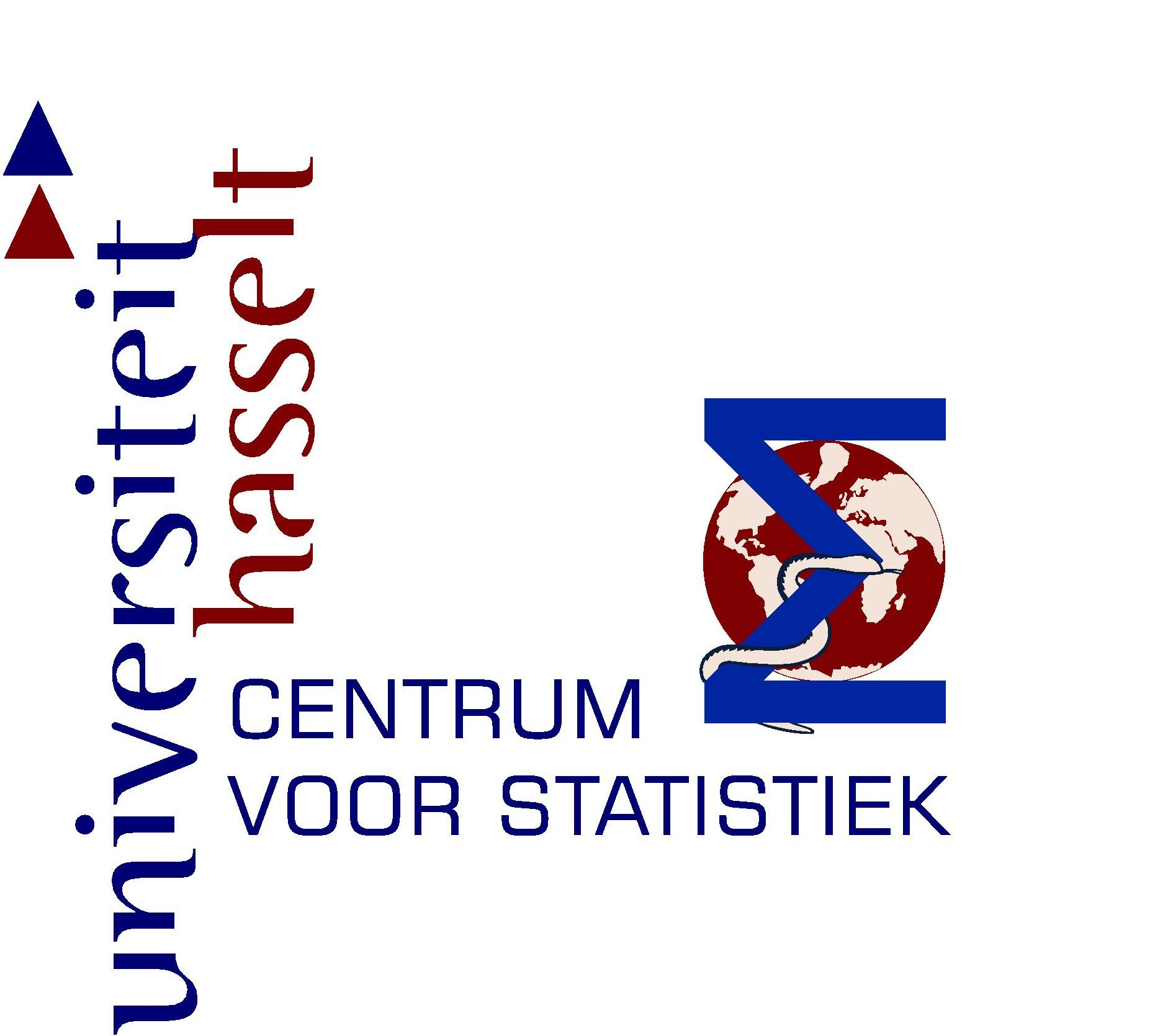


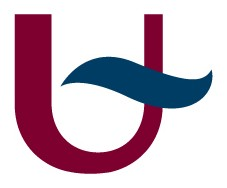


**Universiteit Antwerpen**


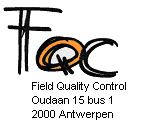


### Petit journal

### étude de contact

Si tu as des problèmes ou des questions concernant le journal, n’hésite pas à nous contacter au:

**03-231 06 67** ou **0800-93667**

Marie-Paule Feremans – Dave Van Ginkel

#### N°

##### Jour 1, date / /

Jour 2, date / /

**Comment remplir ce journal?**

- Nous te prions d’indiquer dans ce journal toutes les personnes que tu as rencontrées au cours des deux journées indiquées.
- Rencontrer veut dire que tu as parlé avec cette personne et que cette personne était à côté de toi. Donc les personnes avec lesquelles tu as seulement parlé au téléphone (ou GSM/internet) ne comptent pas comme contact. Un contact peut aussi être physique: toucher la peau de l’autre personne (se donner la main, donner une bise, des câlins ou en faisant du sport).
  - Les contacts avec des animaux ne comptent pas.
  - Important: il ne faut pas prendre en compte les personnes que tu as uniquement contactées par téléphone ou GSM.
  - Si tu ne connais pas l’âge exact de la personne contactée, essaie de deviner (p. ex. 40-45 ans) ou demande quelqu’un qui pourrait t’aider (parent, grand frère, grande soeur).
  - ‘Loisir’ correspond à toute activité que tu fais en dehors de l’école, par exemple faire du sport, de l’enseignement musical, voir des amis, aller au cinéma, faire des activités dans un club.
- Il faut utiliser une seule ligne par personne contactée: si tu as rencontré la même personne plusieurs fois dans la journée, ne l’indique qu’une seule fois et essaie de deviner au total combien de temps tu as passé avec elle pendant toute la journée.
- Le plus facile est d’essayer de commencer avec la personne que tu as rencontrée en premier le matin et de continuer en te rappelant ce que tu as fait pendant la journée.
- Quand tu as terminé la liste de tes contacts, nous te demandons de bien réfléchir encore une fois si tu n’as pas oublié une activité où tu aurais pu rencontrer quelqu’un.
- Il faudra seulement marquer tes contacts pendant la journée indiquée entre 5 heures le matin et 5 heures le lendemain matin.

Merci de fournir quelques données personnelles:

1. Age ans
2. Sexe ⬜ féminin ⬜ masculin
3. Quelle est ta situation en ce moment?
   - Je vais à l’école/lycée ou je suis une formation continue

Normalement je me trouve en classe avec autres personnes

- - J’ai du travail
  - Je cherche du travail
  - autre

1. Nationalité:
   - belge
   - autre de l’Union européenne
   - autre en dehors de l’Union européenne
2. Le nombre de personnes dans ton ménage (sans toi-même):
3. L’âge des membres de ton ménage (sans toi-même), en commençant par le plus jeune: , , , , , , , , , , ,
4. Lieu de résidence 8. Code postal
5. Si tu exerce une profession, est-ce que tu y rencontre beaucoup de personnes (clients, patients..)? ⬜ oui ⬜ non

**Si oui**, veux-tu estimer en moyenne le nombre de personnes (clients, patients, étudiants,...) que vous rencontrez par jour:

Ces contacts professionnels se situent plutôt dans les groupes suivantes (plusieurs possibilités): ⬜ 0-5 ans ⬜ 6-11 ans ⬜ 12-17 ans ⬜ 18-60 ans ⬜ plus de 60 ans

Si tu as estimé le nombre de ces contacts à plus de 20, nous te prions de ne pas énumérer ces contacts dans ton journal, et de seulement indiquer les autres contacts.

# Exemple

| Age (ou fourchette) | Sexe ♀ ♂  féminin masculin | Lieu de contact (choix multiples possibles)  crèche,  maternelle, transport  école, (voitures,  à la lieu de lycée, train,  maison travail université bus, …) loisirs autres |
| --- | --- | --- |
| (- )  9  (- )  2  5  3  0 | X  X | X  X  X |

Première ligne: tu parles pendant dix minutes avec ton frère de 9 ans dans le bus scolaire le matin. Le soir tu as joué ensemble entre 18-20 heures.

Deuxième ligne: tu as parlé avec une jeune vendeuse dans ton magasin de chaussures préféré, où tu vas plusieurs fois par an. Aujourd’hui tu as essayé plusieurs pairs de chaussures.

| A quelle fréquence rencontrez-vous cette personne  (presque) quelques quelques quelques 1ère  chaque fois par fois par fois par an fois  jour semaine mois ou moins  souvent | Avez-vous touché sa peau?  (p.ex. se donner la main,  bises, sport)    oui non | Durée totale passée avec la personne      moins 5-15 15 min 1-4h 4h ou  de min -1h plus  5 min |
| --- | --- | --- |
| X  X | X  X | X  X |

Date jour 1 / /

**Liste de personnes avec lesquelles tu as été en contact pendant la**

| Age (ou fourchette) | Sexe ♀ ♂  féminin masculin | Lieu de contact (choix multiples possibles)  crèche, transport  maternelle, (voitures,  à la lieu de école, train, loisirs autres  maison travail lycée, bus, …)  université |
| --- | --- | --- |
| (- )  (- )  (- )  (- )  (- )  (- )  (- )  (- )  (- )  (- )  (- )  (- )  (- )  (- )  (- ) |  |  |

**première journée attribuée entre 5 heures et 5 heures le lendemain matin**

| A quelle fréquence rencontrez-vous cette personne  (presque) quelques quelques quelques 1ère  chaque fois par fois par fois par an fois  jour semaine mois ou moins  souvent | Avez-vous touché sa peau?  (p.ex. se donner la main,  bises, sport)    oui non | Durée totale passée avec la personne  moins 5-15 15 min 1-4h 4h  de min - 1 h ou  5 min plus |
| --- | --- | --- |
|  |  |  |

Date jour 1 / /

**Liste de personnes avec lesquelles tu as été en contact pendant la**

| Age (ou fourchette) | Sexe ♀ ♂  féminin masculin | Lieu de contact (choix multiples possibles)  crèche, transport  maternelle, (voitures,  à la lieu de école, train, loisirs autres  maison travail lycée, bus, …)  université |
| --- | --- | --- |
| (- )  (- )  (- )  (- )  (- )  (- )  (- )  (- )  (- )  (- )  (- )  (- )  (- )  (- )  (- ) |  |  |

**première journée attribuée entre 5 heures et 5 heures le lendemain matin**

| A quelle fréquence rencontrez-vous cette personne  (presque) quelques quelques quelques 1ère  chaque fois par fois par fois par an fois  jour semaine mois ou moins  souvent | Avez-vous touché sa peau?  (p.ex. se donner la main,  bises, sport)    oui non | Durée totale passée avec la personne  moins 5-15 15 min 1-4h 4h  de min - 1 h ou  5 min plus |
| --- | --- | --- |
|  |  |  |

Date jour 1 / /

**Liste de personnes avec lesquelles tu as été en contact pendant la**

| Age (ou fourchette) | Sexe ♀ ♂  féminin masculin | Lieu de contact (choix multiples possibles)  crèche, transport  maternelle, (voitures,  à la lieu de école, train, loisirs autres  maison travail lycée, bus, …)  université |
| --- | --- | --- |
| (- )  (- )  (- )  (- )  (- )  (- )  (- )  (- )  (- )  (- )  (- )  (- )  (- )  (- )  (- ) |  |  |

**première journée attribuée entre 5 heures et 5 heures le lendemain matin**

| A quelle fréquence rencontrez-vous cette personne  (presque) quelques quelques quelques 1ère  chaque fois par fois par fois par an fois  jour semaine mois ou moins  souvent | Avez-vous touché sa peau?  (p.ex. se donner la main,  bises, sport)    oui non | Durée totale passée avec la personne  moins 5-15 15 min 1-4h 4h  de min - 1 h ou  5 min plus |
| --- | --- | --- |
|  |  |  |

Date jour 1 / /

**Liste de personnes avec lesquelles tu as été contact pendant la**

| Age (ou fourchette) | Sexe ♀ ♂  féminin masculin | Lieu de contact (choix multiples possibles)  crèche, transport  maternelle, (voitures,  à la lieu de école, train, loisirs autres  maison travail lycée, bus, …)  université |
| --- | --- | --- |
| (- )  (- )  (- )  (- )  (- )  (- )  (- )  (- )  (- )  (- )  (- )  (- )  (- )  (- )  (- ) |  |  |

**première journée attribuée entre 5 heures et 5 heures le lendemain matin**

| A quelle fréquence rencontrez-vous cette personne  (presque) quelques quelques quelques 1ère  chaque fois par fois par fois par an fois  jour semaine mois ou moins  souvent | Avez-vous touché sa peau?  (p.ex. se donner la main,  bises, sport)    oui non | Durée totale passée avec la personne  moins 5-15 15 min 1-4h 4h  de min - 1 h ou  5 min plus |
| --- | --- | --- |
|  |  |  |

Date jour 1 / /

**Liste de personnes avec lesquelles tu as été en contact pendant la**

| Age (ou fourchette) | Sexe ♀ ♂  féminin masculin | Lieu de contact (choix multiples possibles)  crèche, transport  maternelle, (voitures,  à la lieu de école, train, loisirs autres  maison travail lycée, bus, …)  université |
| --- | --- | --- |
| (- )  (- )  (- )  (- )  (- )  (- )  (- )  (- )  (- )  (- )  (- )  (- )  (- )  (- )  (- ) |  |  |

**première journée attribuée entre 5 heures et 5 heures le lendemain matin**

| A quelle fréquence rencontrez-vous cette personne  (presque) quelques quelques quelques 1ère  chaque fois par fois par fois par an fois  jour semaine mois ou moins  souvent | Avez-vous touché sa peau?  (p.ex. se donner la main,  bises, sport)    oui non | Durée totale passée avec la personne  moins 5-15 15 min 1-4h 4h  de min - 1 h ou  5 min plus |
| --- | --- | --- |
|  |  |  |

Date jour 1 / /

**Liste de personnes avec lesquelles tu as été en contact pendant la**

| Age (ou fourchette) | Sexe ♀ ♂  féminin masculin | Lieu de contact (choix multiples possibles)  crèche, transport  maternelle, (voitures,  à la lieu de école, train, loisirs autres  maison travail lycée, bus, …)  université |
| --- | --- | --- |
| (- )  (- )  (- )  (- )  (- )  (- )  (- )  (- )  (- )  (- )  (- )  (- )  (- )  (- )  (- ) |  |  |

**première journée attribuée entre 5 heures et 5 heures le lendemain matin**

| A quelle fréquence rencontrez-vous cette personne  (presque) quelques quelques quelques 1ère  chaque fois par fois par fois par an fois  jour semaine mois ou moins  souvent | Avez-vous touché sa peau?  (p.ex. se donner la main,  bises, sport)    oui non | Durée totale passée avec la personne  moins 5-15 15 min 1-4h 4h  de min - 1 h ou  5 min plus |
| --- | --- | --- |
|  |  |  |

Date jour 2 / /

**Liste de personnes avec lesquelles tu as été en contact pendant la**

| Age (ou fourchette) | Sexe ♀ ♂  féminin masculin | Lieu de contact (choix multiples possibles)  crèche, transport  maternelle, (voitures,  à la lieu de école, train, loisirs autres  maison travail lycée, bus, …)  université |
| --- | --- | --- |
| (- )  (- )  (- )  (- )  (- )  (- )  (- )  (- )  (- )  (- )  (- )  (- )  (- )  (- )  (- ) |  |  |

**deuxième journée attribuée entre 5 heures et 5 heures le lendemain matin**

| A quelle fréquence rencontrez-vous cette personne  (presque) quelques quelques quelques 1ère  chaque fois par fois par fois par an fois  jour semaine mois ou moins  souvent | Avez-vous touché sa peau?  (p.ex. se donner la main,  bises, sport)    oui non | Durée totale passée avec la personne  moins 5-15 15 min 1-4h 4h  de min - 1 h ou  5 min plus |
| --- | --- | --- |
|  |  |  |

Date jour 2 / /

**Liste de personnes avec lesquelles tu as été en contact pendant la**

| Age (ou fourchette) | Sexe ♀ ♂  féminin masculin | Lieu de contact (choix multiples possibles)  crèche, transport  maternelle, (voitures,  à la lieu de école, train, loisirs autres  maison travail lycée, bus, …)  université |
| --- | --- | --- |
| (- )  (- )  (- )  (- )  (- )  (- )  (- )  (- )  (- )  (- )  (- )  (- )  (- )  (- )  (- ) |  |  |

**deuxième journée attribuée entre 5 heures et 5 heures le lendemain matin**

| A quelle fréquence rencontrez-vous cette personne  (presque) quelques quelques quelques 1ère  chaque fois par fois par fois par an fois  jour semaine mois ou moins  souvent | Avez-vous touché sa peau?  (p.ex. se donner la main,  bises, sport)    oui non | Durée totale passée avec la personne  moins 5-15 15 min 1-4h 4h  de min - 1 h ou  5 min plus |
| --- | --- | --- |
|  |  |  |

Date jour 2 / /

**Liste de personnes avec lesquelles tu as été en contact pendant la**

| Age (ou fourchette) | Sexe ♀ ♂  féminin masculin | Lieu de contact (choix multiples possibles)  crèche, transport  maternelle, (voitures,  à la lieu de école, train, loisirs autres  maison travail lycée, bus, …)  université |
| --- | --- | --- |
| (- )  (- )  (- )  (- )  (- )  (- )  (- )  (- )  (- )  (- )  (- )  (- )  (- )  (- )  (- ) |  |  |

**deuxième journée attribuée entre 5 heures à 5 heures le lendemain matin**

| A quelle fréquence rencontrez-vous cette personne  (presque) quelques quelques quelques 1ère  chaque fois par fois par fois par an fois  jour semaine mois ou moins  souvent | Avez-vous touché sa peau?  (p.ex. se donner la main,  bises, sport)    oui non | Durée totale passée avec la personne  moins 5-15 15 min 1-4h 4h  de min - 1 h ou  5 min plus |
| --- | --- | --- |
|  |  |  |

Date jour 2 / /

**Liste de personnes avec lesquelles tu as été en contact pendant la**

| Age (ou fourchette) | Sexe ♀ ♂  féminin masculin | Lieu de contact (choix multiples possibles)  crèche, transport  maternelle, (voitures,  à la lieu de école, train, loisirs autres  maison travail lycée, bus, …)  université |
| --- | --- | --- |
| (- )  (- )  (- )  (- )  (- )  (- )  (- )  (- )  (- )  (- )  (- )  (- )  (- )  (- )  (- ) |  |  |

**deuxième journée attribuée entre 5 heures et 5 heures le lendemain matin**

| A quelle fréquence rencontrez-vous cette personne  (presque) quelques quelques quelques 1ère  chaque fois par fois par fois par an fois  jour semaine mois ou moins  souvent | Avez-vous touché sa peau?  (p.ex. se donner la main,  bises, sport)    oui non | Durée totale passée avec la personne  moins 5-15 15 min 1-4h 4h  de min - 1 h ou  5 min plus |
| --- | --- | --- |
|  |  |  |

Date jour 2 / /

**Liste de personnes avec lesquelles tu as été en contact pendant la**

| Age (ou fourchette) | Sexe ♀ ♂  féminin masculin | Lieu de contact (choix multiples possibles)  crèche, transport  maternelle, (voitures,  à la lieu de école, train, loisirs autres  maison travail lycée, bus, …)  université |
| --- | --- | --- |
| (- )  (- )  (- )  (- )  (- )  (- )  (- )  (- )  (- )  (- )  (- )  (- )  (- )  (- )  (- ) |  |  |

**deuxième journée attribuée entre 5 heures et 5 heures le lendemain matin**

| A quelle fréquence rencontrez-vous cette personne  (presque) quelques quelques quelques 1ère  chaque fois par fois par fois par an fois  jour semaine mois ou moins  souvent | Avez-vous touché sa peau?  (p.ex. se donner la main,  bises, sport)    oui non | Durée totale passée avec la personne  moins 5-15 15 min 1-4h 4h  de min - 1 h ou  5 min plus |
| --- | --- | --- |
|  |  |  |

Date jour 2 / /

**Liste de personnes avec lesquelles tu as été en contact pendant la**

| Age (ou fourchette) | Sexe ♀ ♂  féminin masculin | Lieu de contact (choix multiples possibles)  crèche, transport  maternelle, (voitures,  à la lieu de école, train, loisirs autres  maison travail lycée, bus, …)  université |
| --- | --- | --- |
| (- )  (- )  (- )  (- )  (- )  (- )  (- )  (- )  (- )  (- )  (- )  (- )  (- )  (- )  (- ) |  |  |

**deuxième journée attribuée entre 5 heures et 5 heures le lendemain matin**

| A quelle fréquence rencontrez-vous cette personne  (presque) quelques quelques quelques 1ère  chaque fois par fois par fois par an fois  jour semaine mois ou moins  souvent | Avez-vous touché sa peau?  (p.ex. se donner la main,  bises, sport)    oui non | Durée totale passée avec la personne  moins 5-15 15 min 1-4h 4h  de min - 1 h ou  5 min plus |
| --- | --- | --- |
|  |  |  |

1. As-tu eu des problèmes pour remplir ce journal? Si oui, merci de les préciser.
2. As-tu complété le journal au fur et à mesure pendant les journées en question (en le gardant avec toi) ou uniquement le soir?

Journée 1

⬜ pendant la journée

⬜ le soir

⬜ autre, à spécifier

Journée 2

⬜ pendant la journée

⬜ le soir

⬜ autre, à spécifier

1. Combien de contacts penses-tu ne pas avoir énumérés, soit parce que tu les a oubliés ou parce qu’il y en avait trop?

Journée 1

⬜ 0

⬜ 1-4

⬜ 5-9

⬜ 10 ou plus

Journée 2

⬜ 0

⬜ 1-4

⬜ 5-9

⬜ 10 ou plus

Nous te remercions encore une fois de ta participation.

Toutes les informations de ce journal seront traitées de façon confidentielle et ne seront utilisées qu’à des fins de recherche scientifique.


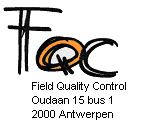


🖀 03-231 06 67

🖀 0800-93667
