# Supplementary material for: Mining social mixing patterns for infectious disease models based on a two-day population survey in Belgium
Source: BMC Infect Dis. 2009 Jan 20;9:5. doi: 10.1186/1471-2334-9-5 (PMC2656518; doi:10.1186/1471-2334-9-5)
Supplement: Additional file 5 — Diary Adults Dutch. original diaries in Dutch for adults. [file 1471-2334-9-5-S5.doc]

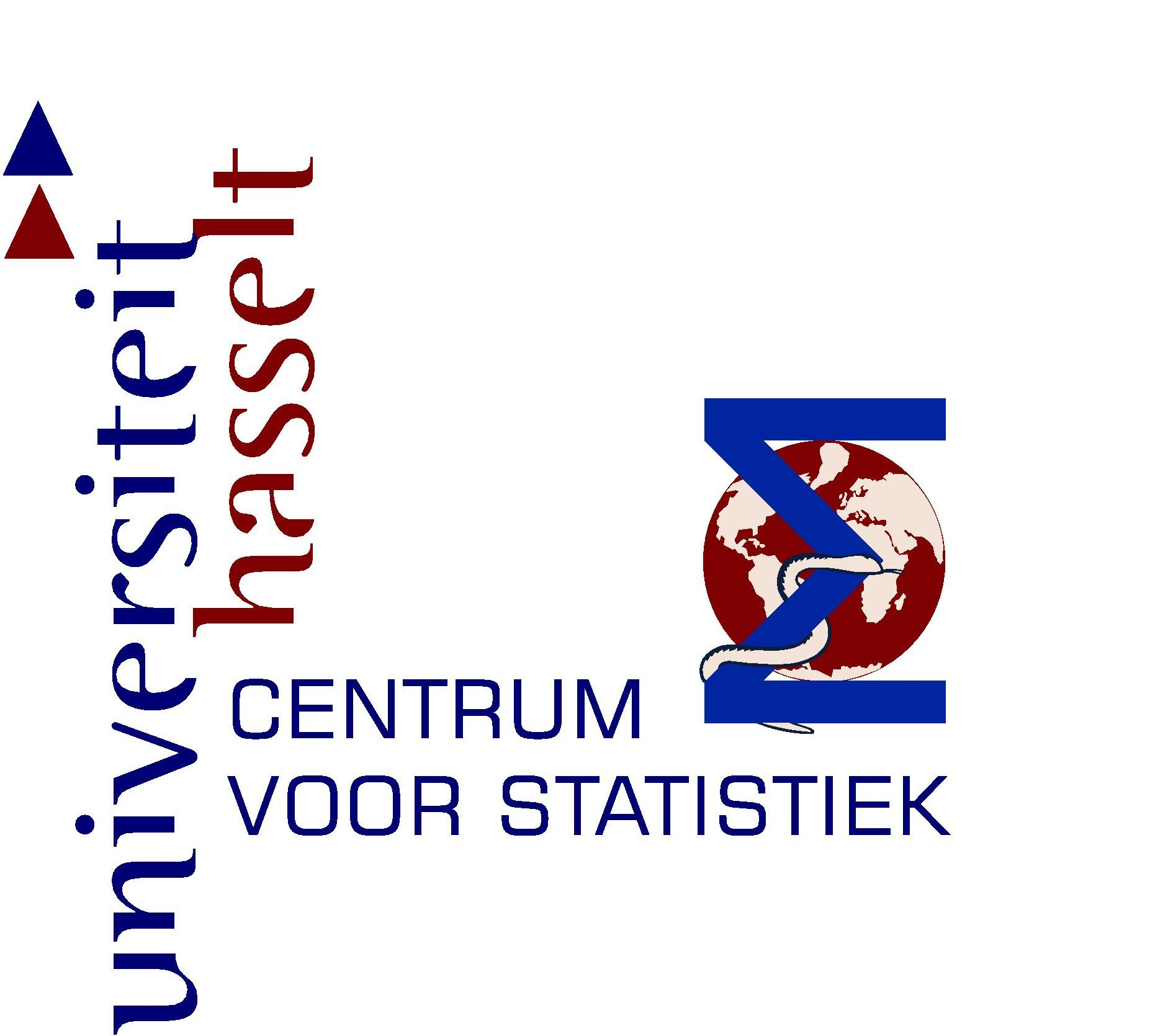


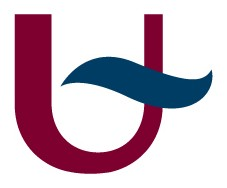


**Universiteit Antwerpen**


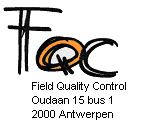


### Dagboekje

### studie contactpatronen

Indien u vragen hebt over het dagboekje, neem dan contact op met:

**03-231 06 67** of **0800-93667**

Marie-Paule Feremans – Dave Van Ginkel

#### Nr

##### Dag 1, datum / /

Dag 2, datum / /

**Instructies bij het invullen van het dagboekje**

- Gelieve alle personen met wie u direct contact hebt gehad en die u deze 2 dagen ontmoet hebt, te noteren in het dagboekje.
- Met ‘contact’ bedoelen we dat u met iemand gesproken heeft in zijn/haar aanwezigheid (geen telefonisch contact of contact via internet). Het contact kan ook fysiek zijn: iemand aanraken (hand geven, een kus geven, knuffelen, toevallige contacten tijdens sport).
  - Contact met dieren moet niet genoteerd worden.
  - Belangrijk: personen met wie u enkel telefonisch contact (GSM/internet) hebt gehad, moeten niet opgenomen worden in het dagboekje.
  - Als u de exacte leeftijd van de persoon met wie u contact hebt gehad niet kent, geef dan een zo nauwkeurig mogelijke schatting (bv tussen 40 en 45 jaar).
- Gebruik 1 regel per persoon met wie u contact had: als u met een bepaalde persoon verschillende keren contact had, schrijf dit dan eenmaal op en geef een schatting van de totale tijd die u samen hebt doorgebracht.
  - Het beste is dat u uw dagboekje om de 1 à 2 uur even ter hand neemt en aanvult in functie van uw contacten.
- Het meest eenvoudige is uw contacten chronologisch in te vullen, te beginnen met het contact dat u het eerst had die dag en verdergaan met de andere personen die u zich herinnert in functie van de activiteiten van die dag.
- Als u denkt klaar te zijn met de lijst van contacten die u gehad hebt die dag, overloop de dag dan nog eens grondig door na te kijken of u geen enkele activiteit vergeten bent waarbij u contact gehad hebt. Uw agenda kan hierbij een handig hulpmiddel zijn.
- Een ‘dag’ beschouwen we in deze studie als één die begint om 5h ‘s morgens en eindigt om 5h de volgende morgen.

Wij danken u voor het invullen van volgende persoonlijke gegevens:

1. Leeftijd jaar
2. Geslacht ⬜ vrouw ⬜ man
3. Beroep
   - Zelfstandige (kunstenaar, handelaar, landbouwer, …)
   - Kader of vrij beroep (advocaat, dokter, architect, …)
   - bediende
   - arbeider
   - gepensioneerde
   - huisvrouw/man
   - student(e)
   - werkzoekend
   - andere
4. Opleidingsniveau (welke studies hebt u beëindigd):

⬜ geen enkele

⬜ basisonderwijs (lagere school)

⬜ beroepsonderwijs

⬜ lager technisch onderwijs

⬜ lager secundair onderwijs

⬜ hoger technisch onderwijs

⬜ hoger secundair onderwijs

⬜ hoger onderwijs buiten de universiteit

⬜ universitair onderwijs

1. Nationaliteit:
   - Belg
   - Andere nationaliteit binnen de Europese Unie
   - Andere nationaliteit buiten de Europese Unie
2. Aantal personen die deel uitmaken van het gezin (uzelf niet meegerekend):
3. Leeftijd van de gezinsleden (uzelf niet meegerekend), te beginnen met de jongste: , , , , , , , , , , ,
4. Woonplaats 9. Postcode

10. Oefent u een beroep uit waarbij u veel contacten hebt? (cliënten, patiënten, studenten,.) ⬜ ja ⬜ neen

**Indien ja,** gelieve een schatting te maken van het gemiddeld aantal personen dat u per dag ziet (cliënten, patiënten, studenten, ....):

Deze professionele contacten situeren zich vooral in volgende leeftijdscategorieën (meerdere antwoorden mogelijk):

⬜ 0-5 jaar ⬜ 6-11 jaar ⬜ 12-17 jaar ⬜ 18-60 jaar ⬜ ouder dan 60 jaar

Als u schat dat het aantal contacten meer dan 20 is, gelieve deze contacten dan niet allemaal op te sommen in uw dagboekje maar enkel de andere (niet-professionele) contacten te vermelden.

# Voorbeeld

| Leeftijd (of leeftijdscategorie) | Geslacht ♀ ♂  vrouw man | Plaats van contact (meerdere antwoorden mogelijk)  crèche,  peutertuin, onderweg  school, (auto,  thuis werk hogeschool trein  universiteit bus, ...) vrije tijd andere |
| --- | --- | --- |
| (- )  9  (- )  2  5  3  0 | X  X | X  X  X |

Eerste regel: u hebt ’s morgens 10 minuten met uw zoon van 9 jaar gepraat terwijl u hem naar school bracht. ’s Avonds hebt u samen gespeeld tussen 18h en 20h en u hebt hem een kus gegeven voor hij naar bed ging.

Tweede regel: U hebt met een jonge verkoopster gesproken in uw lievelingsschoenwinkel waar u een paar keer per jaar komt. U hebt vandaag verschillende schoenen gepast.

| Hoe dikwijls ziet u deze persoon?  (bijna) enkele enkele enkele  elke keren keren keren  dag per week per per jaar eerste  maand of minder keer | Hebt u hem/haar aangeraakt?  (bv hand geven, kus geven, sport)    ja neen | Tijd doorgebracht met deze persoon      Minder 5-15 15 min 1-4u 4u of  dan min -1u langer  5 min |
| --- | --- | --- |
| X  X | X  X | X  X |

Datum dag 1 / /

**Lijst van de personen met wie u in contact bent geweest tijdens**

| Leeftijd (of leeftijdscategorie) | Geslacht ♀ ♂  vrouw man | Plaats van contact (meerdere antwoorden mogelijk)  crèche,  peutertuin, onderweg  school, (auto,  thuis werk hogeschool trein  universiteit bus, ...) vrije tijd andere |
| --- | --- | --- |
| (- )  (- )  (- )  (- )  (- )  (- )  (- )  (- )  (- )  (- )  (- )  (- )  (- )  (- )  (- ) |  |  |

**deze eerste dag (van 5u ’s morgens tot 5u ’s morgens de volgende dag)**

| Hoe dikwijls ziet u deze persoon?  (bijna) enkele enkele enkele  elke keren keren keren  dag per week per per jaar eerste  maand of minder keer | Hebt u hem/haar aangeraakt?  (bv hand geven, kus geven, sport)    ja neen | Tijd doorgebracht met deze persoon    Minder 5-15 15 min 1-4u 4u of  dan min -1u langer  5 min |
| --- | --- | --- |
|  |  |  |

Datum dag 1 / /

**Lijst van de personen met wie u in contact bent geweest tijdens**

| Leeftijd (of leeftijdscategorie) | Geslacht ♀ ♂  vrouw man | Plaats van contact (meerdere antwoorden mogelijk)  crèche,  peutertuin, onderweg  school, (auto,  thuis werk hogeschool trein  universiteit bus, ...) vrije tijd andere |
| --- | --- | --- |
| (- )  (- )  (- )  (- )  (- )  (- )  (- )  (- )  (- )  (- )  (- )  (- )  (- )  (- )  (- ) |  |  |

**deze eerste dag (van 5u ’s morgens tot 5u ’s morgens de volgende dag)**

| Hoe dikwijls ziet u deze persoon?  (bijna) enkele enkele enkele  elke keren keren keren  dag per week per per jaar eerste  maand of minder keer | Hebt u hem/haar aangeraakt?  (bv hand geven, kus geven, sport)    ja neen | Tijd doorgebracht met deze persoon    Minder 5-15 15 min 1-4u 4u of  dan min -1u langer  5 min |
| --- | --- | --- |
|  |  |  |

Datum dag 1 / /

**Lijst van de personen met wie u in contact bent geweest tijdens**

| Leeftijd (of leeftijdscategorie) | Geslacht ♀ ♂  vrouw man | Plaats van contact (meerdere antwoorden mogelijk)  crèche,  peutertuin, onderweg  school, (auto,  thuis werk hogeschool trein  universiteit bus, ...) vrije tijd andere |
| --- | --- | --- |
| (- )  (- )  (- )  (- )  (- )  (- )  (- )  (- )  (- )  (- )  (- )  (- )  (- )  (- )  (- ) |  |  |

**deze eerste dag (van 5u ’s morgens tot 5u ’s morgens de volgende dag)**

| Hoe dikwijls ziet u deze persoon?  (bijna) enkele enkele enkele  elke keren keren keren  dag per week per per jaar eerste  maand of minder keer | Hebt u hem/haar aangeraakt?  (bv hand geven, kus geven, sport)    ja neen | Tijd doorgebracht met deze persoon    Minder 5-15 15 min 1-4u 4u of  dan min -1u langer  5 min |
| --- | --- | --- |
|  |  |  |

Datum dag 1 / /

**Lijst van de personen met wie u in contact bent geweest tijdens**

| Leeftijd (of leeftijdscategorie) | Geslacht ♀ ♂  vrouw man | Plaats van contact (meerdere antwoorden mogelijk)  crèche,  peutertuin, onderweg  school, (auto,  thuis werk hogeschool trein  universiteit bus, ...) vrije tijd andere |
| --- | --- | --- |
| (- )  (- )  (- )  (- )  (- )  (- )  (- )  (- )  (- )  (- )  (- )  (- )  (- )  (- )  (- ) |  |  |

**deze eerste dag (van 5u ’s morgens tot 5u ’s morgens de volgende dag)**

| Hoe dikwijls ziet u deze persoon?  (bijna) enkele enkele enkele  elke keren keren keren  dag per week per per jaar eerste  maand of minder keer | Hebt u hem/haar aangeraakt?  (bv hand geven, kus geven, sport)    ja neen | Tijd doorgebracht met deze persoon    Minder 5-15 15 min 1-4u 4u of  dan min -1u langer  5 min |
| --- | --- | --- |
|  |  |  |

Datum dag 1 / /

**Lijst van de personen met wie u in contact bent geweest tijdens**

| Leeftijd (of leeftijdscategorie) | Geslacht ♀ ♂  vrouw man | Plaats van contact (meerdere antwoorden mogelijk)  crèche,  peutertuin, onderweg  school, (auto,  thuis werk hogeschool trein  universiteit bus, ...) vrije tijd andere |
| --- | --- | --- |
| (- )  (- )  (- )  (- )  (- )  (- )  (- )  (- )  (- )  (- )  (- )  (- )  (- )  (- )  (- ) |  |  |

**deze eerste dag (van 5u ’s morgens tot 5u ’s morgens de volgende dag)**

| Hoe dikwijls ziet u deze persoon?  (bijna) enkele enkele enkele  elke keren keren keren  dag per week per per jaar eerste  maand of minder keer | Hebt u hem/haar aangeraakt?  (bv hand geven, kus geven, sport)    ja neen | Tijd doorgebracht met deze persoon    Minder 5-15 15 min 1-4u 4u of  dan min -1u langer  5 min |
| --- | --- | --- |
|  |  |  |

Datum dag 1 / /

**Lijst van de personen met wie u in contact bent geweest tijdens**

| Leeftijd (of leeftijdscategorie) | Geslacht ♀ ♂  vrouw man | Plaats van contact (meerdere antwoorden mogelijk)  crèche,  peutertuin, onderweg  school, (auto,  thuis werk hogeschool trein  universiteit bus, ...) vrije tijd andere |
| --- | --- | --- |
| (- )  (- )  (- )  (- )  (- )  (- )  (- )  (- )  (- )  (- )  (- )  (- )  (- )  (- )  (- ) |  |  |

**deze eerste dag (van 5u ’s morgens tot 5u ’s morgens de volgende dag)**

| Hoe dikwijls ziet u deze persoon?  (bijna) enkele enkele enkele  elke keren keren keren  dag per week per per jaar eerste  maand of minder keer | Hebt u hem/haar aangeraakt?  (bv hand geven, kus geven, sport)    ja neen | Tijd doorgebracht met deze persoon    Minder 5-15 15 min 1-4u 4u of  dan min -1u langer  5 min |
| --- | --- | --- |
|  |  |  |

Datum dag 2 / /

**Lijst van de personen met wie u in contact bent geweest tijdens**

| Leeftijd (of leeftijdscategorie) | Geslacht ♀ ♂  vrouw man | Plaats van contact (meerdere antwoorden mogelijk)  crèche,  peutertuin, onderweg  school, (auto,  thuis werk hogeschool trein  universiteit bus, ...) vrije tijd andere |
| --- | --- | --- |
| (- )  (- )  (- )  (- )  (- )  (- )  (- )  (- )  (- )  (- )  (- )  (- )  (- )  (- )  (- ) |  |  |

**deze tweede dag (van 5u ’s morgens tot 5u ’s morgens de volgende dag)**

| Hoe dikwijls ziet u deze persoon?  (bijna) enkele enkele enkele  elke keren keren keren  dag per week per per jaar eerste  maand of minder keer | Hebt u hem/haar aangeraakt?  (bv hand geven, kus geven, sport)    ja neen | Tijd doorgebracht met deze persoon    Minder 5-15 15 min 1-4u 4u of  dan min -1u langer  5 min |
| --- | --- | --- |
|  |  |  |

Datum dag 2 / /

**Lijst van de personen met wie u in contact bent geweest tijdens**

| Leeftijd (of leeftijdscategorie) | Geslacht ♀ ♂  vrouw man | Plaats van contact (meerdere antwoorden mogelijk)  crèche,  peutertuin, onderweg  school, (auto,  thuis werk hogeschool trein  universiteit bus, ...) vrije tijd andere |
| --- | --- | --- |
| (- )  (- )  (- )  (- )  (- )  (- )  (- )  (- )  (- )  (- )  (- )  (- )  (- )  (- )  (- ) |  |  |

**deze tweede dag (van 5u ’s morgens tot 5u ’s morgens de volgende dag)**

| Hoe dikwijls ziet u deze persoon?  (bijna) enkele enkele enkele  elke keren keren keren  dag per week per per jaar eerste  maand of minder keer | Hebt u hem/haar aangeraakt?  (bv hand geven, kus geven, sport)    ja neen | Tijd doorgebracht met deze persoon    Minder 5-15 15 min 1-4u 4u of  dan min -1u langer  5 min |
| --- | --- | --- |
|  |  |  |

Datum dag 2 / /

**Lijst van de personen met wie u in contact bent geweest tijdens**

| Leeftijd (of leeftijdscategorie) | Geslacht ♀ ♂  vrouw man | Plaats van contact (meerdere antwoorden mogelijk)  crèche,  peutertuin, onderweg  school, (auto,  thuis werk hogeschool trein  universiteit bus, ...) vrije tijd andere |
| --- | --- | --- |
| (- )  (- )  (- )  (- )  (- )  (- )  (- )  (- )  (- )  (- )  (- )  (- )  (- )  (- )  (- ) |  |  |

**deze tweede dag (van 5u ’s morgens tot 5u ’s morgens de volgende dag)**

| Hoe dikwijls ziet u deze persoon?  (bijna) enkele enkele enkele  elke keren keren keren  dag per week per per jaar eerste  maand of minder keer | Hebt u hem/haar aangeraakt?  (bv hand geven, kus geven, sport)    ja neen | Tijd doorgebracht met deze persoon    Minder 5-15 15 min 1-4u 4u of  dan min -1u langer  5 min |
| --- | --- | --- |
|  |  |  |

Datum dag 2 / /

**Lijst van de personen met wie u in contact bent geweest tijdens**

| Leeftijd (of leeftijdscategorie) | Geslacht ♀ ♂  vrouw man | Plaats van contact (meerdere antwoorden mogelijk)  crèche,  peutertuin, onderweg  school, (auto,  thuis werk hogeschool trein  universiteit bus, ...) vrije tijd andere |
| --- | --- | --- |
| (- )  (- )  (- )  (- )  (- )  (- )  (- )  (- )  (- )  (- )  (- )  (- )  (- )  (- )  (- ) |  |  |

**deze tweede dag (van 5u ’s morgens tot 5u ’s morgens de volgende dag)**

| Hoe dikwijls ziet u deze persoon?  (bijna) enkele enkele enkele  elke keren keren keren  dag per week per per jaar eerste  maand of minder keer | Hebt u hem/haar aangeraakt?  (bv hand geven, kus geven, sport)    ja neen | Tijd doorgebracht met deze persoon    Minder 5-15 15 min 1-4u 4u of  dan min -1u langer  5 min |
| --- | --- | --- |
|  |  |  |

Datum dag 2 / /

**Lijst van de personen met wie u in contact bent geweest tijdens**

| Leeftijd (of leeftijdscategorie) | Geslacht ♀ ♂  vrouw man | Plaats van contact (meerdere antwoorden mogelijk)  crèche,  peutertuin, onderweg  school, (auto,  thuis werk hogeschool trein  universiteit bus, ...) vrije tijd andere |
| --- | --- | --- |
| (- )  (- )  (- )  (- )  (- )  (- )  (- )  (- )  (- )  (- )  (- )  (- )  (- )  (- )  (- ) |  |  |

**deze tweede dag (van 5u ’s morgens tot 5u ’s morgens de volgende dag)**

| Hoe dikwijls ziet u deze persoon?  (bijna) enkele enkele enkele  elke keren keren keren  dag per week per per jaar eerste  maand of minder keer | Hebt u hem/haar aangeraakt?  (bv hand geven, kus geven, sport)    ja neen | Tijd doorgebracht met deze persoon    Minder 5-15 15 min 1-4u 4u of  dan min -1u langer  5 min |
| --- | --- | --- |
|  |  |  |

Datum dag 2 / /

**Lijst van de personen met wie u in contact bent geweest tijdens**

| Leeftijd (of leeftijdscategorie) | Geslacht ♀ ♂  vrouw man | Plaats van contact (meerdere antwoorden mogelijk)  crèche,  peutertuin, onderweg  school, (auto,  thuis werk hogeschool trein  universiteit bus, ...) vrije tijd andere |
| --- | --- | --- |
| (- )  (- )  (- )  (- )  (- )  (- )  (- )  (- )  (- )  (- )  (- )  (- )  (- )  (- )  (- ) |  |  |

**deze tweede dag (van 5u ’s morgens tot 5u ’s morgens de volgende dag)**

| Hoe dikwijls ziet u deze persoon?  (bijna) enkele enkele enkele  elke keren keren keren  dag per week per per jaar eerste  maand of minder keer | Hebt u hem/haar aangeraakt?  (bv hand geven, kus geven, sport)    ja neen | Tijd doorgebracht met deze persoon    Minder 5-15 15 min 1-4u 4u of  dan min -1u langer  5 min |
| --- | --- | --- |
|  |  |  |

11. Hebt u problemen gehad met het invullen van dit dagboekje? Indien ja, welke?

12. Hebt u het dagboekje bij de hand gehad en om de paar uur ingevuld of enkel ’s

avonds?

Dag 1

- - Tijdens de dag
  - ‘s avonds
  - andere, specifieer

Dag 2

- - tijdens de dag
  - ‘s avonds
  - andere, specifieer

1. Hoeveel contacten denkt u niet opgesomd te hebben, hetzij omdat u ze vergeten bent, hetzij omdat het er te veel waren.

Dag 1

- - 0
  - 1-4
  - 5-9
  - 10 of meer

Dag 2

- - 0
  - 1-4
  - 5-9
  - 10 of meer

Wij danken u nogmaals voor uw deelname.

Alle gegevens van dit dagboekje zullen confidentieel behandeld worden en zullen enkel gebruikt worden voor het wetenschappelijk onderzoek volgens toepassing van de wet ter bescherming van de persoonlijke levenssfeer.


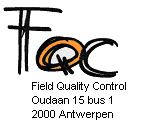


🖀 03-231 06 67

🖀 0800-93667
