# Supplementary material for: Mining social mixing patterns for infectious disease models based on a two-day population survey in Belgium
Source: BMC Infect Dis. 2009 Jan 20;9:5. doi: 10.1186/1471-2334-9-5 (PMC2656518; doi:10.1186/1471-2334-9-5)
Supplement: Additional file 6 — Diary Adults French. original diaries in French for adults. [file 1471-2334-9-5-S6.doc]

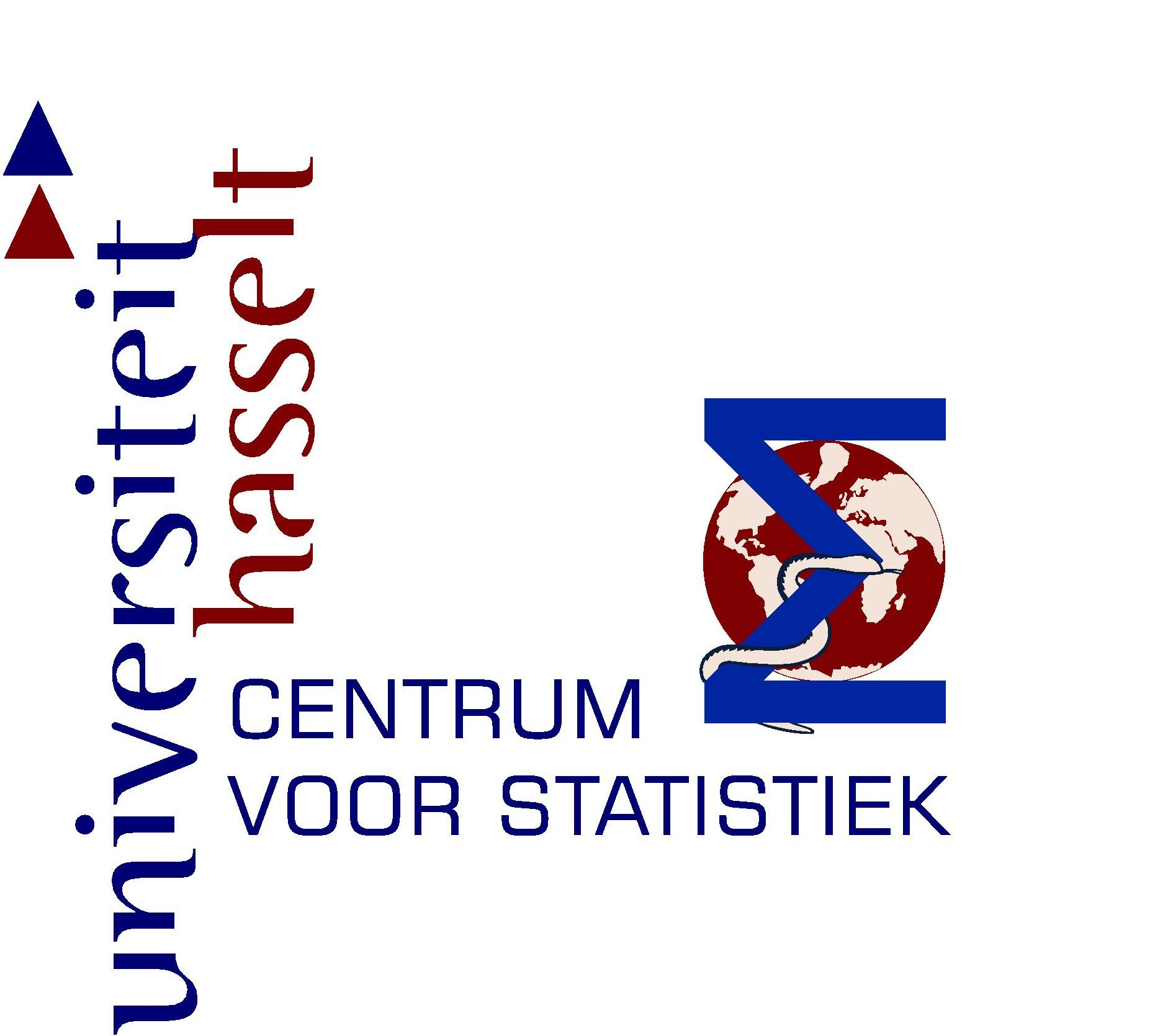


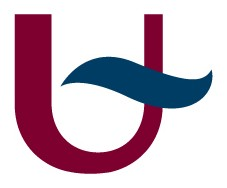


**Universiteit Antwerpen**


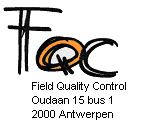


### Petit journal

### étude de contact

Si vous avez des problèmes ou des questions concernant le journal, n’hésitez pas à nous contacter au:

**03-231 06 67** ou **0800-93667**

Marie-Paule Feremans – Dave Van Ginkel

#### N°

##### Jour 1, date / /

Jour 2, date / /

**Comment remplir ce journal?**

- Nous vous prions d’indiquer dans ce journal toutes les personnes avec lesquelles vous avez été en contact direct et que vous avez rencontrées au cours des deux journées que nous vous avons attribuées.
- Un contact veut dire que vous avez parlé avec quelqu’un en sa présence physique (contacts par téléphone, GSM ou internet exclues). Un contact peut aussi être physique: toucher la peau de l’autre personne (se donner la main, donner une bise, des câlins ou en faisant du sport).
  - Les contacts avec des animaux ne comptent pas.
  - Important: il ne faut pas prendre en compte les personnes que vous avez uniquement contactées par téléphone ou GSM.
  - Si vous ne connaissez pas l’âge exact de la personne contactée, veuillez donner une estimation aussi précise que possible (p.ex. 40-45 ans).
- Veuillez utiliser une seule ligne par personne contactée: si vous avez contacté la même personne plusieurs fois dans la journée, ne l’indiquez qu’une seule fois et veuillez estimer le temps total que vous avez passé ensemble lors de la journée attribuée.
  - Votre tâche sera le plus facile si vous marquez vos contacts dans ce journal au fur et à mesure au cours de la journée (tous les 2 heures p.ex.).
- Le plus facile est d’essayer d’inscrire vos contacts par ordre chronologique en commençant par la personne que vous avez rencontrée en premier lors de la journée attribuée et puis de continuer avec toutes les autres personnes dont vous vous souvenez, en fonction des activités de la journée.
- Quand vous pensez avoir terminé la liste de vos contacts, nous vous demandons de bien réfléchir encore une fois afin de vérifier que vous n’avez pas oublié une activité où vous auriez pu avoir un contact. Votre agenda pourra être utile à cet effet.
- Pour les besoins de notre étude, la journée commence à 5 heures le matin de la journée attribuée et finit à 5 heures le lendemain matin.

Merci de fournir quelques données personnelles

1. Age ans
2. Sexe ⬜ féminin ⬜ masculin
3. Situation professionnelle:
   - indépendant (artisan, commerçant, fermier, …)
   - cadre supérieur ou profession libérale (avocat, médecin, architecte, …)
   - employé(e)
   - ouvrier(ère)
   - retraité(e)
   - au foyer
   - étudiant(e)
   - à la recherche d’emploi
   - autre
4. Niveau d’éducation (quelles études avez-vous terminé):

⬜ Aucune étude

⬜ Enseignement primaire

⬜ Enseignement professionnel

⬜ Enseignement technique inférieur

⬜ Enseignement général inférieur

⬜ Enseignement technique secondaire

⬜ Enseignement général secondaire

⬜ Enseignement supérieur non universitaire

⬜ Enseignement universitaire

1. Nationalité:
   - belge
   - autre de l’Union européenne
   - autre en dehors de l’Union européenne
2. Le nombre de personnes dans votre ménage (sans vous-même):
3. L’âge des membres de votre ménage (sans vous-même), en commençant par le plus jeune: , , , , , , , , , , ,
4. Lieu de résidence 9. Code postal
5. Est-ce que vous exercez une profession qui entraîne beaucoup de contacts (clients,

patients, étudiants, …)? ⬜ oui ⬜ non

**Si oui**, veuillez estimer en moyenne le nombre de personnes (clients, patients, étudiants,...) que vous rencontrez par jour:

Ces contacts professionnels se situent plutôt dans les groupes suivantes (plusieurs possibilités): ⬜ 0-5 ans ⬜ 6-11 ans ⬜ 12-17 ans ⬜ 18-60 ans ⬜ plus de 60 ans

Si vous avez estimé le nombre de ces contacts à plus de 20, nous vous prions de ne pas énumérer vos contacts professionnels dans votre journal, et de seulement indiquer les autres contacts.

# Exemple

| Age (ou fourchette) | Sexe ♀ ♂  féminin masculin | Lieu de contact (choix multiples possibles)  crèche,  maternelle, transport  école, (voitures,  à la lieu de lycée, train,  maison travail université bus, …) loisirs autres |
| --- | --- | --- |
| (- )  9  (- )  2  5  3  0 | X  X | X  X  X |

Première ligne: vous avez parlé pendant dix minutes avec votre fils de 9 ans en le conduisant à l’école le matin. Le soir vous avez joué ensemble entre 18-20 heures et vous lui avez donné une bise avant d’aller au lit.

Deuxième ligne: vous avez parlé avec une jeune vendeuse dans votre magasin de chaussures préféré, où vous allez plusieurs fois par an. Aujourd’hui vous avez essayé plusieurs pairs de chaussures.

| A quelle fréquence rencontrez-vous cette personne  (presque) quelques quelques quelques 1ère  chaque fois par fois par fois par an fois  jour semaine mois ou moins  souvent | Avez-vous touché sa peau?  (p.ex. se donner la main,  bises, sport)    oui non | Durée totale passée avec la personne      moins 5-15 15 min 1-4h 4h ou  de min -1h plus  5 min |
| --- | --- | --- |
| X  X | X  X | X  X |

Date jour 1 / /

**Liste de personnes avec lesquelles vous étiez en contact pendant cette**

| Age (ou fourchette) | Sexe ♀ ♂  féminin masculin | Lieu de contact (choix multiples possibles)  crèche, transport  maternelle, (voitures,  à la lieu de école, train, loisirs autres  maison travail lycée, bus, …)  université |
| --- | --- | --- |
| (- )  (- )  (- )  (- )  (- )  (- )  (- )  (- )  (- )  (- )  (- )  (- )  (- )  (- )  (- ) |  |  |

**première journée attribuée entre 5 heures et 5 heures le lendemain matin**

| A quelle fréquence rencontrez-vous cette personne  (presque) quelques quelques quelques 1ère  chaque fois par fois par fois par an fois  jour semaine mois ou moins  souvent | Avez-vous touché sa peau?  (p.ex. se donner la main,  bises, sport)    oui non | Durée totale passée avec la personne  moins 5-15 15 min 1-4h 4h  de min - 1 h ou  5 min plus |
| --- | --- | --- |
|  |  |  |

Date jour 1 / /

**Liste de personnes avec lesquelles vous étiez en contact pendant cette**

| Age (ou fourchette) | Sexe ♀ ♂  féminin masculin | Lieu de contact (choix multiples possibles)  crèche, transport  maternelle, (voitures,  à la lieu de école, train, loisirs autres  maison travail lycée, bus, …)  université |
| --- | --- | --- |
| (- )  (- )  (- )  (- )  (- )  (- )  (- )  (- )  (- )  (- )  (- )  (- )  (- )  (- )  (- ) |  |  |

**première journée attribuée entre 5 heures et 5 heures le lendemain matin**

| A quelle fréquence rencontrez-vous cette personne  (presque) quelques quelques quelques 1ère  chaque fois par fois par fois par an fois  jour semaine mois ou moins  souvent | Avez-vous touché sa peau?  (p.ex. se donner la main,  bises, sport)    oui non | Durée totale passée avec la personne  moins 5-15 15 min 1-4h 4h  de min - 1 h ou  5 min plus |
| --- | --- | --- |
|  |  |  |

Date jour 1 / /

**Liste de personnes avec lesquelles vous étiez en contact pendant cette**

| Age (ou fourchette) | Sexe ♀ ♂  féminin masculin | Lieu de contact (choix multiples possibles)  crèche, transport  maternelle, (voitures,  à la lieu de école, train, loisirs autres  maison travail lycée, bus, …)  université |
| --- | --- | --- |
| (- )  (- )  (- )  (- )  (- )  (- )  (- )  (- )  (- )  (- )  (- )  (- )  (- )  (- )  (- ) |  |  |

**première journée attribuée entre 5 heures et 5 heures le lendemain matin**

| A quelle fréquence rencontrez-vous cette personne  (presque) quelques quelques quelques 1ère  chaque fois par fois par fois par an fois  jour semaine mois ou moins  souvent | Avez-vous touché sa peau?  (p.ex. se donner la main,  bises, sport)    oui non | Durée totale passée avec la personne  moins 5-15 15 min 1-4h 4h  de min - 1 h ou  5 min plus |
| --- | --- | --- |
|  |  |  |

Date jour 1 / /

**Liste de personnes avec lesquelles vous étiez en contact pendant cette**

| Age (ou fourchette) | Sexe ♀ ♂  féminin masculin | Lieu de contact (choix multiples possibles)  crèche, transport  maternelle, (voitures,  à la lieu de école, train, loisirs autres  maison travail lycée, bus, …)  université |
| --- | --- | --- |
| (- )  (- )  (- )  (- )  (- )  (- )  (- )  (- )  (- )  (- )  (- )  (- )  (- )  (- )  (- ) |  |  |

**première journée attribuée entre 5 heures et 5 heures le lendemain matin**

| A quelle fréquence rencontrez-vous cette personne  (presque) quelques quelques quelques 1ère  chaque fois par fois par fois par an fois  jour semaine mois ou moins  souvent | Avez-vous touché sa peau?  (p.ex. se donner la main,  bises, sport)    oui non | Durée totale passée avec la personne  moins 5-15 15 min 1-4h 4h  de min - 1 h ou  5 min plus |
| --- | --- | --- |
|  |  |  |

Date jour 1 / /

**Liste de personnes avec lesquelles vous étiez en contact pendant cette**

| Age (ou fourchette) | Sexe ♀ ♂  féminin masculin | Lieu de contact (choix multiples possibles)  crèche, transport  maternelle, (voitures,  à la lieu de école, train, loisirs autres  maison travail lycée, bus, …)  université |
| --- | --- | --- |
| (- )  (- )  (- )  (- )  (- )  (- )  (- )  (- )  (- )  (- )  (- )  (- )  (- )  (- )  (- ) |  |  |

**première journée attribuée entre 5 heures et 5 heures le lendemain matin**

| A quelle fréquence rencontrez-vous cette personne  (presque) quelques quelques quelques 1ère  chaque fois par fois par fois par an fois  jour semaine mois ou moins  souvent | Avez-vous touché sa peau?  (p.ex. se donner la main,  bises, sport)    oui non | Durée totale passée avec la personne  moins 5-15 15 min 1-4h 4h  de min - 1 h ou  5 min plus |
| --- | --- | --- |
|  |  |  |

Date jour 1 / /

**Liste de personnes avec lesquelles vous étiez en contact pendant cette**

| Age (ou fourchette) | Sexe ♀ ♂  féminin masculin | Lieu de contact (choix multiples possibles)  crèche, transport  maternelle, (voitures,  à la lieu de école, train, loisirs autres  maison travail lycée, bus, …)  université |
| --- | --- | --- |
| (- )  (- )  (- )  (- )  (- )  (- )  (- )  (- )  (- )  (- )  (- )  (- )  (- )  (- )  (- ) |  |  |

**première journée attribuée entre 5 heures et 5 heures le lendemain matin**

| A quelle fréquence rencontrez-vous cette personne  (presque) quelques quelques quelques 1ère  chaque fois par fois par fois par an fois  jour semaine mois ou moins  souvent | Avez-vous touché sa peau?  (p.ex. se donner la main,  bises, sport)    oui non | Durée totale passée avec la personne  moins 5-15 15 min 1-4h 4h  de min - 1 h ou  5 min plus |
| --- | --- | --- |
|  |  |  |

Date jour 2 / /

**Liste de personnes avec lesquelles vous étiez en contact pendant cette**

| Age (ou fourchette) | Sexe ♀ ♂  féminin masculin | Lieu de contact (choix multiples possibles)  crèche, transport  maternelle, (voitures,  à la lieu de école, train, loisirs autres  maison travail lycée, bus, …)  université |
| --- | --- | --- |
| (- )  (- )  (- )  (- )  (- )  (- )  (- )  (- )  (- )  (- )  (- )  (- )  (- )  (- )  (- ) |  |  |

**deuxième journée attribuée entre 5 heures et 5 heures le lendemain matin**

| A quelle fréquence rencontrez-vous cette personne  (presque) quelques quelques quelques 1ère  chaque fois par fois par fois par an fois  jour semaine mois ou moins  souvent | Avez-vous touché sa peau?  (p.ex. se donner la main,  bises, sport)    oui non | Durée totale passée avec la personne  moins 5-15 15 min 1-4h 4h  de min - 1 h ou  5 min plus |
| --- | --- | --- |
|  |  |  |

Date jour 2 / /

**Liste de personnes avec lesquelles vous étiez en contact pendant cette**

| Age (ou fourchette) | Sexe ♀ ♂  féminin masculin | Lieu de contact (choix multiples possibles)  crèche, transport  maternelle, (voitures,  à la lieu de école, train, loisirs autres  maison travail lycée, bus, …)  université |
| --- | --- | --- |
| (- )  (- )  (- )  (- )  (- )  (- )  (- )  (- )  (- )  (- )  (- )  (- )  (- )  (- )  (- ) |  |  |

**deuxième journée attribuée entre 5 heures et 5 heures le lendemain matin**

| A quelle fréquence rencontrez-vous cette personne  (presque) quelques quelques quelques 1ère  chaque fois par fois par fois par an fois  jour semaine mois ou moins  souvent | Avez-vous touché sa peau?  (p.ex. se donner la main,  bises, sport)    oui non | Durée totale passée avec la personne  moins 5-15 15 min 1-4h 4h  de min - 1 h ou  5 min plus |
| --- | --- | --- |
|  |  |  |

Date jour 2 / /

**Liste de personnes avec lesquelles vous étiez en contact pendant cette**

| Age (ou fourchette) | Sexe ♀ ♂  féminin masculin | Lieu de contact (choix multiples possibles)  crèche, transport  maternelle, (voitures,  à la lieu de école, train, loisirs autres  maison travail lycée, bus, …)  université |
| --- | --- | --- |
| (- )  (- )  (- )  (- )  (- )  (- )  (- )  (- )  (- )  (- )  (- )  (- )  (- )  (- )  (- ) |  |  |

**deuxième journée attribuée entre 5 heures à 5 heures le lendemain matin**

| A quelle fréquence rencontrez-vous cette personne  (presque) quelques quelques quelques 1ère  chaque fois par fois par fois par an fois  jour semaine mois ou moins  souvent | Avez-vous touché sa peau?  (p.ex. se donner la main,  bises, sport)    oui non | Durée totale passée avec la personne  moins 5-15 15 min 1-4h 4h  de min - 1 h ou  5 min plus |
| --- | --- | --- |
|  |  |  |

Date jour 2 / /

**Liste de personnes avec lesquelles vous étiez en contact pendant cette**

| Age (ou fourchette) | Sexe ♀ ♂  féminin masculin | Lieu de contact (choix multiples possibles)  crèche, transport  maternelle, (voitures,  à la lieu de école, train, loisirs autres  maison travail lycée, bus, …)  université |
| --- | --- | --- |
| (- )  (- )  (- )  (- )  (- )  (- )  (- )  (- )  (- )  (- )  (- )  (- )  (- )  (- )  (- ) |  |  |

**deuxième journée attribuée entre 5 heures et 5 heures le lendemain matin**

| A quelle fréquence rencontrez-vous cette personne  (presque) quelques quelques quelques 1ère  chaque fois par fois par fois par an fois  jour semaine mois ou moins  souvent | Avez-vous touché sa peau?  (p.ex. se donner la main,  bises, sport)    oui non | Durée totale passée avec la personne  moins 5-15 15 min 1-4h 4h  de min - 1 h ou  5 min plus |
| --- | --- | --- |
|  |  |  |

Date jour 2 / /

**Liste de personnes avec lesquelles vous étiez en contact pendant cette**

| Age (ou fourchette) | Sexe ♀ ♂  féminin masculin | Lieu de contact (choix multiples possibles)  crèche, transport  maternelle, (voitures,  à la lieu de école, train, loisirs autres  maison travail lycée, bus, …)  université |
| --- | --- | --- |
| (- )  (- )  (- )  (- )  (- )  (- )  (- )  (- )  (- )  (- )  (- )  (- )  (- )  (- )  (- ) |  |  |

**deuxième journée attribuée entre 5 heures et 5 heures le lendemain matin**

| A quelle fréquence rencontrez-vous cette personne  (presque) quelques quelques quelques 1ère  chaque fois par fois par fois par an fois  jour semaine mois ou moins  souvent | Avez-vous touché sa peau?  (p.ex. se donner la main,  bises, sport)    oui non | Durée totale passée avec la personne  moins 5-15 15 min 1-4h 4h  de min - 1 h ou  5 min plus |
| --- | --- | --- |
|  |  |  |

Date jour 2 / /

**Liste de personnes avec lesquelles vous étiez en contact pendant cette**

| Age (ou fourchette) | Sexe ♀ ♂  féminin masculin | Lieu de contact (choix multiples possibles)  crèche, transport  maternelle, (voitures,  à la lieu de école, train, loisirs autres  maison travail lycée, bus, …)  université |
| --- | --- | --- |
| (- )  (- )  (- )  (- )  (- )  (- )  (- )  (- )  (- )  (- )  (- )  (- )  (- )  (- )  (- ) |  |  |

**deuxième journée attribuée entre 5 heures et 5 heures le lendemain matin**

| A quelle fréquence rencontrez-vous cette personne  (presque) quelques quelques quelques 1ère  chaque fois par fois par fois par an fois  jour semaine mois ou moins  souvent | Avez-vous touché sa peau?  (p.ex. se donner la main,  bises, sport)    oui non | Durée totale passée avec la personne  moins 5-15 15 min 1-4h 4h  de min - 1 h ou  5 min plus |
| --- | --- | --- |
|  |  |  |

1. Avez-vous eu des problèmes pour remplir ce journal? Si oui, merci de les préciser.
2. Avez-vous complété le journal au fur et à mesure pendant les journées en question (en le gardant avec vous) ou uniquement le soir?

Journée 1

⬜ pendant la journée

⬜ le soir

⬜ autre, à spécifier

Journée 2

⬜ pendant la journée

⬜ le soir

⬜ autre, à spécifier

1. Combien de contacts pensez-vous ne pas avoir énumérés, soit parce que vous les avez oubliés ou parce qu’il y en avait trop?

Journée 1

⬜ 0

⬜ 1-4

⬜ 5-9

⬜ 10 ou plus

Journée 2

⬜ 0

⬜ 1-4

⬜ 5-9

⬜ 10 ou plus

Nous vous remercions encore une fois de votre participation.

Toutes les informations de ce journal seront traitées de façon confidentielle et ne seront utilisées qu’à des fins de recherche scientifique.


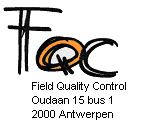


🖀 03-231 06 67

🖀 0800-93667
