# Supplementary material for: Mining social mixing patterns for infectious disease models based on a two-day population survey in Belgium
Source: BMC Infect Dis. 2009 Jan 20;9:5. doi: 10.1186/1471-2334-9-5 (PMC2656518; doi:10.1186/1471-2334-9-5)
Supplement: Additional file 7 — Data dictionary. English data dictionary of the diaries. [file 1471-2334-9-5-S7.doc]

# Appendix II: Belgian Contact Study Dictionary

**Notes & explanations:**

- Abbreviations:
  - cnt_ stands for contact and refers to the person being contacted by the person whom the diary refers to
  - hh_ stands for household
- Non-response field has been coded as empty , i.e. contain nothing, these entries have been considered as missing values in the analysis
- The data is organized into 3 ASCII files:
  - The file “participantsfull1.txt” with the information relating to the person whom the diary refers to
    - One line per participant 🡪 variables referring to day 1 have an index 1 and variables referring to day 2 have an index 2.
  - The file “participantsfull2.txt” with the information relating to the person whom the diary refers to
    - Two lines per participant 🡪 one line for each day.
  - The file “allcontacts.txt” with the contact data is linkable to the participantsfull2.txt file through the identifier variables “local_id” and “firstorsec_day”
    - One line per contacted person (colour coded in yellow).
- Data are tab-separated text files
- Data are available from the authors upon request

Variable dictionary for “participantsfull1.txt” (with indices) and “participantsfull2.txt”

| **Variable name** | **Variable label** | **Variable**  **Type** | **Value labels** |
| --- | --- | --- | --- |
| local_id | Local identifying number of the diary (initial) | Integer |  |
| country | Country where survey was carried out | String of 2 chars | BE - Belgium |
| firstorsec_day* | Which day the variables refer to | Integer | 1 : day 1  2 : day 2 |
| type | Who filled the diary in? | Integer | 1 : Adult, himself  2 : Teenager, older Child. himself  3 : Parent on behalf of child |
| participant_age | Age of person whom the diary refers to (adult or child) | Integer |  |
| participant _gender | Gender of the person whom the diary refers to (adult or child) | String 1 char | F : Female  M : Male |
| participant _occupation | Occupational status of the person filling in the diary | Integer | 1: working  2: retired  3: at home (housewife)  4: currently unemployed / job seeking  5: in fulltime or further education  6: other |
| participant _occ_detail | Details of the occupational status of the person filling in the diary, if available | Integer | 5: entrepreneur/self-employed  6 : upper employee (kader, vrij beroep)  7 : lower employee (bediende)  8: worker |
| participant _education | Educational level of the person filling in the diary  (only if diary=1 or 3) | Integer | 0: no formal schooling  1: primary school  2: secondary school (lower)  3: upper secondary school (upper)  4: secondary school (unspecified)  7: university degree (unspecified)  8: diary type == 2 (partdata2) |
| participant_edu_length | Corresponding total number of years of education (average) (only if diary=1 or 3) | Integer |  |
| participant _school_year | School year teenage/older child respondent attend  (only if diary_type=2, i.e. teenager) | Integer |  |
| participant _nationality | Nationality of the person filling in the diary | String 2 chars | BE – Belgium  EU – other EU countries  OT – other non-EU countries |
| hh_ size | Household size **including** participant | Integer |  |
| hh_age_1 | Age of household member 1 | Integer |  |
| hh_age_2 | Age of household member 2 | Integer |  |
| hh_age_3 | Age of household member 3 | Integer |  |
| hh_age_4 | Age of household member 4 | Integer |  |
| hh_age_5 | Age of household member 5 | Integer |  |
| hh_age_6 | Age of household member 6 | Integer |  |
| hh_age_7 | Age of household member 7 | Integer |  |
| hh_age_8 | Age of household member 8 | Integer |  |
| hh_age_9 | Age of household member 9 | Integer |  |
| hh_age_10 | Age of household member 10 | Integer |  |
| hh_age_11 | Age of household member 11 | Integer |  |
| hh_age_12 | Age of household member 12 | Integer |  |
| hh_age_13 | Age of household member 13 | Integer |  |
| hh_age_14 | Age of household member 14 | Integer |  |
| hh_age_15 | Age of household member 15 | Integer |  |
| hh_age_16 | Age of household member 16 | Integer |  |
| hh_age_17 | Age of household member 17 | Integer |  |
| hh_age_18 | Age of household member 18 | Integer |  |
| hh_age_19 | Age of household member 19 | Integer |  |
| hh_age_20 | Age of household member 20 | Integer |  |
| class_size  (if diary_type=2) | How many persons do you share a classroom with?  (if diary_type==2) | Integer |  |
| work_contacts | Do you have many contacts at work?  (if diary_type==1 or 2) | String 1 char | Y : Yes  N : No |
| work_contacts_nr | How many contacts at work?  (if diary_type==1 or 2) | Integer |  |
| work_age_1 | Are the contacts at work in age class 0-5? | Boolean | 0 : No  1 : Yes |
| work_age_2 | Are the contacts at work in age class 6-11? | Boolean | 0 : No  1 : Yes |
| work_age_3 | Are the contacts at work in age class 12-17? | Boolean | 0 : No  1 : Yes |
| work_age_4 | Are the contacts at work in age class 18-60? | Boolean | 0 : No  1 : Yes |
| work_age_5 | Are the contacts at work in age class 60+? | Boolean | 0 : No  1 : Yes |
| fill_in_age | Age of the person filling in the diary (only if diary_type=3) | Integer |  |
| fill_in_gender | Gender of the person filling in the diary (only if diary_type=3) | String 1 char | F : Female  M : Male |
| child_care | Does the child attend day care or school? | String 1 char | Y : Yes  N : No |
| child_care_nr | How many children at day care or school on average? | Integer | 1: less than 10  2: between 10 and 20  3: more than 20 |
| child_relationship | Relationship of child to person filling in the diary | Integer | 1: Mother  2: Father  3: Sibling  4: Grandparent  5: Other |
| child_nationality | Nationality of the child whom the diary refers to | String 2 chars | BE - Belgium  EU – other EU countries  OT – other non-EU countries |
| problems | Problems filling in the diary | String 1 char | Y : Yes  N : No |
| day | Day diary was filled in | Integer |  |
| month | Month diary was filled in | Integer |  |
| year | Year diary was filled in | Integer |  |
| dayofweek | Day of the week of filling in the diary | Integer | 0 : Sunday  1 : Monday  2 : Tuesday  3 : Wednesday  4 : Thursday  5 : Friday  6 : Saturday |
| work_day | Was the diary filled in on a weekday? | Boolean | 0 : No  1 : Yes |
| holiday | Was the diary filled in during holiday (Easter holiday from 1^st^ of April till 17^th^ of April 2006)? | Boolean | 0 : No  1 : Yes |
| diary_how | How was the diary filled in? | Integer | 1: during the day  2: in the evening  3: other |
| diary_missed_unsp | How many contacts were missed by the person filling in the diary (unspecified, whether physical or non-physical)? | Integer | 1: 0  2: 1-4  3: 5-9  4: 10 or more |

(*) This variable is only available in participantsfull2.txt

Variable dictionary for “allcontacts.txt”

| **Variable name** | **Variable label** | **Variable**  **Type** | **Value labels** |
| --- | --- | --- | --- |
| local_id | Local identifying number of the diary (initial) | Integer |  |
| country | Country where survey was carried out | String | BE - Belgium |
| firstorsec_day | Which day the variables refer to | Integer | 1 : day 1  2 : day 2 |
| cnt_age_mean | Age of the contacted person, average of cnt_age_l and cnt_age_r if both were specified | Integer |  |
| cnt_ age_l | Left limit of age range given of reported contact | Integer |  |
| cnt_age_r | Right limit of age range given of reported contact | Integer |  |
| cnt_sex | Gender of reported contact | String | F : Female  M : Male |
| cnt_home | Contact at home | Boolean |  |
| cnt_work | Contact at work | Boolean |  |
| cnt_school | Contact at school | Boolean |  |
| cnt_transport | Contact during transport | Boolean |  |
| cnt_leisure | Contact during leisure activ. | Boolean |  |
| cnt_otherplace | Contact during other activ. | Boolean |  |
| cnt_frequency | Frequency of contacting the reported contact | Integer | 1: daily  2: weekly  3: monthly  4: a few times a year  5: 1st time |
| cnt_touch | Was the contact physical? | Integer | 1 : Yes  2 : No |
| cnt_duration | Total duration of contact with reported person during the whole day | Integer | 1: less than 5 minutes  2 : 5 – 15 mins  3 : 15 mins – 1hour  4 : 1hour – 4hours  5 : 4 hours or more |
