# Supplementary material for: Mining social mixing patterns for infectious disease models based on a two-day population survey in Belgium
Source: BMC Infect Dis. 2009 Jan 20;9:5. doi: 10.1186/1471-2334-9-5 (PMC2656518; doi:10.1186/1471-2334-9-5)
Supplement: Additional file 12 — WGEE. The results of the weighted GEE analyses. [file 1471-2334-9-5-S12.doc]

# Appendix IV: Weighted GEE results for the different types of diaries.

Table IV.1.1-IV.1.4

Table IV.2.1-IV-2.4

Table IV.3.1-IV-3.4

***IV.1. WGEE-analysis on children (Type I diary)***

| **Children** |  | **N°** | **All Contacts** | |  | **Close Contacts** | | | **Non-close Contacts** | | |
| --- | --- | --- | --- | --- | --- | --- | --- | --- | --- | --- | --- |
|  |  |  | Rate | LCL | UCL | Rate | LCL | UCL | Rate | LCL | UCL |
|  | (Intercept) |  | 5.27 |  |  | 3.71 |  |  | 1.16 |  |  |
| Holiday period | Holiday | 235 | **0.73** | 0.58 | 0.90 | **0.70** | 0.54 | 0.91 |  |  |  |
| Region | Flanders | 168 | 0.94 | 0.69 | 1.27 | 1.20 | 0.92 | 1.57 | 0.58 | 0.32 | 1.04 |
| (BL: Brussels) | Wallonia | 80 | **0.72** | 0.52 | 0.99 | 0.99 | 0.74 | 1.33 | **0.34** | 0.17 | 0.66 |
| Gender | Male | 140 | 1.13 | 0.92 | 1.37 | 1.20 | 0.97 | 1.49 |  |  |  |
| Age | 6-8 years | 94 |  |  |  |  |  |  |  |  |  |
| Child Care | 11-20 children | 34 | **1.42** | 1.06 | 1.90 | 1.16 | 0.84 | 1.59 | **4.15** | 1.51 | 11.41 |
| (BL: 1-10 children) | 20+ children | 168 | **1.50** | 1.19 | 1.90 | 1.12 | 0.88 | 1.44 | **5.46** | 2.24 | 13.35 |
|  | No childcare* | 80 | 1.15 | 0.92 | 1.43 | 1.02 | 0.82 | 1.28 | **2.58** | 1.07 | 6.22 |
| Household Size |  |  | **1.18** | 1.10 | 1.26 | **1.18** | 1.09 | 1.28 |  |  |  |
| Overdispersion + s.e. |  |  |  | **4.04** | 0.64 |  | **2.97** | 0.43 |  | **3.58** | 0.46 |
| Correlation + s.e. |  |  |  | 0.04 | 0.12 |  | 0.10 | 0.11 |  | -0.12 | 0.09 |
| * No child care includes the weekend period | | |  |  |  |  |  |  |  |  |  |

Table IV.1.1: Final weighted GEE model for all, close and non-close contacts for type I diary. For each of the discrete variables, the baseline category is shown. In the third column, the number of observations in each category is given. The rate should be interpreted as a factor versus the baseline and the intercept rate and is given together with the 95% confidence limits (LCL, UCL).

| **Children** |  | **N°** | **Home Contacts** | | | **Work Contacts***** | | | **School Contacts**** | | | **Leisure Contacts** | | | **Transport Contacts** | | | **Other Contacts** | | |
| --- | --- | --- | --- | --- | --- | --- | --- | --- | --- | --- | --- | --- | --- | --- | --- | --- | --- | --- | --- | --- |
|  |  |  | Rate | LCL | UCL | Rate | LCL | UCL | Rate | LCL | UCL | Rate | LCL | UCL | Rate | LCL | UCL | Rate | LCL | UCL |
|  | (Intercept) |  | 1.40 |  |  |  |  |  | 2.24 |  |  | 0.48 |  |  | 0.12 |  |  | 1.51 |  |  |
| Holiday period | Holiday | 235 |  |  |  |  |  |  |  |  |  | **2.38** | 1.22 | 4.64 |  |  |  |  |  |  |
| Region | Flanders | 168 | 0.78 | 0.57 | 1.06 |  |  |  |  |  |  | **3.03** | 1.17 | 7.85 |  |  |  |  |  |  |
| (BL: Brussels) | Wallonia | 80 | 1.06 | 0.75 | 1.50 |  |  |  |  |  |  | 0.83 | 0.31 | 2.23 |  |  |  |  |  |  |
| Gender | Male | 140 |  |  |  |  |  |  |  |  |  |  |  |  |  |  |  |  |  |  |
| Age | 6-8 years | 94 |  |  |  |  |  |  |  |  |  |  |  |  |  |  |  |  |  |  |
| Child Care | 11-20 children | 34 | 1.51 | 0.99 | 2.31 |  |  |  | **2.41** | 1.14 | 5.10 |  |  |  |  |  |  |  |  |  |
| (BL: 1-10 children) | 20+ children | 168 | **1.72** | 1.13 | 2.60 |  |  |  | **3.29** | 1.36 | 7.96 |  |  |  |  |  |  |  |  |  |
|  | No childcare* | 80 | **1.92** | 1.32 | 2.81 |  |  |  | **0.00** | 0.00 | 0.00 |  |  |  |  |  |  |  |  |  |
| Household Size |  |  | 1.07 | 0.92 | 1.23 |  |  |  | 1.17 | 0.90 | 1.54 |  |  |  |  |  |  |  |  |  |
| Overdispersion + s.e. | |  |  | **1.68** | 0.29 |  |  |  |  | 11.28 | 6.37 |  | **8.06** | 1.57 |  | 1.84 | 0.98 |  | **3.95** | 1.03 |
| Correlation +s.e. |  |  |  | **0.46** | 0.09 |  |  |  |  | - | - |  | 0.01 | 0.10 |  | 0.21 | 0.18 |  | **0.31** | 0.14 |
| * No child care includes the weekend period | | |  |  |  |  |  |  |  |  |  |  |  |  |  |  |  |  |  |  |
| ** School contacts were only observed in the week during a regular period | | | | | | |  |  |  |  |  |  |  |  |  |  |  |  |  |  |
| *** Work contacts were not observed for children | | | |  |  |  |  |  |  |  |  |  |  |  |  |  |  |  |  |  |

Table IV.1.2: Final weighted GEE model for the different location contacts for type I diary. For each of the discrete variables, the baseline category is shown. In the third column, the number of observations in each category is given. The rate should be interpreted as a factor versus the baseline and the intercept rate and is given together with the 95% confidence limits (LCL, UCL).

| **Children** |  | **N°** | **Daily Contacts** | | | **Weekly Contacts** | | | **Monthly Contacts** | | | **Few Times a Year** | | | **First Time Contacts** | | |
| --- | --- | --- | --- | --- | --- | --- | --- | --- | --- | --- | --- | --- | --- | --- | --- | --- | --- |
|  |  |  | Rate | LCL | UCL | Rate | LCL | UCL | Rate | LCL | UCL | Rate | LCL | UCL | Rate | LCL | UCL |
|  | (Intercept) |  | 4.92 |  |  | 2.32 |  |  | 0.64 |  |  | 1.08 |  |  | 0.83 |  |  |
| Holiday period | Holiday | 235 |  |  |  |  |  |  |  |  |  |  |  |  |  |  |  |
| Region | Flanders | 168 |  |  |  |  |  |  | 1.63 | 0.91 | 2.94 |  |  |  |  |  |  |
| (BL: Brussels) | Wallonia | 80 |  |  |  |  |  |  | 0.81 | 0.42 | 1.55 |  |  |  |  |  |  |
| Gender | Male | 140 |  |  |  |  |  |  |  |  |  |  |  |  |  |  |  |
| Age | 6-8 years | 94 |  |  |  |  |  |  |  |  |  |  |  |  |  |  |  |
| Child Care | 11-20 children | 34 |  |  |  |  |  |  | 1.54 | 0.72 | 3.33 |  |  |  |  |  |  |
| (BL: 1-10 children) | 20+ children | 168 |  |  |  |  |  |  | 1.50 | 0.69 | 3.26 |  |  |  |  |  |  |
|  | No childcare* | 80 |  |  |  |  |  |  | **2.38** | 1.08 | 5.26 |  |  |  |  |  |  |
| Household Size |  |  |  |  |  |  |  |  |  |  |  |  |  |  |  |  |  |
| Overdispersion + s.e. |  |  |  | **6.13** | 1.98 |  | **4.81** | 1.02 |  | **3.06** | 0.52 |  | **7.06** | 1.82 |  | **4.28** | 0.58 |
| Correlation + s.e. |  |  |  | 0.00 | 0.04 |  | 0.12 | 0.07 |  | -0.06 | 0.09 |  | -0.02 | 0.11 |  | 0.14 | 0.11 |
| * No child care includes the weekend period | | |  |  |  |  |  |  |  |  |  |  |  |  |  |  |  |

Table IV.1.3: Final weighted GEE model for the different frequency contacts for type I diary. For each of the discrete variables, the baseline category is shown. In the third column, the number of observations in each category is given. The rate should be interpreted as a factor versus the baseline and the intercept rate and is given together with the 95% confidence limits (LCL, UCL).

| **Children** |  | **N°** | **Less than 5 min** | | | **5-15 min** | |  | **15 min-1 hour** | |  | **1-4 hours** | |  | **More than 4 hours** | | |
| --- | --- | --- | --- | --- | --- | --- | --- | --- | --- | --- | --- | --- | --- | --- | --- | --- | --- |
|  |  |  | Rate | LCL | UCL | Rate | LCL | UCL | Rate | LCL | UCL | Rate | LCL | UCL | Rate | LCL | UCL |
|  | (Intercept) |  | 0.08 |  |  | 0.40 |  |  | 0.40 |  |  | 2.23 |  |  | 2.72 |  |  |
| Holiday period | Holiday | 235 | **2.47** | 1.07 | 5.71 |  |  |  |  |  |  |  |  |  | **0.60** | 0.41 | 0.88 |
| Region | Flanders | 168 | **4.19** | 1.12 | 15.63 | **2.30** | 1.06 | 5.00 |  |  |  |  |  |  |  |  |  |
| (BL: Brussels) | Wallonia | 80 | 1.19 | 0.29 | 4.98 | 1.53 | 0.67 | 3.53 |  |  |  |  |  |  |  |  |  |
| Gender | Male | 140 |  |  |  |  |  |  |  |  |  |  |  |  |  |  |  |
| Age | 6-8 years | 94 |  |  |  |  |  |  | 1.47 | 0.96 | 2.25 | 0.90 | 0.58 | 1.40 |  |  |  |
| Child Care | 11-20 children | 34 |  |  |  |  |  |  |  |  |  |  |  |  | 1.23 | 0.66 | 2.30 |
| (BL: 1-10 children) | 20+ children | 168 |  |  |  |  |  |  |  |  |  |  |  |  | 1.19 | 0.70 | 2.01 |
|  | No childcare* | 80 |  |  |  |  |  |  |  |  |  |  |  |  | 0.85 | 0.54 | 1.36 |
| Household Size |  |  |  |  |  |  |  |  | **1.28** | 1.09 | 1.50 | 1.06 | 0.94 | 1.19 | **1.23** | 1.08 | 1.41 |
| Overdispersion + s.e. |  |  |  | **2.76** | 0.76 |  | **2.07** | 0.27 |  | **3.38** | 0.51 |  | **6.62** | 1.69 |  | **3.26** | 0.66 |
| Correlation + s.e. |  |  |  | 0.24 | 0.13 |  | 0.10 | 0.09 |  | 0.07 | 0.07 |  | 0.08 | 0.08 |  | **0.29** | 0.11 |
| * No child care includes the weekend period | |  |  |  |  |  |  |  |  |  |  |  |  |  |  |  |  |

Table IV.1.4: Final weighted GEE model for the different duration contacts for type I diary. For each of the discrete variables, the baseline category is shown. In the third column, the number of observations in each category is given. The rate should be interpreted as a factor versus the baseline and the intercept rate and is given together with the 95% confidence limits (LCL, UCL).

***2. WGEE-analysis on adolescents (Type II diary)***

| **Adolescents** |  | **N°** | **All Contacts** | |  | **Close Contacts** | | | **Non-close Contacts** | | |
| --- | --- | --- | --- | --- | --- | --- | --- | --- | --- | --- | --- |
|  |  |  | Rate | LCL | UCL | Rate | LCL | UCL | Rate | LCL | UCL |
|  | (Intercept) |  | 11.09 |  |  | 3.25 |  |  | 15.27 |  |  |
| Weekend | Yes | 19 | **0.34** | 0.22 | 0.53 |  |  |  | **0.24** | 0.14 | 0.43 |
| Holiday period | Holiday | 43 | **0.38** | 0.25 | 0.56 | 0.81 | 0.58 | 1.14 | **0.19** | 0.12 | 0.30 |
| Holiday & Weekend |  | 106 | **3.05** | 1.86 | 5.02 |  |  |  | **4.21** | 2.11 | 8.37 |
| Region | Flanders | 144 | 1.34 | 0.91 | 1.99 |  |  |  | 1.57 | 0.70 | 3.53 |
| (BL: Brussels) | Wallonia | 78 | 0.92 | 0.60 | 1.42 |  |  |  | 0.53 | 0.23 | 1.22 |
| First or Second Day | Second Day | 125 | **0.80** | 0.65 | 0.97 |  |  |  |  |  |  |
| Gender | Male | 126 |  |  |  |  |  |  |  |  |  |
| Age | 12-17 years | 184 |  |  |  |  |  |  |  |  |  |
| Class Size |  |  | 0.99 | 0.97 | 1.01 | **1.02** | 1.01 | 1.03 | 0.98 | 0.96 | 1.00 |
| Household Size |  |  | **1.25** | 1.11 | 1.41 | **1.28** | 1.14 | 1.45 |  |  |  |
| Overdispersion + s.e. |  |  |  | **7.02** | 1.11 |  | **6.33** | 1.26 |  | **6.40** | 1.10 |
| Correlation + s.e. |  |  |  | 0.18 | 0.12 |  | 0.30 | 0.18 |  | 0.08 | 0.09 |

Table IV.2.1: Final weighted GEE model for all, close and non-close contacts for type II diary. For each of the discrete variables, the baseline category is shown. In the third column, the number of observations in each category is given. The rate should be interpreted as a factor versus the baseline and the intercept rate and is given together with the 95% confidence limits (LCL, UCL).

| **Adolescents** |  | **N°** | **Home Contacts** | | | **Work Contacts*** | | | **School Contacts**** | | | **Leisure Contacts** | | | **Transport Contacts** | | | **Other Contacts** | | |
| --- | --- | --- | --- | --- | --- | --- | --- | --- | --- | --- | --- | --- | --- | --- | --- | --- | --- | --- | --- | --- |
|  |  |  | Rate | LCL | UCL | Rate | LCL | UCL | Rate | LCL | UCL | Rate | LCL | UCL | Rate | LCL | UCL | Rate | LCL | UCL |
|  | (Intercept) |  | 1.19 |  |  |  |  |  | 30.70 |  |  | 0.90 |  |  | 0.64 |  |  | 1.66 |  |  |
| Weekend | Yes | 19 |  |  |  |  |  |  |  |  |  | **1.49** | 1.02 | 2.17 |  |  |  |  |  |  |
| Holiday period | Holiday | 43 |  |  |  |  |  |  |  |  |  | **1.86** | 1.19 | 2.92 | **0.37** | 0.17 | 0.78 |  |  |  |
| Holiday & Weekend |  | 106 |  |  |  |  |  |  |  |  |  |  |  |  |  |  |  |  |  |  |
| Region | Flanders | 144 |  |  |  |  |  |  | 0.71 | 0.25 | 2.05 | **3.22** | 1.58 | 6.56 |  |  |  |  |  |  |
| (BL: Brussels) | Wallonia | 78 |  |  |  |  |  |  | 0.65 | 0.22 | 1.90 | 0.78 | 0.36 | 1.71 |  |  |  |  |  |  |
| First or Second Day | Second Day | 125 |  |  |  |  |  |  | 1.35 | 0.84 | 2.18 | **0.66** | 0.46 | 0.96 |  |  |  |  |  |  |
| Gender | Male | 126 |  |  |  |  |  |  |  |  |  |  |  |  |  |  |  |  |  |  |
| Age | 12-17 years | 184 |  |  |  |  |  |  |  |  |  |  |  |  |  |  |  |  |  |  |
| Class Size |  |  | **1.32** | 1.19 | 1.46 |  |  |  | 0.98 | 0.95 | 1.00 |  |  |  |  |  |  |  |  |  |
| Household Size |  |  | 0.99 | 0.98 | 1.00 |  |  |  |  |  |  |  |  |  |  |  |  |  |  |  |
| Overdispersion + s.e. |  |  |  | **1.63** | 0.36 |  |  |  |  | **8.00** | 1.77 |  | **8.49** | 1.68 |  | **2.38** | 0.46 |  | **5.04** | 1.51 |
| Correlation + s.e. |  |  |  | 0.22 | 0.15 |  |  |  |  | - | - |  | **0.23** | 0.10 |  | 0.14 | 0.17 |  | 0.74 | 0.42 |
| * Work contacts were not analyzed | |  |  |  |  |  |  |  |  |  |  |  |  |  |  |  |  |  |  |  |
| ** Excluded weekend and holiday period | | |  |  |  |  |  |  |  |  |  |  |  |  |  |  |  |  |  |  |

Table IV.2.2: Final weighted GEE model for the different location contacts for type II diary. For each of the discrete variables, the baseline category is shown. In the third column, the number of observations in each category is given. The rate should be interpreted as a factor versus the baseline and the intercept rate and is given together with the 95% confidence limits (LCL, UCL).

| **Adolescents** |  | **N°** | **Daily Contacts** | | | **Weekly Contacts** | | | **Monthly Contacts** | | | **Few Times a Year** | | | **First Time Contacts** | | |
| --- | --- | --- | --- | --- | --- | --- | --- | --- | --- | --- | --- | --- | --- | --- | --- | --- | --- |
|  |  |  | Rate | LCL | UCL | Rate | LCL | UCL | Rate | LCL | UCL | Rate | LCL | UCL | Rate | LCL | UCL |
|  | (Intercept) |  | 15.34 |  |  | 4.01 |  |  | 2.32 |  |  | 1.11 |  |  | 1.07 |  |  |
| Weekend | Yes | 19 | **0.26** | 0.19 | 0.37 | **0.46** | 0.32 | 0.64 |  |  |  |  |  |  |  |  |  |
| Holiday period | Holiday | 43 | **0.25** | 0.17 | 0.35 | **0.27** | 0.17 | 0.44 |  |  |  |  |  |  |  |  |  |
| Holiday & Weekend |  | 106 | **3.91** | 2.40 | 6.38 | **2.54** | 1.55 | 4.16 |  |  |  |  |  |  |  |  |  |
| Region | Flanders | 144 |  |  |  |  |  |  |  |  |  |  |  |  |  |  |  |
| (BL: Brussels) | Wallonia | 78 |  |  |  |  |  |  |  |  |  |  |  |  |  |  |  |
| First or Second Day | Second Day | 125 |  |  |  |  |  |  |  |  |  |  |  |  |  |  |  |
| Gender | Male | 126 |  |  |  |  |  |  |  |  |  |  |  |  |  |  |  |
| Age | 12-17 years | 184 |  |  |  | **2.65** | 1.60 | 4.40 |  |  |  |  |  |  |  |  |  |
| Class Size |  |  |  |  |  |  |  |  |  |  |  |  |  |  |  |  |  |
| Household Size |  |  |  |  |  |  |  |  |  |  |  |  |  |  |  |  |  |
| Overdispersion + s.e. |  |  |  | **5.05** | 1.32 |  | **5.83** | 0.98 |  | **6.31** | 2.00 |  | **11.39** | 5.64 |  | **4.33** | 0.67 |
| Correlation + s.e. |  |  |  | **0.20** | 0.10 |  | **0.36** | 0.13 |  | **-0.30** | 0.07 |  | **0.30** | 0.08 |  | 0.08 | 0.08 |

Table IV.2.3: Final weighted GEE model for the different frequency contacts for type II diary. For each of the discrete variables, the baseline category is shown. In the third column, the number of observations in each category is given. The rate should be interpreted as a factor versus the baseline and the intercept rate and is given together with the 95% confidence limits (LCL, UCL).

| **Adolescents** |  | **N°** | **Less than 5 min** | | | **5-15 min** | |  | **15 min-1 hour** | |  | **1-4 hours** | |  | **More than 4 hours** | | |
| --- | --- | --- | --- | --- | --- | --- | --- | --- | --- | --- | --- | --- | --- | --- | --- | --- | --- |
|  |  |  | Rate | LCL | UCL | Rate | LCL | UCL | Rate | LCL | UCL | Rate | LCL | UCL | Rate | LCL | UCL |
|  | (Intercept) |  | 1.36 |  |  | 4.23 |  |  | 1.25 |  |  | 3.71 |  |  | 5.42 |  |  |
| Weekend | Yes | 19 |  |  |  |  |  |  |  |  |  |  |  |  |  |  |  |
| Holiday period | Holiday | 43 |  |  |  | **0.28** | 0.17 | 0.46 | **0.38** | 0.23 | 0.64 | 0.80 | 0.58 | 1.10 |  |  |  |
| Holiday & Weekend |  | 106 |  |  |  |  |  |  |  |  |  |  |  |  |  |  |  |
| Region | Flanders | 144 |  |  |  |  |  |  |  |  |  | **1.81** | 1.19 | 2.76 |  |  |  |
| (BL: Brussels) | Wallonia | 78 |  |  |  |  |  |  |  |  |  | 1.19 | 0.77 | 1.85 |  |  |  |
| First or Second Day | Second Day | 125 |  |  |  |  |  |  |  |  |  | **0.57** | 0.42 | 0.77 |  |  |  |
| Gender | Male | 126 |  |  |  |  |  |  |  |  |  |  |  |  |  |  |  |
| Age | 12-17 years | 184 |  |  |  |  |  |  |  |  |  |  |  |  |  |  |  |
| Class Size |  |  |  |  |  |  |  |  |  |  |  |  |  |  |  |  |  |
| Household Size |  |  |  |  |  |  |  |  | 1.32 | 0.99 | 1.75 |  |  |  |  |  |  |
| Overdispersion + s.e. |  |  |  | **6.87** | 1.39 |  | **4.90** | 1.05 |  | **3.97** | 0.58 |  | **3.94** | 0.54 |  | **9.21** | 2.22 |
| Correlation + s.e. |  |  |  | -0.01 | 0.13 |  | **0.18** | 0.09 |  | **0.19** | 0.08 |  | 0.03 | 0.12 |  | 0.17 | 0.12 |

Table IV.2.4: Final weighted GEE model for the different duration contacts for type II diary. For each of the discrete variables, the baseline category is shown. In the third column, the number of observations in each category is given. The rate should be interpreted as a factor versus the baseline and the intercept rate and is given together with the 95% confidence limits (LCL, UCL).

***3. WGEE-analysis on adults (Type IV diary)***

| **Adults** |  | **N°** | **All Contacts** | |  | **Close Contacts** | | | **Non-close Contacts** | | |
| --- | --- | --- | --- | --- | --- | --- | --- | --- | --- | --- | --- |
|  |  |  | Rate | LCL | UCL | Rate | LCL | UCL | Rate | LCL | UCL |
|  | (Intercept) |  | 6.37 |  |  | 4.62 |  |  | 1.25 |  |  |
| Day of week | Tuesday | 29 | 1.15 | 0.81 | 1.63 | 1.29 | 0.80 | 2.07 | 1.19 | 0.75 | 1.89 |
| (BL: Monday) | Wednesday | 37 | 0.95 | 0.70 | 1.31 | 1.08 | 0.67 | 1.74 | 0.98 | 0.59 | 1.64 |
|  | Thursday | 73 | 0.81 | 0.60 | 1.10 | 0.77 | 0.52 | 1.15 | 0.93 | 0.66 | 1.31 |
|  | Friday | 108 | 0.93 | 0.68 | 1.27 | 1.05 | 0.72 | 1.51 | 0.93 | 0.69 | 1.27 |
|  | Saturday | 96 | **0.54** | 0.42 | 0.69 | **0.64** | 0.47 | 0.87 | **0.48** | 0.35 | 0.66 |
|  | Sunday | 71 | **0.46** | 0.35 | 0.59 | **0.54** | 0.40 | 0.73 | **0.39** | 0.27 | 0.57 |
| Holiday period | Holiday | 24 | 1.07 | 0.87 | 1.31 | 1.20 | 0.73 | 1.99 | 1.19 | 0.75 | 1.90 |
| (BL: Regular weekday) | Holiday Tuesday | 42 |  |  |  | 0.85 | 0.39 | 1.82 | 0.82 | 0.37 | 1.81 |
|  | Holiday Wednesday | 64 |  |  |  | 0.75 | 0.38 | 1.47 | 0.96 | 0.47 | 1.95 |
|  | Holiday Thursday | 32 |  |  |  | 0.97 | 0.49 | 1.94 | 0.70 | 0.35 | 1.38 |
|  | Holiday Friday | 7 |  |  |  | 0.61 | 0.23 | 1.65 | 0.67 | 0.27 | 1.67 |
|  | Holiday Saturday | 148 |  |  |  | 0.94 | 0.52 | 1.71 | 0.83 | 0.48 | 1.45 |
|  | Holiday Sunday | 164 |  |  |  | 1.01 | 0.58 | 1.77 | 0.79 | 0.43 | 1.44 |
| Region | Flanders | 882 | **1.84** | 1.29 | 2.61 | **1.52** | 1.04 | 2.22 | **2.37** | 1.56 | 3.63 |
| (BL: Brussels) | Wallonia | 478 | **1.70** | 1.21 | 2.38 | **1.74** | 1.20 | 2.52 | **1.70** | 1.09 | 2.65 |
| Occupation | Retired | 172 | **0.45** | 0.32 | 0.62 | **0.55** | 0.40 | 0.77 | **0.35** | 0.21 | 0.56 |
| (BL: Working) | At home | 88 | **0.50** | 0.37 | 0.68 | **0.60** | 0.46 | 0.78 | **0.42** | 0.26 | 0.70 |
|  | Unemployed | 76 | **0.53** | 0.40 | 0.72 | **0.60** | 0.45 | 0.78 | **0.46** | 0.31 | 0.67 |
|  | Further/fulltime education | 84 | 0.91 | 0.64 | 1.28 | 0.89 | 0.60 | 1.31 | 0.93 | 0.64 | 1.36 |
|  | Other | 70 | 0.76 | 0.52 | 1.11 | 0.93 | 0.61 | 1.41 | **0.60** | 0.38 | 0.96 |
| Age of participant | 25-44 years | 336 | 0.84 | 0.66 | 1.08 | 0.79 | 0.61 | 1.01 | 0.92 | 0.68 | 1.25 |
| (BL: 18-24 years) | 45-64 years | 368 | 1.07 | 0.80 | 1.42 | 0.93 | 0.70 | 1.24 | 1.23 | 0.88 | 1.71 |
|  | 65+ years | 96 | 1.00 | 0.63 | 1.57 | 0.99 | 0.64 | 1.52 | 0.88 | 0.44 | 1.73 |
| Education | Primary education | 36 | 1.63 | 0.78 | 3.41 | 1.03 | 0.44 | 2.38 | **3.90** | 1.29 | 11.79 |
| (BL: No formal education) | Secondary education | 498 | **1.96** | 1.01 | 3.81 | 1.36 | 0.69 | 2.69 | **3.94** | 1.39 | 11.10 |
|  | Higher education | 414 | 1.90 | 0.97 | 3.73 | 1.40 | 0.71 | 2.76 | **3.56** | 1.23 | 10.29 |
| Gender | Male | 426 | 0.96 | 0.81 | 1.12 | 1.04 | 0.87 | 1.25 |  |  |  |
| First or Second Day | Second Day | 479 | 0.98 | 0.81 | 1.18 | 0.95 | 0.79 | 1.15 |  |  |  |
| Household Size |  |  | **1.13** | 1.07 | 1.20 | **1.15** | 1.08 | 1.23 | **1.13** | 1.05 | 1.20 |
| Overdispersion + s.e. |  |  |  | **15.72** | 1.78 |  | **9.59** | 0.98 |  | **11.03** | 1.19 |
| Correlation + s.e. |  |  |  | 0.06 | 0.05 |  | 0.09 | 0.06 |  | **0.10** | 0.05 |

Table IV.3.1: Final weighted GEE model for all, close and non-close contacts for type IV diary. For each of the discrete variables, the baseline category is shown. In the third column, the number of observations in each category is given. The rate should be interpreted as a factor versus the baseline and the intercept rate and is given together with the 95% confidence limits (LCL, UCL).

| **Adults** |  | **N°** | **Home Contacts** | | | **Work Contacts*** | | | **School Contacts**** | | | **Leisure Contacts** | | | **Transport Contacts** | | | **Other Contacts** | | |
| --- | --- | --- | --- | --- | --- | --- | --- | --- | --- | --- | --- | --- | --- | --- | --- | --- | --- | --- | --- | --- |
|  |  |  | Rate | LCL | UCL | Rate | LCL | UCL | Rate | LCL | UCL | Rate | LCL | UCL | Rate | LCL | UCL | Rate | LCL | UCL |
|  | (Intercept) |  | 1.24 |  |  | 0.73 |  |  | 0.68 |  |  | 2.57 |  |  | 0.24 |  |  | 0.54 |  |  |
| Day of week | Tuesday | 29 | 1.02 | 0.64 | 1.62 | 1.27 | 0.73 | 2.21 |  |  |  |  |  |  |  |  |  |  |  |  |
| (BL: Monday) | Wednesday | 37 | 0.94 | 0.68 | 1.30 | 0.83 | 0.54 | 1.28 |  |  |  |  |  |  |  |  |  |  |  |  |
|  | Thursday | 73 | 0.73 | 0.53 | 1.01 | 0.77 | 0.49 | 1.22 |  |  |  |  |  |  |  |  |  |  |  |  |
|  | Friday | 108 | 0.90 | 0.65 | 1.24 | 0.80 | 0.51 | 1.26 |  |  |  |  |  |  |  |  |  |  |  |  |
|  | Saturday | 96 | 1.02 | 0.74 | 1.39 |  |  |  |  |  |  |  |  |  |  |  |  |  |  |  |
|  | Sunday | 71 | 0.91 | 0.64 | 1.28 |  |  |  |  |  |  |  |  |  |  |  |  |  |  |  |
| Holiday period | Holiday | 24 | 1.03 | 0.68 | 1.56 |  |  |  |  |  |  |  |  |  |  |  |  |  |  |  |
| (BL: Regular weekday) | Holiday Tuesday | 42 | **0.50** | 0.27 | 0.94 |  |  |  |  |  |  |  |  |  |  |  |  |  |  |  |
|  | Holiday Wednesday | 64 | 0.98 | 0.61 | 1.57 |  |  |  |  |  |  |  |  |  |  |  |  |  |  |  |
|  | Holiday Thursday | 32 | 0.90 | 0.53 | 1.54 |  |  |  |  |  |  |  |  |  |  |  |  |  |  |  |
|  | Holiday Friday | 7 | 0.78 | 0.39 | 1.56 |  |  |  |  |  |  |  |  |  |  |  |  |  |  |  |
|  | Holiday Saturday | 148 | 0.83 | 0.52 | 1.31 |  |  |  |  |  |  |  |  |  |  |  |  |  |  |  |
|  | Holiday Sunday | 164 | 1.01 | 0.60 | 1.70 |  |  |  |  |  |  |  |  |  |  |  |  |  |  |  |
| Region | Flanders | 882 | **1.37** | 1.06 | 1.77 |  |  |  |  |  |  | 1.59 | 0.93 | 2.70 | **2.57** | 1.24 | 5.31 | 1.13 | 0.61 | 2.10 |
| (BL: Brussels) | Wallonia | 478 | **1.35** | 1.03 | 1.76 |  |  |  |  |  |  | 1.29 | 0.70 | 2.35 | 1.54 | 0.78 | 3.03 | 1.79 | 0.95 | 3.38 |
| Occupation | Retired | 172 |  |  |  | **0.04** | 0.02 | 0.12 | 0.18 | 0.03 | 1.09 | 1.56 | 0.95 | 2.58 |  |  |  | 0.91 | 0.51 | 1.62 |
| (BL: Working) | At home | 88 |  |  |  | **0.00** | 0.00 | 0.00 | 0.83 | 0.16 | 4.41 | 0.84 | 0.49 | 1.44 |  |  |  | **1.85** | 1.23 | 2.80 |
|  | Unemployed | 76 |  |  |  | **0.06** | 0.02 | 0.20 | 0.83 | 0.28 | 2.47 | 1.41 | 0.67 | 2.95 |  |  |  | 1.35 | 0.82 | 2.21 |
|  | Further/fulltime education | 84 |  |  |  | 0.65 | 0.31 | 1.36 | **6.96** | 3.16 | 15.33 | 1.13 | 0.64 | 2.01 |  |  |  | 1.34 | 0.49 | 3.67 |
|  | Other | 70 |  |  |  | 0.46 | 0.21 | 1.02 | **0.08** | 0.01 | 0.57 | 0.91 | 0.52 | 1.59 |  |  |  | 1.19 | 0.76 | 1.88 |
| Age of participant | 25-44 years | 336 | 1.08 | 0.87 | 1.34 | 0.84 | 0.51 | 1.36 |  |  |  | **0.42** | 0.25 | 0.70 | **0.38** | 0.20 | 0.69 | 1.20 | 0.57 | 2.53 |
| (BL: 18-24 years) | 45-64 years | 368 | 1.03 | 0.84 | 1.28 | 1.13 | 0.68 | 1.88 |  |  |  | 0.68 | 0.37 | 1.26 | 1.08 | 0.59 | 1.98 | 1.20 | 0.56 | 2.55 |
|  | 65+ years | 96 | 1.27 | 0.93 | 1.73 | 0.96 | 0.23 | 3.99 |  |  |  | **0.34** | 0.14 | 0.84 | **0.38** | 0.16 | 0.91 | 1.50 | 0.56 | 4.02 |
| Education | Primary education | 36 |  |  |  | 4.27 | 0.37 | 49.03 |  |  |  |  |  |  |  |  |  | 1.26 | 0.48 | 3.31 |
| (BL: No formal education) | Secondary education | 498 |  |  |  | 38.13 | 18.74 | 77.60 |  |  |  |  |  |  |  |  |  | 1.82 | 0.71 | 4.64 |
|  | Higher education | 414 |  |  |  | 31.18 | 15.66 | 62.08 |  |  |  |  |  |  |  |  |  | 2.09 | 0.78 | 5.57 |
| Gender | Male | 426 |  |  |  |  |  |  |  |  |  |  |  |  | 1.62 | 0.90 | 2.90 |  |  |  |
| First or Second Day | Second Day | 479 |  |  |  |  |  |  |  |  |  |  |  |  | 0.68 | 0.43 | 1.10 |  |  |  |
| Household Size |  |  | **1.22** | 1.16 | 1.29 |  |  |  |  |  |  |  |  |  | 1.10 | 0.94 | 1.28 | **1.10** | 1.01 | 1.20 |
| Overdispersion + s.e. |  |  |  | **2.25** | 0.29 |  | **38.47** | 7.17 |  | **6.54** | 2.17 |  | **10.93** | 1.24 |  | **4.03** | 1.23 |  | **5.57** | 0.74 |
| Correlation + s.e. |  |  |  | 0.17 | 0.10 |  | 0.00 | 0.00 |  | 0.09 | 0.08 |  | 0.09 | 0.06 |  | 0.03 | 0.03 |  | **0.33** | 0.07 |
| * Excluded weekend |  |  |  |  |  |  |  |  |  |  |  |  |  |  |  |  |  |  |  |  |
| ** Excluded weekend & holiday | |  |  |  |  |  |  |  |  |  |  |  |  |  |  |  |  |  |  |  |

Table IV.3.2: Final weighted GEE model for the different location contacts for type IV diary. For each of the discrete variables, the baseline category is shown. In the third column, the number of observations in each category is given. The rate should be interpreted as a factor versus the baseline and the intercept rate and is given together with the 95% confidence limits (LCL, UCL).

| **Adults** |  | **N°** | **Daily Contacts** | | | **Weekly Contacts** | | | **Monthly Contacts** | | | **Few Times a Year** | | | **First Time Contacts** | | |
| --- | --- | --- | --- | --- | --- | --- | --- | --- | --- | --- | --- | --- | --- | --- | --- | --- | --- |
|  |  |  | Rate | LCL | UCL | Rate | LCL | UCL | Rate | LCL | UCL | Rate | LCL | UCL | Rate | LCL | UCL |
|  | (Intercept) |  | 5.39 |  |  | 2.32 |  |  | 4.55 |  |  | 1.15 |  |  | 1.60 |  |  |
| Day of week | Tuesday | 29 | 1.19 | 0.79 | 1.80 | 1.05 | 0.67 | 1.65 |  |  |  |  |  |  |  |  |  |
| (BL: Monday) | Wednesday | 37 | 1.03 | 0.73 | 1.44 | 0.84 | 0.60 | 1.16 |  |  |  |  |  |  |  |  |  |
|  | Thursday | 73 | 0.83 | 0.60 | 1.17 | 0.74 | 0.53 | 1.04 |  |  |  |  |  |  |  |  |  |
|  | Friday | 108 | 0.90 | 0.65 | 1.26 | 0.88 | 0.63 | 1.22 |  |  |  |  |  |  |  |  |  |
|  | Saturday | 96 | **0.35** | 0.27 | 0.46 | **0.32** | 0.24 | 0.43 |  |  |  |  |  |  |  |  |  |
|  | Sunday | 71 | **0.31** | 0.24 | 0.42 | **0.30** | 0.22 | 0.41 |  |  |  |  |  |  |  |  |  |
| Holiday period | Holiday | 24 | 0.92 | 0.73 | 1.17 |  |  |  |  |  |  | 0.82 | 0.64 | 1.04 |  |  |  |
| (BL: Regular weekday) | Holiday Tuesday | 42 |  |  |  |  |  |  |  |  |  |  |  |  |  |  |  |
|  | Holiday Wednesday | 64 |  |  |  |  |  |  |  |  |  |  |  |  |  |  |  |
|  | Holiday Thursday | 32 |  |  |  |  |  |  |  |  |  |  |  |  |  |  |  |
|  | Holiday Friday | 7 |  |  |  |  |  |  |  |  |  |  |  |  |  |  |  |
|  | Holiday Saturday | 148 |  |  |  |  |  |  |  |  |  |  |  |  |  |  |  |
|  | Holiday Sunday | 164 |  |  |  |  |  |  |  |  |  |  |  |  |  |  |  |
| Region | Flanders | 882 |  |  |  | **2.04** | 1.38 | 3.02 |  |  |  |  |  |  |  |  |  |
| (BL: Brussels) | Wallonia | 478 |  |  |  | **1.67** | 1.11 | 2.50 |  |  |  |  |  |  |  |  |  |
| Occupation | Retired | 172 | **0.18** | 0.12 | 0.25 | **0.31** | 0.22 | 0.43 | **0.58** | 0.34 | 0.99 |  |  |  |  |  |  |
| (BL: Working) | At home | 88 | **0.34** | 0.25 | 0.45 | **0.40** | 0.30 | 0.53 | 0.69 | 0.46 | 1.04 |  |  |  |  |  |  |
|  | Unemployed | 76 | **0.37** | 0.26 | 0.52 | **0.37** | 0.27 | 0.51 | 0.95 | 0.49 | 1.87 |  |  |  |  |  |  |
|  | Further/fulltime education | 84 | 0.98 | 0.66 | 1.44 | 1.19 | 0.84 | 1.68 | 1.00 | 0.63 | 1.61 |  |  |  |  |  |  |
|  | Other | 70 | 0.73 | 0.43 | 1.23 | 0.80 | 0.51 | 1.25 | 0.70 | 0.48 | 1.03 |  |  |  |  |  |  |
| Age of participant | 25-44 years | 336 |  |  |  |  |  |  | **0.70** | 0.50 | 0.96 |  |  |  |  |  |  |
| (BL: 18-24 years) | 45-64 years | 368 |  |  |  |  |  |  | 1.07 | 0.71 | 1.62 |  |  |  |  |  |  |
|  | 65+ years | 96 |  |  |  |  |  |  | 1.20 | 0.61 | 2.36 |  |  |  |  |  |  |
| Education | Primary education | 36 | 1.46 | 0.75 | 2.84 | 1.80 | 0.78 | 4.16 |  |  |  | 1.05 | 0.25 | 4.39 |  |  |  |
| (BL: No formal education) | Secondary education | 498 | **2.03** | 1.33 | 3.10 | **2.46** | 1.17 | 5.16 |  |  |  | 1.21 | 0.31 | 4.71 |  |  |  |
|  | Higher education | 414 | **1.66** | 1.09 | 2.51 | **2.18** | 1.03 | 4.64 |  |  |  | 1.67 | 0.43 | 6.52 |  |  |  |
| Gender | Male | 426 |  |  |  |  |  |  |  |  |  |  |  |  |  |  |  |
| First or Second Day | Second Day | 479 |  |  |  |  |  |  |  |  |  |  |  |  |  |  |  |
| Household Size |  |  |  |  |  |  |  |  |  |  |  | 1.09 | 1.00 | 1.18 |  |  |  |
| Overdispersion + s.e. |  |  |  | **5.79** | 0.57 |  | **7.72** | 0.92 |  | **6.96** | 0.85 |  | 4.83 | 0.40 |  | **6.64** | 0.86 |
| Correlation + s.e. |  |  |  | **0.23** | 0.06 |  | 0.02 | 0.03 |  | **0.14** | 0.04 |  | 0.18 | 0.05 |  | 0.10 | 0.08 |

Table IV.3.3: Final weighted GEE model for the different frequency contacts for type IV diary. For each of the discrete variables, the baseline category is shown. In the third column, the number of observations in each category is given. The rate should be interpreted as a factor versus the baseline and the intercept rate and is given together with the 95% confidence limits (LCL, UCL).

| **Adults** |  | **N°** | **Less than 5 min** | | | **5-15 min** | |  | **15 min-1 hour** | |  | **1-4 hours** | |  | **More than 4 hours** | | |
| --- | --- | --- | --- | --- | --- | --- | --- | --- | --- | --- | --- | --- | --- | --- | --- | --- | --- |
|  |  |  | Rate | LCL | UCL | Rate | LCL | UCL | Rate | LCL | UCL | Rate | LCL | UCL | Rate | LCL | UCL |
|  | (Intercept) |  | 0.32 |  |  | 2.07 |  |  | 0.44 |  |  | 4.17 |  |  | 5.31 |  |  |
| Day of week | Tuesday | 29 |  |  |  | 1.02 | 0.64 | 1.61 | 1.40 | 0.88 | 2.23 |  |  |  | 1.09 | 0.73 | 1.65 |
| (BL: Monday) | Wednesday | 37 |  |  |  | 0.90 | 0.62 | 1.31 | 1.22 | 0.78 | 1.91 |  |  |  | 0.86 | 0.61 | 1.20 |
|  | Thursday | 73 |  |  |  | 0.72 | 0.50 | 1.04 | 1.02 | 0.71 | 1.48 |  |  |  | 0.82 | 0.56 | 1.19 |
|  | Friday | 108 |  |  |  | 0.98 | 0.68 | 1.40 | 1.03 | 0.73 | 1.45 |  |  |  | 0.86 | 0.63 | 1.17 |
|  | Saturday | 96 |  |  |  | **0.36** | 0.26 | 0.50 | **0.46** | 0.33 | 0.65 |  |  |  | 0.72 | 0.50 | 1.03 |
|  | Sunday | 71 |  |  |  | **0.29** | 0.20 | 0.42 | **0.49** | 0.31 | 0.77 |  |  |  | **0.51** | 0.39 | 0.68 |
| Holiday period | Holiday | 24 | 0.82 | 0.64 | 1.07 |  |  |  | 1.47 | 0.90 | 2.40 | 0.86 | 0.71 | 1.03 |  |  |  |
| (BL: Regular weekday) | Holiday Tuesday | 42 |  |  |  |  |  |  | 0.66 | 0.30 | 1.48 |  |  |  |  |  |  |
|  | Holiday Wednesday | 64 |  |  |  |  |  |  | **0.47** | 0.24 | 0.93 |  |  |  |  |  |  |
|  | Holiday Thursday | 32 |  |  |  |  |  |  | 0.51 | 0.24 | 1.08 |  |  |  |  |  |  |
|  | Holiday Friday | 7 |  |  |  |  |  |  | 0.53 | 0.19 | 1.50 |  |  |  |  |  |  |
|  | Holiday Saturday | 148 |  |  |  |  |  |  | 0.73 | 0.41 | 1.32 |  |  |  |  |  |  |
|  | Holiday Sunday | 164 |  |  |  |  |  |  | 0.51 | 0.26 | 1.02 |  |  |  |  |  |  |
| Region | Flanders | 882 |  |  |  |  |  |  |  |  |  |  |  |  | **1.81** | 1.22 | 2.68 |
| (BL: Brussels) | Wallonia | 478 |  |  |  |  |  |  |  |  |  |  |  |  | **1.77** | 1.16 | 2.69 |
| Occupation | Retired | 172 | **0.47** | 0.29 | 0.78 | **0.34** | 0.24 | 0.49 | **0.42** | 0.30 | 0.60 | **0.59** | 0.44 | 0.81 | **0.35** | 0.22 | 0.55 |
| (BL: Working) | At home | 88 | 0.60 | 0.35 | 1.04 | **0.42** | 0.29 | 0.61 | **0.61** | 0.42 | 0.88 | **0.56** | 0.40 | 0.77 | **0.55** | 0.42 | 0.73 |
|  | Unemployed | 76 | **0.41** | 0.27 | 0.63 | **0.51** | 0.29 | 0.89 | **0.58** | 0.37 | 0.92 | 0.74 | 0.52 | 1.05 | **0.46** | 0.33 | 0.64 |
|  | Further/fulltime education | 84 | 0.90 | 0.56 | 1.45 | 1.06 | 0.70 | 1.60 | 0.88 | 0.59 | 1.31 | **1.64** | 1.22 | 2.22 | 1.05 | 0.76 | 1.46 |
|  | Other | 70 | 0.75 | 0.45 | 1.28 | 0.61 | 0.36 | 1.03 | 0.95 | 0.58 | 1.54 | 1.13 | 0.73 | 1.73 | **0.56** | 0.39 | 0.79 |
| Age of participant | 25-44 years | 336 |  |  |  |  |  |  |  |  |  |  |  |  |  |  |  |
| (BL: 18-24 years) | 45-64 years | 368 |  |  |  |  |  |  |  |  |  |  |  |  |  |  |  |
|  | 65+ years | 96 |  |  |  |  |  |  |  |  |  |  |  |  |  |  |  |
| Education | Primary education | 36 | **5.94** | 1.04 | 34.00 | **3.16** | 1.20 | 8.28 | **8.36** | 1.61 | 43.34 |  |  |  | **0.32** | 0.15 | 0.66 |
| (BL: No formal education) | Secondary education | 498 | **11.38** | 2.25 | 57.47 | **3.57** | 1.50 | 8.49 | **8.41** | 1.76 | 40.18 |  |  |  | 0.62 | 0.38 | 1.00 |
|  | Higher education | 414 | **12.84** | 2.53 | 65.16 | **3.29** | 1.38 | 7.82 | **7.72** | 1.62 | 36.79 |  |  |  | **0.59** | 0.36 | 0.96 |
| Gender | Male | 426 |  |  |  |  |  |  |  |  |  |  |  |  |  |  |  |
| First or Second Day | Second Day | 479 |  |  |  |  |  |  |  |  |  |  |  |  |  |  |  |
| Household Size |  |  |  |  |  |  |  |  | **1.16** | 1.08 | 1.26 |  |  |  |  |  |  |
| Overdispersion + s.e. |  |  |  | **7.73** | 0.98 |  | **7.86** | 1.07 |  | **5.81** | 0.52 |  | **5.87** | 0.67 |  | **5.26** | 0.64 |
| Correlation + s.e. |  |  |  | **0.17** | 0.06 |  | 0.03 | 0.04 |  | **0.14** | 0.04 |  | 0.08 | 0.05 |  | 0.05 | 0.07 |

Table IV.3.4: Final weighted GEE model for the different duration contacts for type IV diary. For each of the discrete variables, the baseline category is shown. In the third column, the number of observations in each category is given. The rate should be interpreted as a factor versus the baseline and the intercept rate and is given together with the 95% confidence limits (LCL, UCL).
